# Head-to-tail assembled borphyrins for solid-state lithium extraction

**DOI:** 10.1038/s41467-026-78080-0

**Published:** 2026-10-01

**Authors:** Ehsan Hamzehpoor, Seiya Ota, Suprio Chowdhury, Zongzhe Li, Ashley Yeung, Rhianna A. Parcon, Raksha Kandel, Brian O. Patrick, Mark J. MacLachlan

**Affiliations:** 1 Department of Chemistry, University of British Columbia, Vancouver, BC Canada; 2 Stewart Blusson Quantum Matter Institute, University of British Columbia, Vancouver, BC Canada; 3 Present Address: Department of Chemistry, University of Montreal, Montréal, QC Canada

**Keywords:** Chemical bonding, Chemical bonding, Molecular self-assembly, Self-assembly

## Abstract

Lithium is a critical mineral for advancing greener technologies. Extraction of lithium ions (Li⁺) from sea salt, minerals, and waste is important, but has been hindered by the lack of extraction methods that offer both high selectivity and strong lithium affinity. Here, we report a dioxaborole-linked, head-to-tail assembled macrocycle – termed borphyrin – featuring a rigid cavity with a 16-crown-4-like configuration. This molecule exhibits strong affinity for lithium and high selectivity over competing cations under dry solid-liquid extraction conditions, owing to its narrow binding cavity and planar macrocyclic core. Borphyrin enabled the extraction of lithium from crystalline solids of ether-insoluble salts, as well as selective extraction of Li⁺ from dried mixed-salt matrices containing alkali, alkaline earth and transition-metal salts. These results establish borphyrin as a potential molecular platform for selective lithium recognition and solid-liquid lithium extraction.

## Abstract (web summary)

Extraction of lithium ions (Li^+^) from sea salt, minerals, and waste is important, but the availability of extraction methods that offer both high selectivity and strong lithium affinity is limited. Here, the authors report a dioxaborole-linked, head-to-tail assembled macrocycle – termed borphyrin – featuring a rigid cavity with a 16-crown-4-like configuration.

## Introduction

Lithium-ion batteries account for over 87% of global lithium consumption, yet less than 5% of the lithium products are recycled. As a result, the demand for lithium production is met almost entirely through extraction from natural sources^1^. Its limited and geographically dispersed reserves raise concerns about future supply, making lithium a worldwide critical mineral^2^. The majority of the world’s lithium resources are not in ores or mines, but rather highly diluted lithium ions (Li^+^) in salt lakes and seawater, together comprising about 99% of all known lithium sources. Seawater alone contains an estimated 230 gigatons of lithium ion, compared to only 31 megatons in ore and brine deposits^3^. Resourcing the lithium from seawater and brines would unlock global access to this critical element for greener technologies, but remains a major challenge because of the very low Li^+^ concentration and the presence of large excesses of competing salt^4^.

Methods based on electrochemical techniques, membrane separation, and evaporative processes have been proposed, but they often lack either selectivity or efficiency^5^. These limitations arise because, in natural brines and seawater, Li^+^ (~0.1–0.2 ppm) must compete with sodium, magnesium, and calcium ions, which are present at concentrations several orders of magnitude higher^5^. Additionally, the high hydration energy of Li^+^ and the large lattice energy of its salts in the solid state further add a thermodynamic penalty and reduce separation efficiencies. Developing molecules and materials that can selectively bind to lithium is important for extracting, recovering, and purifying lithium salts^6^. In this context, molecular receptors that can selectively bind Li^+^ under competitive conditions provide useful platforms for evaluating the structural requirements for lithium recognition, even when the extraction format differs from direct aqueous lithium recovery.

More broadly, macrocyclic receptors have provided important design principles for lithium recognition, as summarized in recent reviews^7^. In seminal work, Cram and co-workers demonstrated that highly preorganized spherands can exhibit exceptionally strong and selective binding of small alkali metal cations, including Li^+^, highlighting the importance of rigid, size-matched cavities for lithium recognition^8,9^. Crown ethers (CEs), i.e., ring-shaped molecules that can bind guest ions and molecules in their interiors, have attracted extensive attention for their size-selective binding to alkali and alkaline earth metals, transition metals, and hydrogen-bonded organic molecules^10,11^. In CEs, oxygen donor atoms within their cavities, along with the flexibility of their polyether scaffold, enable binding to such a wide range of guests. However, high size-selectivity is required for the selective extraction of smaller cations like Li^+^ over larger cations like K^+^, Ca^2+^, and Cs^+^, which are less hydrated and are more favored in extractions following the Hofmeister bias^12^. Among CEs, 12-crown-4 and 14-crown-4 show the highest lithium affinity owing to the match of the ionic radius to their cavity size^13^, larger ions such as Na^+^ or K^+^, however, can form ML_2_ sandwich or M_2_L_3_ club sandwich complexes with these CEs (where M is the metal ion and L is the ligand), stabilized by the enthalpic gain of dual interactions with the metal center. Many modifications to crown-4 derivatives have been explored to prevent these sandwich complexes and improve Li^+^/Na^+^ extraction selectivity to ~1000^14,15^, but it came at the cost of lower binding affinities. Other macrocyclic systems, such as Gohil’s anthraquinone-linked macrocycle^16^, Ohto’s calix[4]arenes^17^, and Sessler’s calix[4]pyrrole hemispherands^18^, have addressed either selectivity (Li^+^/Na^+^ or Li^+^/Mg^2+^) or binding affinity. Nevertheless, there remains room to develop new macrocyclic design motifs that integrate rigid cavities for Li^+^, with strong Li^+^ affinity, high Li^+^ selectivity over other competing cations, and synthetic accessibility (Table S1).

Here we report a class of rigid and planar dioxaborole-linked macrocycles, synthesized on a multigram scale via head-to-tail boronic-ester condensation. These macrocycles, termed borphyrins, show strong, highly selective binding to lithium cations, enabling extraction from a broad range of lithium salts, including insoluble salts like LiF and Li_2_CO_3_, into organic solvents. They also retain Li^+^-selective extraction behavior in dried mixed-salt, including sea-salt mimics containing trace Li^+^ under dry solid–liquid conditions, establishing a proof-of-concept platform for selective lithium recognition.

## Results

### Head-to-tail assembly of borphyrins

Macrocycles are conventionally synthesized via template-assisted or thermodynamically controlled reversible reactions—like Schiff-base or boronic ester condensations—between multiple complementary precursors. These approaches often demand strict stoichiometric control and are prone to forming undesired oligomers or polymers. Head-to-tail (H2T) assembly of single ditopic precursors that contain both cross-reactive functional groups on the same synthon offers an alternative route to macrocycle formation. H2T strategies have been employed in the synthesis of G-quartets^19,20^, campestarenes^21^, cyanostars^22^, and metallomacrocycles^23,24^; yet, H2T assembly of aryl boronic esters is virtually unexplored (a few examples of aliphatic H2T-assembled sugars with boronic acids exist)^25–28^.

**Fig. 1:**
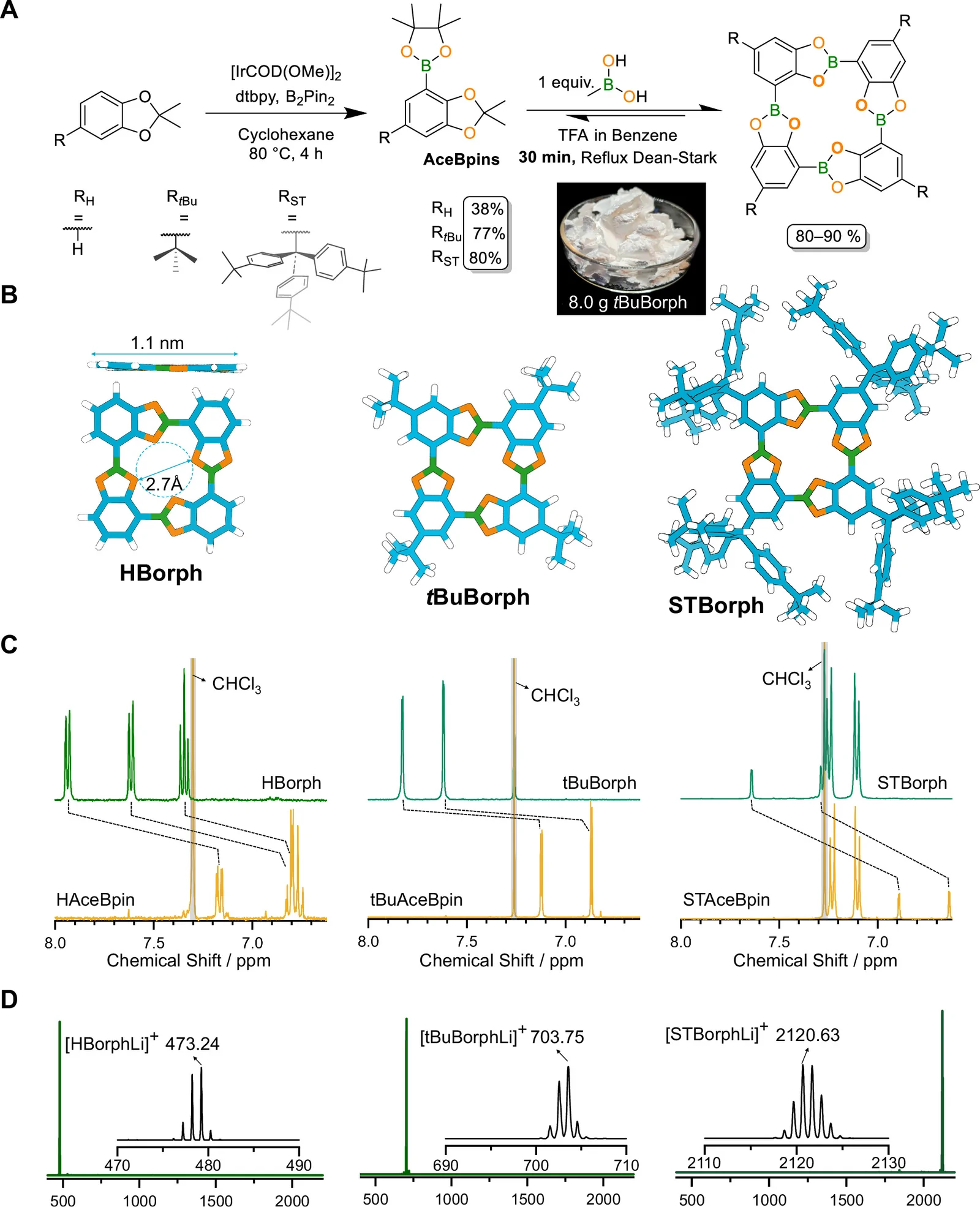
Synthesis and structure of borphyrins. **A** Synthesis of AceBpins via C–H borylation using 1 mol% [IrCOD(OMe)]_2_, 4,4′-di-tert-butyl-2,2′-bipyridine (dtbpy), and dry cyclohexane at 80°C, followed by borphyrin synthesis in a one-pot transesterification with methylboronic acid, trifluoroacetic acid (TFA), and benzene under Dean-Stark reflux for 30 min. **B** Molecular structures of borphyrins obtained from single-crystal X-ray diffraction data for HBorph (left; CCDC#2524674), tBuBorph (middle; CCDC#2514297), STBorph (right; CCDC#2513978). **C** Comparison of the ^1^H NMR of the borphyrins (top, green) with their corresponding AceBpins (bottom, orange) showing the downfield shift of the aromatic peaks upon macrocyclization. **D** MALDI-TOF mass spectra of the borphyrins spiked with 10 equiv. of LiBF_4_ solution. The inset is a zoomed-in spectrum on the corresponding peak region.

For more than a decade, we have been pursuing a molecule that we call the borphyrin (a portmanteau of boron and porphyrin) for its similarity to a porphyrin (Fig. 1)^29^. The theoretical precursor of the parent unsubstituted borphyrin molecule (HBorph) is the ditopic molecule of 3-boronic acid 1,2-dihydroxybenzene. Initial attempts to access this compound via BBr_3_-mediated demethylation of 3-boronic acid veratrole led to rapid protodeborylation of the starting material^29^. We found that the electron-rich nature of the catechol moiety, combined with its acidity, promotes fast autocatalytic protodeborylation^30^, thereby preventing the isolation of the desired precursor.

To circumvent this issue, catechol was protected with an acetonide group (110 g scale, 70% yield), followed by Ir-catalyzed borylation to produce the doubly protected intermediate AceBpin-1 (Fig. 1A). AceBpin-1 was then deprotected using dynamic transesterification with trifluoroacetic acid (TFA) and methylboronic acid^31^, affording HBorph with no detectable protodeborylation products. The borphyrin crystallized directly in the reaction mixture, was isolated by vacuum filtration, washed with acetonitrile, and further purified by sublimation under vacuum (2 × 10^−3 ^Torr, 300 °C), resulting in the isolated product in ~80% after sublimation. Owing to the poor solubility of HBorph at room temperature (soluble at 120 °C in 1,1,2,2-tetrachloroethane), we synthesized two sterically hindered borphyrins, STBorph and tBuBorph (Fig. 1A, B), using similar conditions from the corresponding acetonide precursors. AceBpin-2 was synthesized by Ir-catalyzed borylation on 20 g scale, and subsequently tBuBorph synthesis was scaled to 8 g of product without the need for chromatographic purification across the three-step sequence from commercially available catechol.

Investigation of the reaction conditions revealed that TFA concentration is critical to outcompete protodeborylation, with a minimum 2:1 ratio of benzene to TFA (≥100 equiv.) required for high yields (Fig. S1). This suggests that the rate-determining step (dioxaborole formation) is highly dependent on TFA concentration, while protodeborylation remains independent (close to zero-order with respect to [TFA]).

The doubly protected precursor AceBpin is critical for high-yielding reactions, as unprotected boronic acid (4-boronic acid 2,2-dimethylbenzodioxole) in the presence of TFA yields HBorph in moderate yields (~45%), with protodeborylated catechol as the major byproduct (Fig. S2).

Borphyrin formation was confirmed by spectroscopic and analytical techniques. ^1^H NMR spectra showed significant downfield shifts upon macrocyclization relative to the acetonide precursor (Fig. 1C), while maintaining the identical symmetry, number of peaks and H-H *J* coupling. MALDI-TOF mass spectrometry (Fig. 1D) confirmed the formation of the macrocyclic structure, with no evidence of other ring sizes (for HRMS structure validation, see Supplementary Information). IR spectra showed the disappearance of pinacol and acetonide CH_3_ bands at ~2900 cm⁻¹ and the emergence of characteristic dioxaborole stretches at ~1331, 1102, and 932 cm^−1^ (Fig. S3). Thermal stability of the macrocycles was assessed by thermogravimetric analysis (TGA) under both N_2_ and air, which showed all three borphyrins are thermally stable to at least 420 °C with HBorph and tBuBorph subliming above that temperature, while STBorph begins to decompose at >520 °C (Fig. S4).

Single-crystal X-ray diffraction of all three borphyrins (Fig. 1B), obtained via physical vapor transport (HBorph and tBuBorph) or slow solvent evaporation (STBorph), revealed square, planar macrocyclic cores (~1.1 nm per edge; average out-of-plane deviations of ~0.04 Å) with a cavity of 2.7 Å. Notably, the C_4h_ symmetry of the borphyrin core led to whole-molecule disorder in the diffraction data. For example, in the case of tBuBorph, a 50:50 mixture of rotationally disordered molecules was refined (Fig. S21). Wiberg bond index analysis of the simulated structure from density functional theory (DFT; Fig. S5A) revealed that the endo C-O and B-O bonds are slightly shorter than the exo, and Mulliken charge distribution (Fig. S5C) showed that the exo oxygens are slightly more negative as expected for being more distant from electron withdrawing B atom. This asymmetry is also reflected in the frontier molecular orbital topologies in which the highest occupied molecular orbital (HOMO) is localized on the endo oxygens (Fig. S6). DFT calculations in the gas phase suggest a slightly decreased bandgap from HAceBpin to HBorph, likely due to macrocyclic planarization of the boronic ester (Fig. S6). Nonetheless, the polar -C-B-O- bonds hinder electronic delocalization in borphyrins, as indicated by anisotropy of the induced current density (AICD, Fig. S7). tBuBorph and STBorph show diagnostic absorption peaks in their UV–vis absorption spectra at 301 nm and 306 nm, respectively (Fig. S8). Although the optical bandgap remains unchanged between borphyrins and corresponding AceBpins, borphyrins have narrower absorption bands, higher extinction coefficients and distinct vibrational fine structure as expected for their increased rigidity and symmetry.

### Lithium binding to borphyrins

**Fig. 2:**
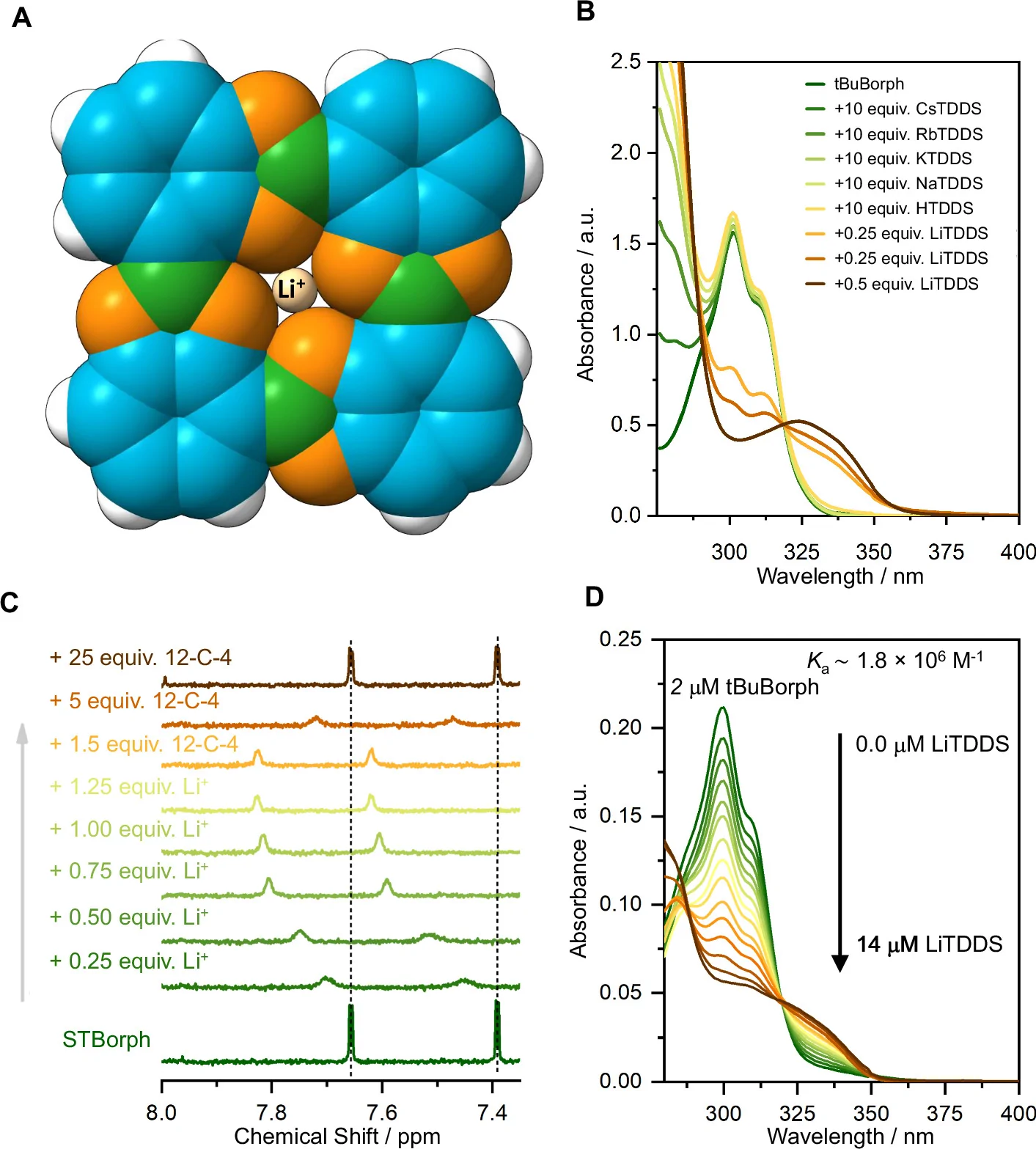
Lithium complexation of borphyrin. **A** Space-filling model of the lithium complexed borphyrins obtained from DFT calculation (B3LYP/6−31G(d)) showing the fit of Li^+^ ion inside the cavity. **B** UV–vis spectra of tBuBorph ([tBuBorph] = 15.5 μM) and sequential addition of MTDDS (M = Cs, Rb, K, Na, H, and Li; TDDS is (tridodecyloxy)benzenesulfonate). The equiv. values in the inset indicate the amount added at each step, not the cumulative concentration. At the end, 1.0 equiv. LiTDDS were in solution. **C** ^1^H NMR spectra of STBorph ([STBorph] = 0.23 mM in CD_2_Cl_2_, 400 MHz, 298 K) upon addition of LiB(C_6_F_5_)_4_ from a CD_2_Cl_2_ stock solution ([LiB(C_6_F_5_)_4_] = 4.6 mM), followed by competitive addition of 12-crown-4 (12-C-4). **D** UV–vis titration of tBuBorph ([tBuBorph] = 2 μM) in 1,4-dioxane with LiTDDS ([LiTDDS] = 0.1 mM stock solution in 1,4-dioxane). The *K*_a_ in the inset of the figure corresponds to global fit with a 1:1 binding model. Colors in (**B**–**D**) correspond to the species, equivalents, or concentrations indicated directly in each panel.

The inner cavity of borphyrin resembles the 16-crown-4 topology, but with a rigid and planar arrangement of oxygen donors with the optimal Rebek packing ratio for Li^+^ binding (R_Li+_/R_Borph_ = 0.56; Fig. 2A)^32^. It is noteworthy that the 16-crown-4 counterpart has a highly flexible backbone and has one of the smallest Li^+^ binding affinities and lithium selectivities among crown-4 derivatives.

DFT calculations suggest that Li^+^ can be accommodated within the borphyrin cavity with minimal distortion of the macrocyclic framework, whereas larger alkali-metal cations require more substantial structural deformation (Fig. S9). To obtain experimental insights into the binding selectivity of borphyrin, we studied the effect of different alkali metal cations on UV–vis absorption spectra of borphyrins. Sequential addition of soluble (tridodecyloxy)benzenesulfonate (TDDS) salts (M = Cs, Rb, K, Na) and the corresponding sulfonic acid (HTDDS) into a solution of tBuBorph in 1,4-dioxane resulted in no detectable change in the UV–vis spectrum, even in the presence of 10 equiv. each of CsTDDS, RbTDDS, KTDDS, NaTDDS, and HTDDS. However, upon addition of 0.25 equiv. of LiTDDS to this mixture, the peak at 301 nm drastically decreased, and a broad absorption spanning 300–360 nm emerged (Fig. 2B). The emerging UV–vis absorption is indicative of a new complex formation with complete consumption of free borphyrin observed upon addition of 1.0 equiv. of LiTDDS. The UV–vis (concentrated solution in Fig. 2B) and ^1^H NMR titration (Figs. 2C, S10A), as well as the Job’s plot analysis (Fig. S10B), suggest a 1:1 complexation stoichiometry (STBorph⊃Li^+^).

Stepwise addition of the LiB(C_6_F_5_)_4_ (in CD_2_Cl_2_-) into a CD_2_Cl_2_- solution of STBorph resulted in a continuous downfield shift and broadening of the STBorph aromatic peaks, up to 1.0 equiv. Li^+^, after which no further change was observed in the borphyrin chemical shift (Fig. 2C). Competitive binding study of 12-crown-4, a benchmark lithium ionophore, shows that nearly 25 equiv. is required to compete with the STBorph⊃Li^+^ complex, indicative of borphyrin’s relatively high lithium binding constant (*K*_a_).

UV–vis titration data of the borphyrins fit well to a 1:1 global binding model, giving calculated affinity constants of *K*ₐ = (1.8 ± 0.4) × 10^6 ^M^-1^ for tBuBorph titrated with LiTDDS in 1,4-dioxane (Fig. 2D and Fig. S11) and (3.9 ± 2.0) × 10^5 ^M^-1^ STBorph titrated with LiOTf in cyclopentyl methyl ether (CPME; Fig. S12), respectively. To quantify the Li^+^/Na^+^ affinity difference, we performed additional UV–vis titrations with Na^+^. Because the weak Na^+^ response requires high guest loadings, NaOTf delivered from a concentrated THF stock solution was used as a soluble Na^+^ source, allowing access to high Na^+^ concentrations without visible precipitation. In contrast to Li^+^, addition of Na^+^ produced only weak spectral changes at much higher guest concentrations. Fitting of three independent titrations to a 1:1 binding model gave apparent association constants in the 10^2 ^M^-1^ range (*K*_a_(Na^+^) = (0.9 ± 0.3) × 10^2 ^M^−1^; Fig. S13). We also evaluated a 2:1 binding model (representative of sandwich complexation or higher-order Na^+^ complexes); however, it gave poorly constrained and physically unrealistic parameters (Fig S13). We therefore use the 1:1 model as a simple apparent description of the weak Na^+^ response. Even using the largest fitted Na^+^ affinity, the measured STBorph–Li^+^ and STBorph–Na^+^ association constants differ by more than three orders of magnitude. Borphyrins’ *K*_a_ values are high relative to many reported macrocyclic lithium receptors (Table S1) with experimental ΔG = −35.7 ± 0.6 kJ mol^−1^ and −31.9 ± 1.4 kJ mol^−1^, for tBuBorph and STBorph, respectively. The lithium counterion had no noticeable impact on complexation. Ether-soluble lithium salts, including LiOTs, LiOTf, LiBF_4_, LiBPh_4_, and lithium methyl orange (LiMO), all showed similar binding responses upon addition to a solution of borphyrin in CPME (Fig. S14A–E). Moreover, etheric borphyrin solution was also able to extract lithium directly from solid crystals of a wide range of lithium salts, regardless of solubility, for example, LiCl and even poorly soluble salts such as Li_2_CO_3_ and LiF (the second-highest lattice energy among inorganic salts; Fig. S14), indicative of strong lithium binding. To further probe the effect of competing alkali metal ions, UV–vis experiments were performed by sequential addition of excess NaBF_4_ (500 equiv.) followed by LiBF_4_ (1 equiv.). Minimal spectral changes were observed upon addition of NaBF_4_ alone, whereas subsequent addition of LiBF_4_ resulted in pronounced perturbations, consistent with preferential lithium binding (Fig. S15). Solution-state lithium binding was further supported by ^1^H NMR spectroscopy, which revealed significant chemical shift changes in the aromatic region of STBorph upon addition of 1 equiv. of LiBArF (Fig. S16). Despite extensive crystallization trials, no single crystals of a borphyrin-containing Li^+^ complex suitable for X-ray diffraction were obtained (Table S3, Fig. S17). The proposed Li^+^-associated binding geometry is therefore supported by the combined UV–vis, NMR, mass spectrometric, and DFT evidence rather than direct crystallographic validation.

**Fig. 3:**
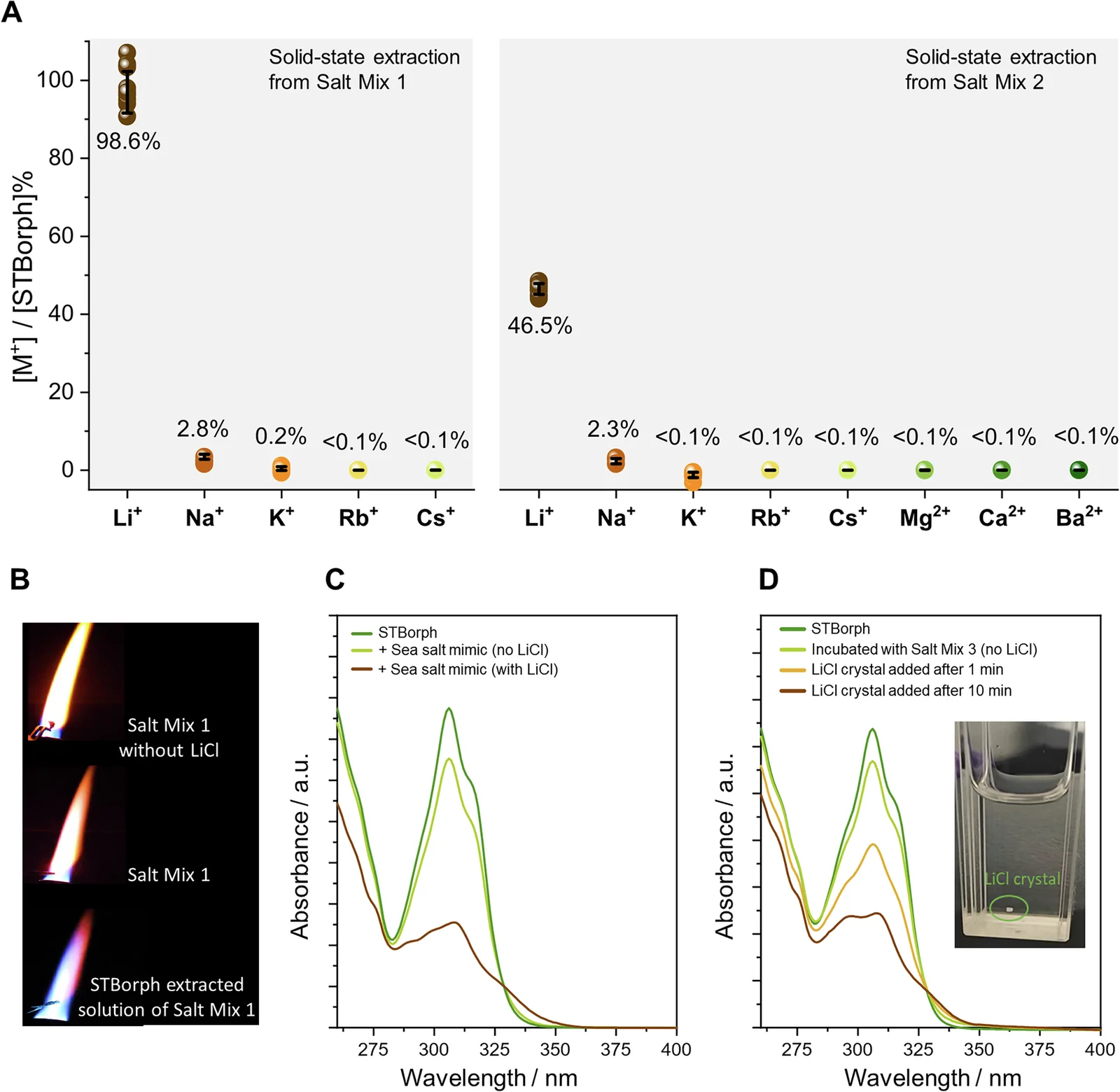
Lithium extraction from solid salt mixtures with borphyrin. **A** ICP-MS analysis of metal-ion extraction after incubation of STBorph ([STBorph] = 0.33 mM in CPME 10 mL) solution with a mixture of alkali metal chloride salt (left) and alkali metal and alkaline earth metal salts (Salt Mix 2; right). **B** Qualitative flame test of the extracted solution of STBorph upon exposure to Salt Mix 1, as well as control aqueous solutions of Salt Mix 1 in the absence or presence of LiCl. **C** UV–vis spectra of STBorph in CPME after exposure to a dried sea-salt mimic mixture without lithium chloride (light green) and with lithium chloride (maroon). **D** UV–vis spectra of STBorph after exposure to Salt Mix 3 in the absence of LiCl or in the presence of LiCl, measured 1 min and 10 min after addition of a solid LiCl crystal.

We then investigated the solid-state extraction of lithium using borphyrin from complex metal mixtures simulating sea salt. In the absence of borphyrin, LiCl extraction (from its crystalline solid) into cyclopentylmethyl ether (CPME; a green and water-immiscible organic solvent) was negligible (<0.1%). However, with borphyrin, near-quantitative lithium extraction (98.6% relative to loaded STBorph) was achieved from an equimolar alkali metal salt mixture (Salt Mix 1: LiCl, NaCl, KCl, RbCl, CsCl). When alkaline earth salts (Salt Mix 2: Salt Mix 1 + MgSO_4_, CaSO_4_, BaSO_4_) were included, extraction efficiency decreased to ~50%. This decrease is consistent with the greater complexity of the mixed solid-salt environment, where hydration, ion pairing, and salt availability may all influence extraction. We therefore interpret this result as a limitation of the current extraction system under more complex salt conditions, rather than attributing it to a single mechanism^33^. Notably, extraction of other cations remained negligible under these conditions (Fig. 3A).

Control experiments confirmed that none of the metal salts were soluble in CPME during the experiment (Fig. S18). Qualitative flame tests further illustrated successful lithium extraction. Control experiments of Salt Mix 1 showed a dominant yellow color due to the presence of NaCl, when a platinum wire was dipped in the aqueous solution of Salt Mix 1 and held within a flame. The red flame in the STBorph extraction solution is characteristic of pure lithium with minimal NaCl and KCl contribution (Fig. 3B). After liquid-liquid extraction of STBorph⊃Li⁺ to access lithium in the aqueous phase, borphyrin undergoes protodeborylation to its corresponding catechol, preventing recyclability for the studied derivatives (Fig. S19).

Borphyrins’ high lithium affinity and selectivity enabled extraction from a dried sea-salt mimic containing Li⁺ at an approximately 10,000-fold lower concentration than competing cations (Na^+^: K^+^: Mg^2+^: Ca^2+^: Li^+^ = 52500: 1900: 6250: 2000: 1), prepared from the corresponding chloride and sulfate salts (NaCl, KCl, MgSO_4_, CaSO_4_, and LiCl), Fig. 3C. Similar selectivity was observed with an extended salt mixture (Salt Mix 3 = Salt Mix 2 + Al(NO_3_)_3_, CoCO_3_, MnCO_3_, NH_4_Cl), relevant to lithium-ion battery recycling brines (Fig. 3D). To the best of our knowledge, few macrocyclic lithium receptors combine strong Li^+^ affinity with this level of Li^+^-selective recognition in such complex dried mixed salts. These results highlight borphyrins as a proof-of-concept molecular design motif for selective lithium recognition under competitive dry-salt conditions^1,34,35^. Despite the strong Li^+^ affinity and selectivity observed under dry binding and extraction conditions, controlled Li^+^ release remains a limitation of the present borphyrin design. Attempts to transfer Li^+^ from STBorph⊃Li^+^ into an aqueous phase led to protodeborylation of the borphyrin framework to the corresponding catechol, preventing straightforward recycling of the current derivatives (Fig. S19). Thus, while borphyrins provide a useful proof-of-concept design motif for selective Li^+^ recognition, future derivatives will require improved hydrolytic stability and controlled release to enable recyclable extraction cycles.

## Discussion

We have developed a class of macrocycles—borphyrins—via H2T assembly of boronic ester condensation from acetonide-protected ditopic precursors. These macrocycles exhibit high Li^+^ selectivity and strong binding affinity under dry conditions. We demonstrated selective Li^+^ extraction from alkali and alkaline earth salt mixtures, establishing borphyrins as a proof-of-concept molecular design motif for selective lithium recognition. At the same time, protodeborylation during protic or aqueous Li^+^ release prevents straightforward recycling of the current derivatives, highlighting hydrolytic stability and controlled release as key limitations of the present design. Further modulation of the borphyrin scaffold may enable more stable derivatives and provide design principles for future recyclable Li^+^-selective receptors.

## Methods

### General materials

All commercially available chemicals were purchased from Sigma-Aldrich, TCI, Cambridge Isotope Laboratories and Ambeed, and used without purification. Dry tetrahydrofuran (THF) and diethyl ether were collected from an Inert PureSolv MD5 purification system. Dry cyclohexane was obtained by distillation over CaH_2_. Deuterated solvents (CD_2_Cl_2_, chloroform-*d*, THF-d_8_, and 1,1,2,2-tetrachloroethane-*d*_2_) were purchased from Cambridge Isotope Laboratories and Sigma-Aldrich. Flash column chromatography was carried out using SiliCycle (230–400 mesh) silica gel as the stationary phase.

### Synthesis of borphyrins

HBorph was prepared from AceBpin-1 (50 mg, 0.181 mmol) and methylboronic acid (11 mg, 0.184 mmol) in a 20 mL round-bottom flask equipped with a Dean–Stark apparatus and an 8 mL trap. A premixed benzene/TFA solution (1:1 v/v, 15 mL) was added and the mixture was immediately heated to reflux. After approximately 40 min, the solvent collected in the Dean–Stark trap was drained and the remaining solvent was collected until the reaction mixture was dry. After cooling to room temperature, acetonitrile (5 mL) was added and the mixture was stirred for 5 min. The precipitated solid was collected by filtration, washed with acetonitrile (3 × 5 mL), and dried to give HBorph (19 mg, 0.040 mmol, 89%). Further purification by vacuum sublimation (2 × 10^-3 ^Torr, 300 °C) afforded HBorph (17 mg, 0.036 mmol, 80%).

For the multigram synthesis of tBuBorph, AceBpin-2 (17.600 g, 52.97 mmol) and methylboronic acid (3.170 g, 52.97 mmol) were placed in a 250 mL round-bottom flask equipped with a Dean–Stark apparatus and a 25 mL trap. A premixed benzene/TFA solution (2:1 v/v, 220 mL) was added and the mixture was immediately heated to reflux. The reaction mixture became opaque after approximately 10 min and was heated for 1 h. Solvent collected in the Dean–Stark trap was drained and the remaining solvent was collected until the reaction mixture was dry. After cooling to room temperature, acetonitrile (3 × 50 mL) was added and the mixture was stirred for 5 min. The solid was filtered, washed with acetonitrile (3 × 25 mL), and dried to afford tBuBorph as a white solid (8.010 g, 11.51 mmol, 87%), which was used without further purification.

STBorph was prepared from AceBpin-3 (300.3 mg, 0.456 mmol) and methylboronic acid (27.3 mg, 0.456 mmol) in a round-bottom flask equipped with a Dean–Stark apparatus and an 8 mL trap. A premixed benzene/TFA solution (1:1 v/v, 15 mL) was added and the mixture was immediately heated to reflux. After approximately 40 min, the solvent collected in the Dean–Stark trap was drained and the remaining solvent was collected until the reaction mixture was dry. After cooling to room temperature, acetonitrile (2 × 5 mL) was added and the mixture was stirred for 5 min. The precipitated solid was collected, washed with acetonitrile (3 × 10 mL), and dried to afford STBorph (213.5 mg, 0.101 mmol, 89%), which was used without further purification. Detailed syntheses and characterization of the AceBpin precursors and borphyrins are provided in the Supplementary Information.

### NMR spectroscopy

Nuclear magnetic resonance (NMR) experiments were recorded on Bruker AVIII HD 400 MHz, Bruker Avance 400 MHz, and Bruker Neo 600 MHz spectrometers. ^1^H and ^13^C NMR chemical shifts (*δ*) are reported in parts per million (ppm) relative to TMS using the residual solvent signal for calibration. *J* values are reported in Hz, and signal multiplicities are denoted as s (singlet), d (doublet), t (triplet), dd (doublet of doublets), td (triplet of doublets), ddd (doublet of doublets of doublets), and m (multiplet). All NMR spectra were obtained at room temperature unless otherwise noted. For the solution-state binding experiment shown in Fig. 2C, STBorph (0.23 mM in CD_2_Cl_2_, 298 K) was titrated with LiBArF from a 4.6 mM stock solution, followed by competitive addition of 12-crown-4.

### Mass spectrometry

Low-resolution mass spectra were collected on a Waters ZQ spectrometer equipped with an ESCI ion source. High-resolution mass spectra were collected on an ESI-TOF Waters Micromass LCT spectrometer or a Bruker Autoflex Speed LRF spectrometer. Matrix-assisted laser desorption/ionization time-of-flight (MALDI-TOF) mass spectra were obtained on a Bruker Biflex IV mass spectrometer equipped with a MALDI ion source in linear and reflectron modes. MALDI samples were prepared by the dried-droplet method from dioxane solutions of borphyrins and LiBF_4_.

### UV–vis spectroscopy and binding titrations

UV–vis absorption data were collected on a Cary 5000 UV–vis–NIR spectrophotometer using 10 mm pathlength quartz cuvettes. Lithium-binding titrations were conducted at 25 °C and monitored by UV–vis spectrophotometry. For the affinity measurements reported here, tBuBorph (2 μM) was titrated with LiTDDS from a 0.1 mM stock solution of LiTDDS in 1,4-dioxane, and STBorph (2 μM) was titrated with LiOTf from a 0.1 mM stock solution of LiOTf in CPME. All titrations were performed in triplicate. Isotherms were fitted by nonlinear least-squares analysis using the BindFit platform (supramolecular.org) with a 1:1 global binding model and the Nelder–Mead method to estimate association constants (*K*ₐ). For Na^+^ binding studies, STBorph (10 μM) in THF was titrated with NaOTf from a concentrated THF stock solution at 25 °C to access high Na^+^ concentrations while minimizing salt precipitation. Three independent titrations were fitted using a 1:1 binding model. A 2:1 model was also evaluated but produced poorly constrained parameters and was therefore not used to report the apparent Na^+^ affinity.

### Solid–liquid extraction experiments

For qualitative extraction from individual lithium salts, a freshly prepared solution of STBorph in CPME (approximately 3 μM; 3 mL) was transferred to a quartz cuvette to record the initial UV–vis spectrum. The solution was then transferred to a scintillation vial, approximately 0.1 mg of the lithium salt was added, and the vial was shaken for 30 s (approximately 5 min for the poorly soluble salts LiCl, LiF and Li_2_CO_3_). The solution was then returned to the cuvette and the UV–vis spectrum was recorded.

For the dried sea-salt-mimicking UV–vis experiment, a 5 g salt mixture was prepared with a Li: Na: K: Mg: Ca cation ratio of 0.2: 10500: 380: 1250: 400. A control mixture was prepared using the same cation ratios without lithium. Deionized water (approximately 10 mL) was added to each mixture to ensure homogeneous mixing, followed by drying at 160 °C overnight. STBorph (approximately 3.0 mg) was dissolved in CPME (5 mL), and a 1 mL aliquot was diluted to a final volume of 10 mL with CPME. After recording the initial spectrum, approximately 110 mg of the lithium-free salt mixture was added to the STBorph solution, the vial was shaken for 30 s, and the solution was passed through a 0.2 μm PTFE syringe filter before recording the spectrum. The procedure was repeated with incremental additions of 220 mg, 550 mg, and 1.10 g of salt. The entire experiment was then repeated under identical conditions with the lithium-containing salt mixture.

For the quantitative mixed-salt extraction experiments in Fig. 3A, STBorph (0.33 mM in CPME, 10 mL) was incubated with either an equimolar alkali-metal chloride mixture (Salt Mix 1: LiCl, NaCl, KCl, RbCl and CsCl) or Salt Mix 2 (Salt Mix 1 with MgSO_4_, CaSO_4_ and BaSO_4_). Control experiments evaluating salt transfer into CPME were performed by extracting dried salt mixtures with CPME (10 mL) under vigorous stirring for 10 s; the extraction was repeated twice. The combined organic phases were back-extracted with 2% HNO_3_ (5 mL × 2), and the aqueous extracts were analyzed by ICP-MS. Other ICP-MS extraction experiments were conducted under analogous conditions in the presence or absence of borphyrin. Extracted ion percentages were calculated relative to the total amount of each cation initially present in the salt mixture. Qualitative flame tests were performed using a platinum wire exposed to the extracted or control solutions.

### ICP-MS

Inductively coupled plasma mass spectrometry (ICP-MS) was performed using an Agilent 7850 ICP-MS. Calibration standards (0.1–10 ppm) were prepared for the metal ions quantified in each experiment, as applicable, from Li^+^, Na^+^, K^+^, Rb^+^, Cs^+^, Mg^2+^, Ca^2+^, Ba^2+^, Al^3+^, Co^2+^, and Mn^2+^ standards. Samples were prepared and diluted with 2% HNO_3_ to maintain analyte concentrations below 10 ppm.

### Thermogravimetric analysis

TGA was performed using a TG 209 F1 Libra thermogravimetric analyzer (NETZSCH) with a sensitivity of 0.1 μg. Samples of approximately 5 mg were analyzed under nitrogen or air at a flow rate of 50 mL min^−1^ from 30 to 650 °C using a heating rate of 10 °C min^−1^.

### Single-crystal X-ray diffraction

Single crystals of HBorph and tBuBorph were obtained by sublimation, whereas crystals of STBorph were obtained by slow evaporation of STBorph in toluene. Diffraction data for HBorph were collected at 296 K using Mo Kα radiation on a Bruker APEX-II CCD diffractometer. Data for tBuBorph and STBorph were collected at 100 K using Cu Kα radiation on Bruker D8 Venture and Bruker APEX-II area-detector diffractometers, respectively. The structures were solved and refined using Olex2/XT/XL workflows^36–38^ with full-matrix least-squares refinement on *F*^2^; non-hydrogen atoms were refined anisotropically and hydrogen atoms were placed geometrically and refined using a riding model. Full crystallographic collection and refinement details are provided in the Supplementary Information.

### Computational methods

Geometry calculations were performed using Gaussian 16^39^. Geometries were optimized in the gas phase using B3LYP with the 6–31G(d) basis set for H, C, B and O^40^. For Cs- and Rb-containing borphyrin complexes, the LANL2DZ basis set/effective core potential was used^41^. Anisotropy of the induced current density (AICD) maps were generated using AICD 3.0.4^42^, following established AICD analysis methods^43^.

## Supplementary information

Supplementary Information

Transparent Peer Review file

## Source data

Source Data

## Data Availability

The X-ray crystallographic coordinates for structures reported in this study have been deposited at the Cambridge Crystallographic Data Centre (CCDC) under deposition numbers 2524674 (HBorph), 2514297 (tBuBorph), and 2513978 (STBorph). These data can be obtained free of charge from the Cambridge Crystallographic Data Centre via www.ccdc.cam.ac.uk/data_request/cif. All other data supporting the findings of this study are provided in the manuscript or Supplementary Information. All data are available from the corresponding author upon request. Source data are provided with this paper.
