# Supplementary material for: Head-to-tail assembled borphyrins for solid-state lithium extraction
Source: Nat Commun. 2026 Oct 1;17:11270. doi: 10.1038/s41467-026-78080-0 (PMC13720209; doi:10.1038/s41467-026-78080-0)
Supplement: Supplementary file 1 [file 41467_2026_78080_MOESM1_ESM.pdf]

Supplementary Information

Head-to-tail assembled borphyrins for solid-state lithium extraction

Ehsan Hamzehpoor, Seiya Ota, Suprio Chowdhury, Zongzhe Li, Ashley Yeung, Rhianna A. Parcon, Raksha Kandel, Brian O. Patrick, Mark J. MacLachlan*

*Corresponding author: mmaclach@chem.ubc.ca

Materials and Methods

All commercially available chemicals were purchased from Sigma-Aldrich, TCI, Cambridge Isotope Laboratories and Ambeed, and used without purification. Dry tetrahydrofuran (THF) and diethyl ether were collected from an Inert PureSolv MD5 purification system. Dry cyclohexane was obtained by distillation over CaH_2. Deuterated solvents (CD_2Cl_2 (DCM-d_2), chloroform-*d*, and 1,1,2,2-tetrachloroethane-d_2) were purchased from Cambridge Isotope Laboratories and Sigma-Aldrich. Flash column chromatography was carried out using SiliCycle (230-400 mesh) silica gel as the stationary phase. AceBOH (used for reaction in Figure S2) was synthesized using a reported procedure[1].

Nuclear magnetic resonance (NMR) experiments were recorded on Bruker AVIII HD 400 MHz, Bruker Avance 400 MHz, and Bruker Neo 600 MHz spectrometers; ^1H and ^{13}C NMR chemical shifts (δ) are given in parts per million (ppm) relative to TMS, using the residual solvent signal for calibration. *J* values are reported in Hz, and signal multiplicity is denoted as *s* (singlet), *d* (doublet), *t* (triplet), *dd* (double of doublets), *td* (triplet of doublets), *ddd* (doublet of doublets of doublets), and *m* (multiplet). All NMR spectra were obtained at room temperature unless otherwise noted.

Low-resolution mass spectra (LRMS) were collected on a Waters ZQ spectrometer equipped with an ESCI ion source.

High-resolution mass spectra (HRMS) were collected on an ESI-TOF Waters Micromass LCT spectrometer or a MALDI-TOF Autoflex Speed LRF spectrometer.

Matrix assisted laser desorption/ionization (MALDI) mass spectra were obtained on a Bruker Biflex IV time-of-flight (TOF) mass spectrometer equipped with a MALDI ion source in both linear and reflection mode. MALDI sample was prepared with dried-droplet method from a dioxane solution of borphyrins and lithium $LiBF_4$.

UV–vis data were collected on a Cary 5000 UV–vis-NIR spectrophotometer using 10 mm pathlength quartz cuvettes. Lithium-binding titrations were conducted at 25 °C and monitored by UV–vis spectrophotometry. For the affinity measurements reported here, tBuBorph (2 μM) was titrated with LiTDDS from a 0.1 mM stock solution in 1,4-dioxane, and STBorph (2 μM) was titrated with LiOTf from a 0.1 mM stock solution in CPME. All titrations were performed in triplicate. Isotherms were fitted by nonlinear least-squares analysis using the BindFit platform (supramolecular.org) with a 1:1 global binding model and the Nelder–Mead method to estimate association constants (K_a). For Na^+ binding studies, STBorph (10 μM) in THF was titrated with NaOTf from a concentrated THF stock solution at 25 °C to access high Na^+ concentrations while minimizing salt precipitation. Three independent titrations were fitted using a 1:1 binding model. A 2:1 model was also evaluated but produced poorly constrained parameters and was therefore not used to report the apparent Na^+ affinity.

ICP-MS Experimental: Inductively coupled plasma mass spectrometry (ICP-MS) was performed using an Agilent 7850 ICP-MS. Calibration standards (0.1–10 ppm) were prepared for the metal ions quantified in each experiment, as applicable, from Li^+, Na^+, K^+, Rb^+, Cs^+, Mg^{2+}, Ca^{2+},

Ba^{2+}, Al^{3+}, Co^{2+}, and Mn^{2+} standards. All samples were prepared and diluted with 2% HNO_3 to ensure that analyte concentrations were below 10 ppm.

Thermogravimetric analysis (TGA): TGA was performed using a TG 209 F1 Libra thermogravimetric analyzer (NETZSCH), with a sensitivity of 0.1 μg. All tests were based on samples of ~5 mg, and conducted under nitrogen or air with a flow rate of 50 mL min^{-1}, and programmed for data collection between 30 and 650 °C, with a heating rate of 10 °C min^{-1}.

Computational methods: The Gaussian suite of programs G016.revC01 was used for all geometry calculations[2]. Geometries were optimized in gas phase with B3LYP with 6-31G(d) for all structures[3]. In case of Cs and Rb borphyrin complexes, B3LYP LanL2DZ[4]. AICD (anisotropy of the induced current density) graphs were calculated using the AICD 3.0.4 code from Dr. Z. Wang[5]. For a comprehensive review on AICD plots and interpretation see the review by D. Geuenich *et al.*[6].

Supplementary Text

Synthesis and Characterization

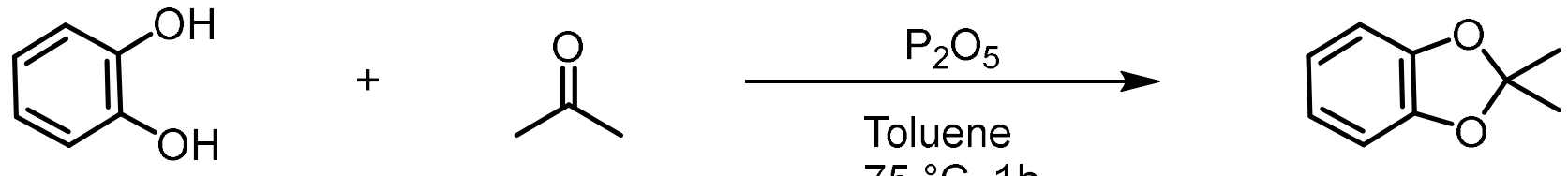

Ace-1

2,2-Dimethyl-1,3-benzodioxole (**Ace-1**): To a stirring mixture of catechol (110 g, 1.0 mol) and P_2O_5 (28.4 g, 0.2 mol) in toluene (500 mL; heated to 75 °C), dry acetone (150 mL, 2.05 mol) was added dropwise over 3 h, while adding aliquots of P_2O_5 (28.4 g, 0.2 mol) every 30 mins (a

total of 142 g P_2O_5 used). After the addition of acetone completed, the mixture was stirred at 75 °C for another 1 h. The organic phase layer was carefully decanted off into a flask and a 150 mL solution of NaOH (1M aq.) was added and stirred for 10 min. The organic layer was separated, washed with water (1 x 40 mL) and saturated NaCl (2 x 20 mL) and concentrated using a rotary evaporator (35 °C, 35 torr to evaporate toluene and excess acetone), to give a light yellow oil from which **Ace-1** was obtained by vacuum distillation (70-75 °C at 19 Torr) as a colourless liquid (91.5 g, 0.61 mol 61%). The ^{1}H and ^{13}C NMR spectra matched that of reported literature[7]. ^{1}H NMR (400 MHz, $CDCl_3$) δ 6.75 - 6.83 (m, 4H), 1.68 (s, 6H); ^{13}C NMR (101 MHz, $CDCl_3$) δ 147.3, 121.0, 117.4, 108.5, 25.9.

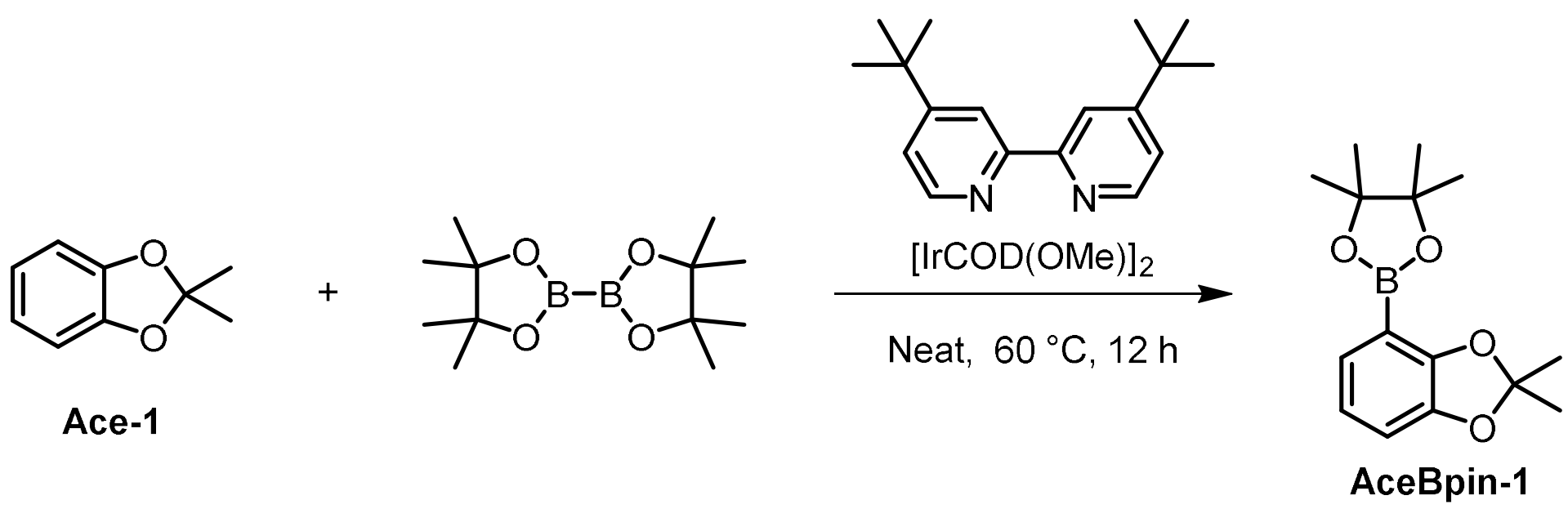

2-(2,2-dimethylbenzo[d][1,3]dioxol-4-yl)-4,4,5,5-tetramethyl-1,3,2-dioxaborolane (AceBpin-1): A mixture of **Ace-1** (2.50 g, 16.65 mmol; large excess) and bis(pinacolato)diboron (B_2Pin_2; 0.21 g, 0.83 mmol), was stirred at room temperature until all B_2Pin_2 dissolved (neat). The solution was purged with N_2 for 10 mins followed by addition of 4,4'-di-tert-butyl-2,2'-bipyridine (0.012 g, 41.6 µmol), and (1,5-cyclooctadiene) (methoxy)iridium(I) dimer $[IrCOD(OMe)]_2$ (0.011 g, 20.8 µmol). The mixture was then heated to 60 °C under N_2 for 12 h. The resulting dark brown solution was cooled down to room temperature, methanol (5 mL) was added dropwise to the reaction mixture, and the mixture was stirred for 10 mins. All volatiles were removed under vacuum using a rotary evaporator. The residue was purified by silica gel column chromatography

using a gradient of ethyl acetate/hexanes (0-10%) to afford **AceBpin-1** (0.175 g, 0.83 mmol, 38%) as a white crystalline solid. HRMS: $[C_{15}H_{21}BO_4H]^+$, m/z = 277.1611 Da (exp), 277.1606 Da (calc). ^{1}H NMR (400 MHz, $CDCl_3$) δ 7.15 (dd, J = 7.4, 1.7 Hz, 1H), 6.81-6.72 (m, 2H), 1.69 (s, 6H), 1.34 (s, 12H). ^{13}C NMR (101 MHz, $CDCl_3$) δ 152.8, 146.8, 127.3, 120.3, 117.3, 110.8, 83.6, 26.0, 24.8.

Note: the reaction yields bis(borylated) acetonide as the major product

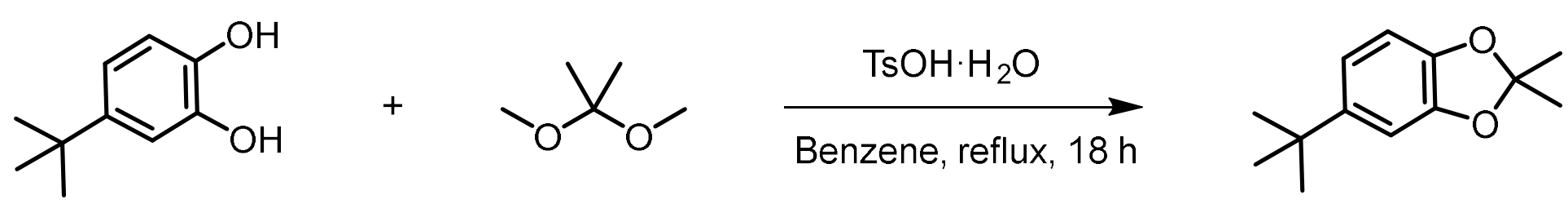

Ace-2

Small scale: 5-(tert-butyl)-2,2-Dimethyl-1,3-benzodioxole (**Ace-2**): To a mixture of 4-(tert-butyl)catechol (3.02 g, 18.2 mmol), and *p*-toluenesulfonic acid monohydrate (0.173 g, 0.910 mmol) was added 2,2-dimethoxypropane (22.3 mL, 182 mmol) and 50 mL of benzene. The mixture was heated at reflux in a Dean-Stark apparatus for 18 h. The reaction mixture was then cooled down to room temperature, after which 6 M NaOH (50 mL) and benzene (100 mL) were added to the mixture. After the mixture stirred for 30 mins, it was extracted three times (3 x 50 mL) with benzene, and all organic layers were combined, washed with saturated NaCl solution (20 mL) and dried with sodium sulfate. The combined organic layers were then dried under vacuum using a rotary evaporator. The resulting dark brown liquid was distilled under vacuum (115-120 °C, 19 Torr) to afford **Ace-2** (1.94 g, 9.44 mmol, 52% yield) as a colourless liquid. The ^{1}H NMR spectrum matched that of the reported literature[8]. ^{1}H NMR (400 MHz, $CDCl_3$) δ 6.83-6.62 (m, 3H), 1.67 (s, 6H), 1.28 (s, 9H). ^{13}C NMR (101 MHz, $CDCl_3$) δ 147.4, 145.2, 144.8, 117.6, 117.3, 107.6, 106.3, 34.7, 31.8, 26.1.

Large scale: 5-(tert-butyl)-2,2-Dimethyl-1,3-benzodioxole (**Ace-2**): Large scale **Ace-2** was synthesized using a reaction procedure similar to that of **Ace-1**. To a stirring mixture of 4-*tert*-

butylcatechol (45.0 g, 0.27 mol) and P_2O_5 (7.60 g, 54.0 mmol) in toluene (250 mL; heated to 75 °C), dry acetone (43.0 mL, 2.05 mol) was added dropwise over 3 h, while adding aliquots of P_2O_5 (7.80 g, 54.0 mmol) every 30 mins (a total of 38.4 g P_2O_5 used). After the addition of acetone completed, the mixture was stirred at 75 °C for another 1 h. The organic phase layer was carefully decanted off into a flask and a 150 mL solution of NaOH was added and stirred for 10 min. The organic layer was separated, washed with water (1 x 40 mL) and saturated NaCl (2 x 20 mL) and concentrated using a rotary evaporator (35 °C, 35 torr) to evaporate toluene and excess acetone), to give a light yellow oil from which **Ace-2** was obtained by vacuum distillation (115-120 °C, 19 Torr) as a colourless liquid (15.0 g, 0.073 mol, 27%). All characterisation data were identical to those mentioned above.

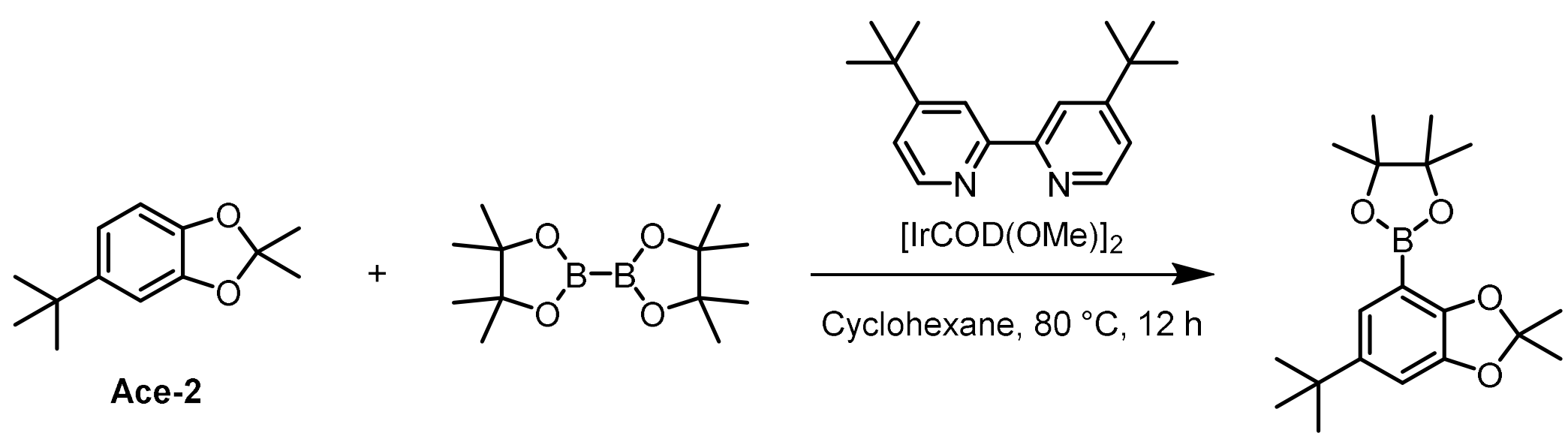

Ace-2 **AceBpin-2**

Small scale: 2-(6-(tert-butyl)-2,2-dimethylbenzo[d][1,3]dioxol-4-yl)-4,4,5,5-tetramethyl-1,3,2-dioxaborolane: (AceBpin-2): To a mixture of **Ace-2** (3.00 g, 14.54 mmol) and bis(pinacolato)diboron (4.43 g, 17.5 mmol), was added 4,4'-di-tert-butyl-2,2'-bipyridine (0.078 g, 0.29 mmol), and (1,5-cyclooctadiene)(methoxy)iridium(I) dimer $[IrCOD(OMe)]_2$ (0.074 g, 0.145 mmol), followed by 10 mL dry cyclohexane in a Schlenk flask. The reaction mixture was purged with N_2 and heated to 80 °C under N_2 overnight. The resulting black mixture was then cooled down to room temperature and methanol (5 mL) was added dropwise into the reaction mixture. After stirring the mixture at room temperature for 30 min, the solvents were removed using a rotary

evaporator. The resulting dark brown residue was dissolved in DCM (50 mL), the mixture was passed through a 1-inch silica gel plug, and the silica was washed with additional DCM (2 x 100 mL). The filtrate was collected and all volatiles were removed using a rotary evaporator, yielding crude mixture (3.71 g, 11.2 mmol; 77%). The crude mixture was then recrystallized from hot hexanes to afford pure **AceBpin-2** (2.15 g, 6.47 mmol, 45%). HRMS (ESI): $[C_{19}H_{29}BO_4H]^+$, m/z = 333.2237 Da (exp), 333.2159 Da (calc). ^{1}H NMR (400 MHz, $CDCl_3$) δ (ppm): 7.15 (s, 1H), 6.90 (s, 1H), 1.70 (s, 6H), 1.35 (s, 12H), 1.31 (s, 9H); ^{13}C NMR (75 MHz, $CDCl_3$) δ 150.9, 146.9, 143.7, 123.0, 117.4, 108.9, 83.6, 34.6, 31.7, 26.2, 24.9.

Large scale: 2-(6-(tert-butyl)-2,2-dimethylbenzo[d][1,3]dioxol-4-yl)-4,4,5,5-tetramethyl-1,3,2- dioxaborolane: (AceBpin-2): To a mixture of **Ace-2** (15.00 g, 72.7 mmol) and bis(pinacolato)diboron (36.93 g, 145.4 mmol), was added 4,4'-di-tert-butyl-2,2'-bipyridine (0.390 g, 1.45 mmol), and (1,5-cyclooctadiene)(methoxy)iridium(I) dimer $[IrCOD(OMe)]_2$ (0.368 g, 0.73 mmol), followed by 75 mL dry cyclohexane in a Schlenk flask. The reaction mixture was purged with N_2 and heated to 80 °C under N_2 overnight. The resulting black mixture was then cooled down to room temperature and methanol (25 mL) was added dropwise into the reaction mixture. After stirring the mixture at room temperature for 30 min, the solvents were removed using a rotary evaporator. The resulting dark brown residue was dissolved in DCM (150 mL), the mixture was passed through a 1-inch silica gel plug, and the silica was washed with additional DCM (2 x 250 mL). The filtrate was collected and all volatiles were removed using a rotary evaporator, yielding crude mixture (21.30 g, 64.11 mmol; 88%). The crude mixture was then recrystallized from hot hexanes to afford pure **AceBpin-2** (17.62 g, 53.0 mmol, 73%). All characterization data were identical to those mentioned above.

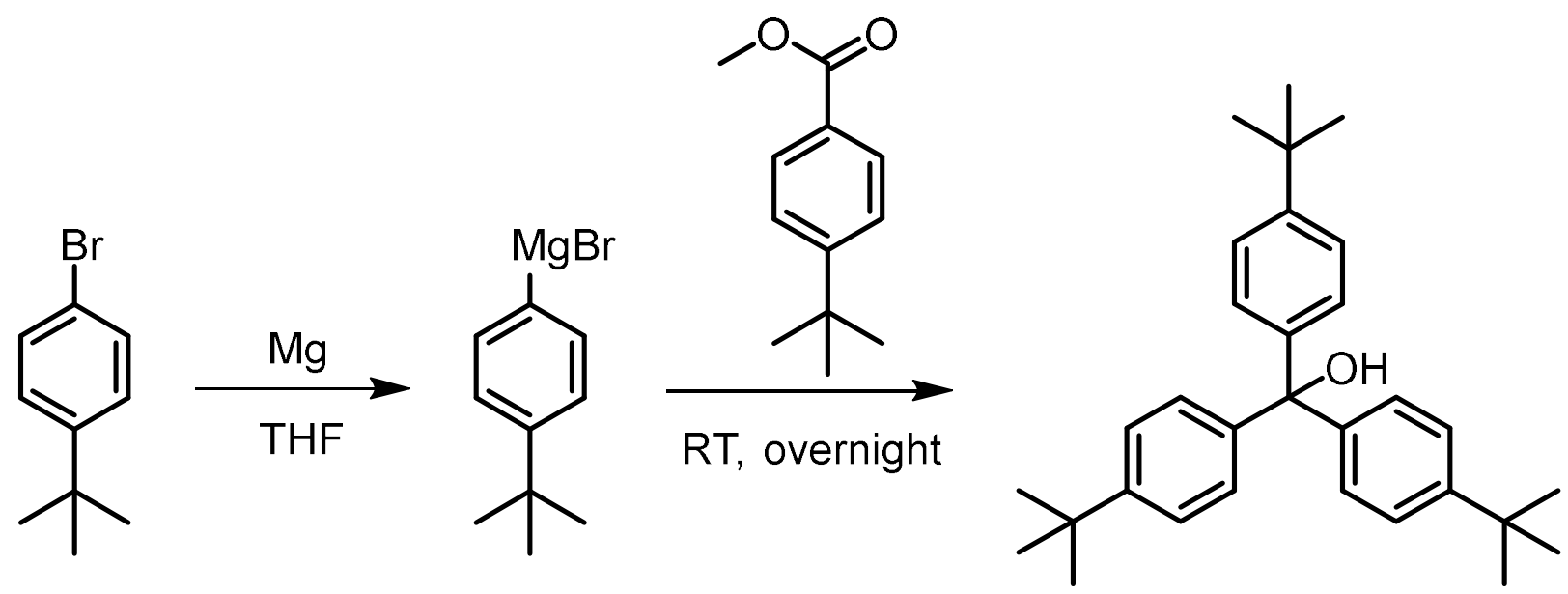

ST-OH

Tris-(4-tert-butyl-phenyl)-methanol (**ST-OH**): To a stirred suspension of magnesium (629 mg, 25.9 mmol) in dry THF (20 mL), pre-activated by washing sequentially with 1 M HCl (3 × 30 mL) and MeOH (5 × 30 mL), was added dropwise one-third of a solution of 1-bromo-4-tert-butylbenzene (4.49 mL, 25.9 mmol) in dry THF (20 mL). The mixture was heated vigorously with a heat gun until the reaction initiated, as indicated by the generation of bubbles without an external heat source. The remaining solution was then added dropwise over 15 min, and the reaction mixture was stirred for 1 h at room temperature with intermittent heat gun treatment, to keep a sustained reflux, indicating the formation of the Grignard reagent. Subsequently, a solution of methyl 4-tert-butylbenzoate (2 mL, 10.4 mmol) in dry THF (20 mL) was added dropwise over 20 minutes. The reaction mixture was stirred at room temperature for 18 h. After completion, a saturated aqueous solution of NH_4Cl (200 mL) was added, and the reaction mixture was extracted with CH_2Cl_2 (4 × 50 mL). The combined organic layer was dried over Na_2SO_4 and concentrated under reduced pressure. The crude product was purified by washing with hexane (3 × 10 mL) to afford **ST-OH** as a white powder (3.17 g, 7.38 mmol, 71%). ^{1}H and ^{13}C NMR spectra were in agreement with literature values[9]. ^{1}H NMR ($CDCl_3$, 400 MHz): δ 1.31 (*s*, 27H), 7.19 (*dd*, J = 2.1, 8.7 Hz, 6H), 7.32 (*dd*, J = 2.4, 8.4 Hz, 6H). The OH peak was not observed due to broadening; ^{13}C NMR (101 MHz, $CDCl_3$) δ 150.0, 144.4, 127.7, 124.8, 81.7, 34.6, 31.5.

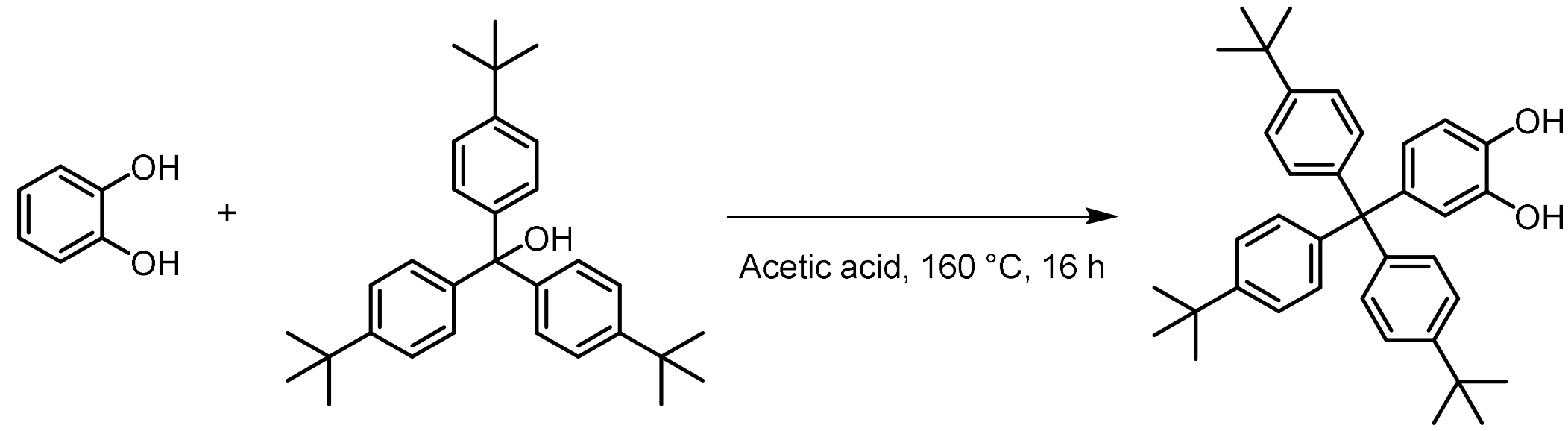

ST-OH ST-CAT

2,2-Dimethyl-5- tris-(4-tert-butyl-phenyl)-1,2-benzenediol (ST-CAT): Acetic acid (5 mL) was added to a mixture of **ST-OH** (2.00 g, 4.68 mmol) and catechol (773 mg, 7.03 mmol) in a thick-walled, high-pressure tube, and the vessel was closed and heated at 160 °C with stirring for 16 h. Upon cooling to room temperature, water (15 mL) was added, resulting in the formation of a white precipitate. The precipitate was collected by vacuum filtration and washed with water (3 × 30 mL) until the filtrate was neutral. The solid was suspended in CH_2Cl_2 (10 mL), and hexanes (20 mL) were added to induce precipitation. The resulting white solid was collected by vacuum filtration, affording the product **ST-CAT** as a white solid (1.88 g, 3.61 mmol, 77% yield). ^{1}H NMR (400 MHz, $CDCl_3$) δ 7.23 (*d*, *J* = 8.5 Hz, 6H), 7.08 (*d*, *J* = 8.5 Hz, 6H), 6.74 (*d*, *J* = 8.4 Hz, 1H), 6.69 (*d*, *J* = 2.1 Hz, 1H), 6.63 (*dd*, *J* = 8.4, 2.2 Hz, 1H), 5.05 (br, 1H), 4.86 (br, 1H), 1.30 (s, 27H); ^{13}C NMR (101 MHz, $CDCl_3$) δ 148.5, 144.1, 142.0, 141.6, 140.8, 130.9, 124.5, 124.2, 118.9, 114.2, 63.3, 34.4, 31.5. HRMS (ESI): $[C_{37}H_{43}O_2]^-$, *m/z* = 519.3272 Da (exp), 519.3263 Da (calc).

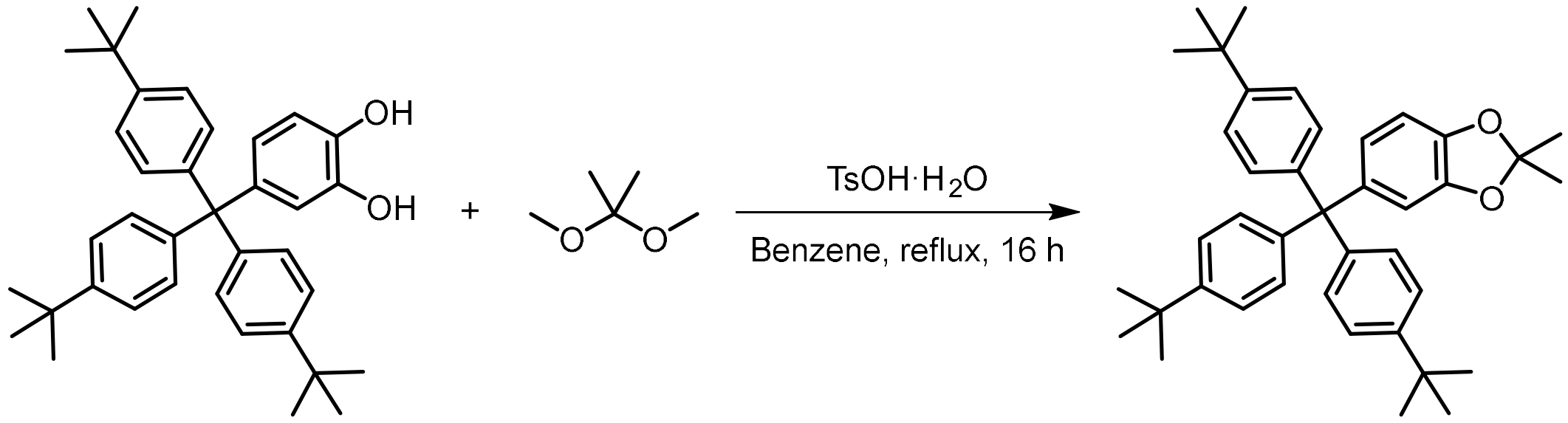

ST-CAT Ace-3

2,2-Dimethyl-5- tris-(4-tert-butyl-phenyl)- 1,3-benzodioxole (**Ace-3**): To a mixture of **ST-CAT** (1.50 g, 2.88 mmol) and *p*-toluenesulfonic acid monohydrate (50.0 mg, 0.290 mmol) was

added 2,2-dimethoxypropane (1.8 mL, 14.7 mmol) and benzene (60 mL). The reaction mixture was heated to reflux using a Dean-Stark apparatus for 16 h. After completion, the reaction mixture was cooled to room temperature, and the solvent was removed under reduced pressure, affording a brown solid. The crude brown solid was redissolved in CH_2Cl_2 (30 mL) and washed with water (3 × 30 mL). The organic layer was separated, dried over anhydrous $MgSO_4$, and concentrated under reduced pressure to give an orange solid. The orange solid was suspended in CH_2Cl_2 (10 mL), and hexanes (20 mL) were added to induce precipitation. The resulting white solid was collected by vacuum filtration, affording product **Ace-3** as a white solid (1.49 g, 2.66 mmol, 92% yield). ^{1}H NMR (400 MHz, $CDCl_3$) δ 7.24 (*d*, J = 8.6 Hz, 6H), 7.09 (*d*, J = 8.6 Hz, 6H), 6.60 (*d*, J = 6.5 Hz, 3H), 1.67 (*s*, 6H), 1.31 (*s*, 27H); ^{13}C {^{1}H} NMR (101 MHz, $CDCl_3$) δ 148.5, 146.6, 145.3, 144.2, 141.0, 130.9, 124.5, 124.2, 117.7, 112.1, 106.8, 63.6, 34.4, 31.5, 26.1. HRMS (APCI): $[C_{40}H_{48}O_2H]^+$, m/z = 561.3726 Da (exp), 561.3733 Da (calc).

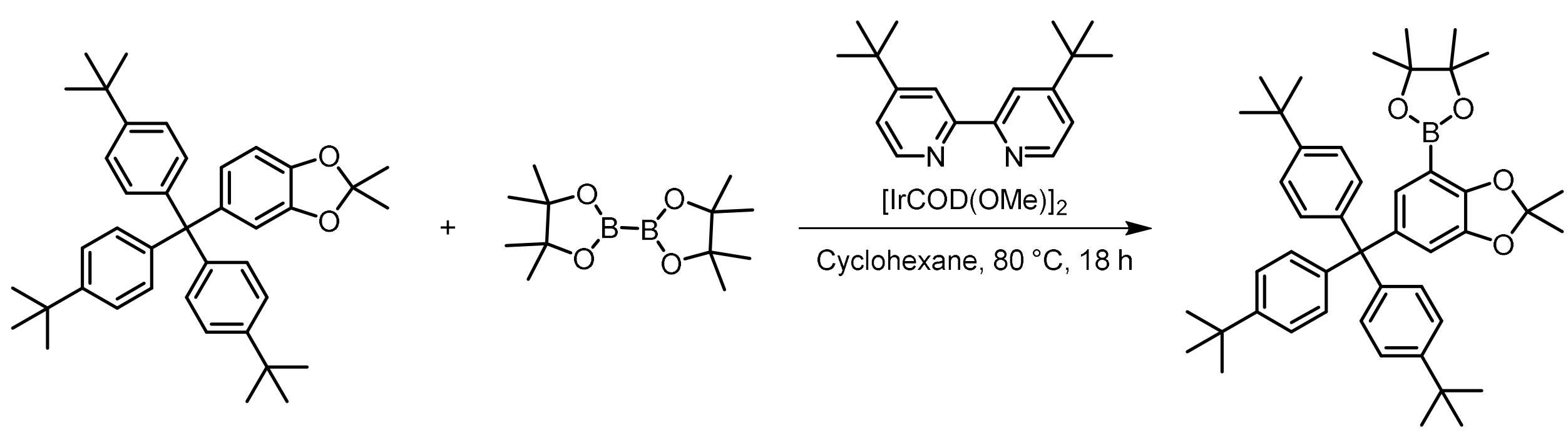

Ace-3 AceBpin-3

2-(2,2-dimethyl-6-(tris(4-(tert-butyl)phenyl)methyl)benzo[d][1,3]dioxol-4-yl)-4,4,5,5-tetramethyl-1,3,2-dioxaborolane (AceBpin-3): To a 20 mL Schlenk flask, **Ace-3** (500 mg, 0.892 mmol), B_2Pin_2 (227 mg, 0.894 mmol), 4,4'-di-tert-butyl-2,2'-dipyridyl (4.8 mg, 0.0179 mmol), and (1,5-cyclooctadiene)-(methoxy) iridium(I) dimer (5.9 mg, 0.00890 mmol) were added, followed by dry cyclohexane (5 mL) in a Schlenk flask. The reaction mixture was purged with N_2

and heated to 80 °C under N_2 overnight. The resulting black mixture was then cooled down to room temperature, and methanol (5 mL) was added dropwise into the reaction mixture. After stirring the mixture at room temperature for 30 min, the solvents were removed using a rotary evaporator. The resulting dark brown residue was dissolved in DCM (30 mL), the mixture was passed through a 1-inch silica gel plug, and the silica was washed with additional DCM (2 x 50 mL). The filtrate was collected and all volatiles were removed using a rotary evaporator, yielding crude product. The crude mixture was suspended in CH_2Cl_2 (10 mL), and hexanes (20 mL) were added to induce precipitation. The resulting white solid was collected by vacuum filtration, affording product **AceBpin-3** as a white solid (472 mg, 0.687 mmol, 77% yield). ^{1}H NMR (400 MHz, $CDCl_3$) δ 7.23 (*d*, J = 8.4 Hz, 6H), 7.11 (*d*, J = 8.4 Hz, 6H), 6.89 (*d*, J = 1.5 Hz, 1H), 6.64 (*d*, J = 1.5 Hz, 1H), 1.68 (*s*, 6H), 1.31 (*s*, 27H), 1.25 (*s*, 12H); ^{13}C {^{1}H} NMR (101 MHz, $CDCl_3$) δ 150.9, 148.4, 146.1, 144.2, 140.0, 131.0, 130.2, 124.1, 117.6, 114.6, 83.5, 63.6, 34.4, 31.5, 26.2, 24.9. HRMS (ESI): $[C_{44}H_{59}BO_4]^+$, m/z = 685.4434 Da (exp), 685.4542 Da (calc).

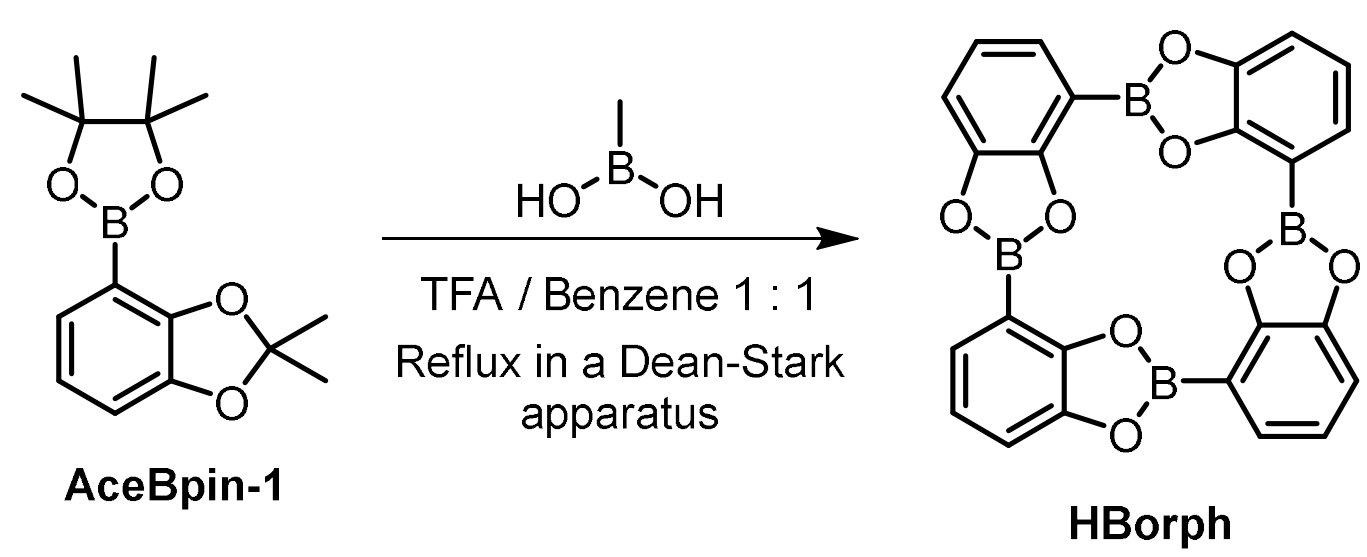

4,6:10,12:16,18:22,24-tetraepoxytetrabenzo[c,g,k,o][1,5,9,13]tetraoxa[2,6,10,14]tetraboracyclohexa-decine (HBorph). A mixture of **AceBpin-1** (0.050 g, 0.181 mmol), and methyl boronic acid (0.011 g, 0.184 mmol) was added to a 20 mL round bottom flask equipped with a Dean-Stark apparatus with an 8 mL trap. A premixed benzene/TFA (1:1 v/v, 15 mL) solution was added and immediately

heated to reflux. After ~40 min, the solvent collected in the Dean–Stark trap was completely drained and the remaining solvent in the flask was further collected in the trap until the reaction mixture was dried out. The white solid residue was cooled to room temperature and then acetonitrile (5 mL) was added and the mixture was stirred for 5 min, resulting in the precipitation of a white solid. The solid was filtered and washed with additional acetonitrile (3 x 5 mL) and dried on filter paper, resulting in **HBorph** as a white solid (0.019 g, 0.040 mmol, 89%), which was further purified by vacuum sublimation (2 × 10^{-3} Torr, 300 °C), resulting in the isolated product in (0.017 g, 0.036 mmol, ~80%). ^{1}H NMR (400 MHz, CD_2Cl_2, 100 °C) δ 7.94 (dd, J = 7.6, 1.3 Hz, 1H), 7.62 (dd, J = 7.9, 1.3 Hz, 1H), 7.35 (t, J = 7.8 Hz, 1H). ^{13}C NMR (obtained from HSQC and HMBC spectra due to low solubility of the **HBorph** even at elevated temperatures) δ 177.8, 171.0, 152.1, 145.9, 139.4, 134.2. HRMS (FD): $[C_{24}H_{12}B_4O_8]^+$, m/z = 472.0912 Da (exp), 472.0904 Da (calc).

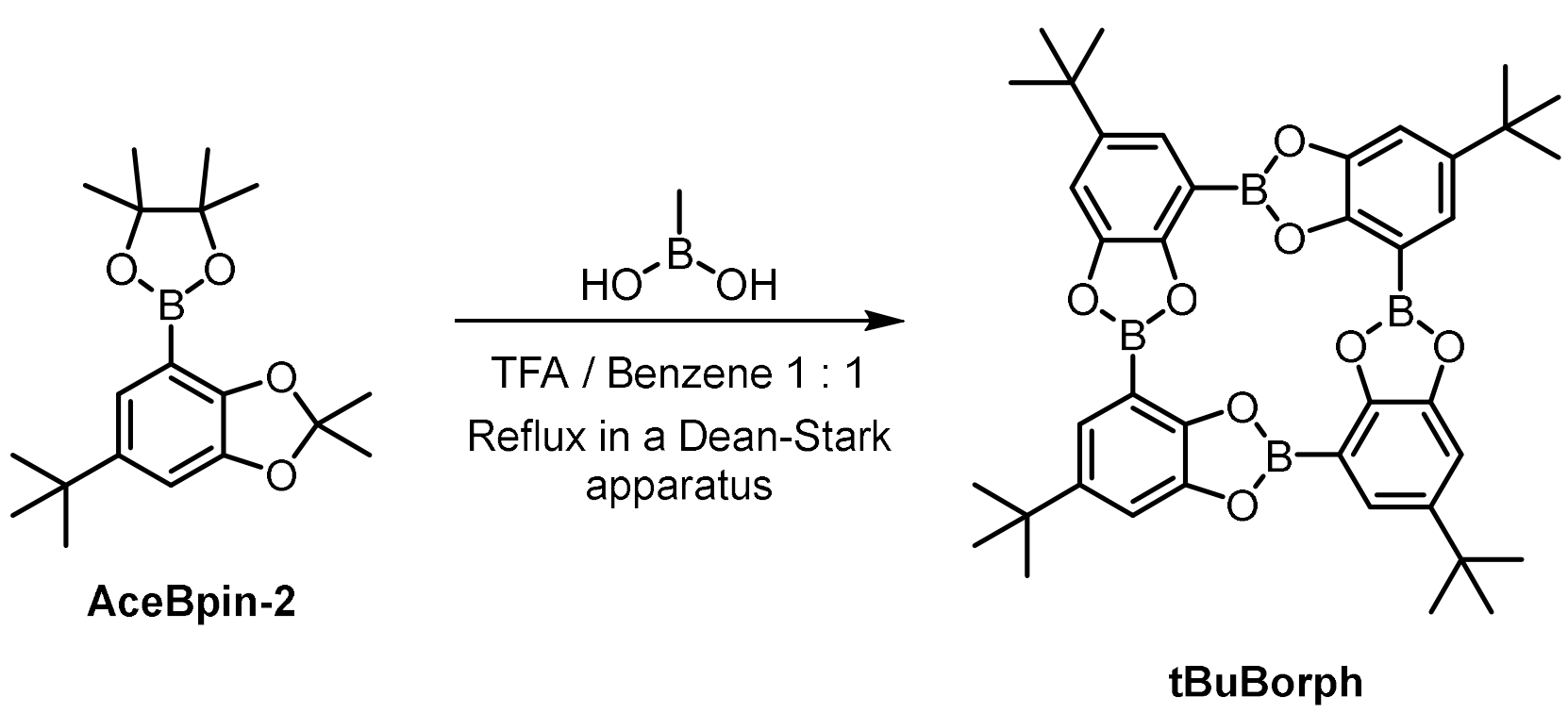

Small scale tBuBorph synthesis:

2,8,14,20-tetra-tert-butyl-4,6:10,12:16,18:22,24tetraepoxytetrabenzo[c,g,k,o][1,5,9,13] tetraoxa[2,6,10,14]tetraboracyclohexadecine(tBuBorph). AceBpin-2 (200.0 mg, 0.602 mmol) and methylboronic acid (36 mg, 0.602 mmol) were placed in a round-bottom flask equipped with a Dean-Stark apparatus with an 8 mL trap. A premixed benzene/TFA (1:1 v/v, 15 mL) solution was added and immediately heated to reflux. After ~40 min, the solvent collected in the Dean–

Stark trap was completely drained and the remaining solvent in the flask was further collected in the trap until the reaction mixture was dried out. The white solid residue was allowed to cool to room temperature to which acetonitrile (2 x 5 mL) was added and stirred for 5 min, resulting in the precipitation of a white solid. The solid was collected and washed with additional acetonitrile (3 x 10 mL) and dried on filter paper, resulting in **tBuBorph** as a white solid (86 mg, 0.123 mmol, 82%), which was used without further purification. ^{1}H NMR (400 MHz, $CDCl_3$) δ 7.84 (d, J = 2.0 Hz, 1H), 7.63 (d, J = 2.0 Hz, 1H), 1.46 (s, 9H). ^{13}C NMR (101 MHz, $CDCl_3$) δ 152.4, 147.8, 146.7, 125.4, 114.4, 109.8, 35.2, 31.9. HRMS (FD): $[C_{40}H_{44}B_4O_8]^+$, m/z = 696.3377 Da (exp), 696.3408 Da (calc).

Large scale tBuBorph synthesis: AceBpin-2 (17.600 g, 52.970 mmol) and methylboronic acid (3.170 g, 52.970 mmol) were placed in a 250 mL round-bottom flask equipped with a Dean-Stark apparatus with a 25 mL trap. A premixed benzene/TFA (2:1 v/v, 220 mL) solution was added and immediately heated to reflux. The reaction mixture turns opaque in 10 min, and the slurry was heated for 1 h after which, the solvent collected in the Dean–Stark trap was completely drained and the remaining solvent in the flask was further collected in the trap and drained until the reaction mixture was dried out. The white solid residue was allowed to cool to room temperature to which acetonitrile (3 x 50 mL) was added and stirred for 5 min. The solid was filtered and washed with additional acetonitrile (3 x 25 mL) and dried on filter paper, resulting in **tBuBorph** as a white solid (8.010 g, 11.51 mmol, 87%), which was used without further purification. The spectroscopic characterization was identical to that of the small scale **tBuBorph**.

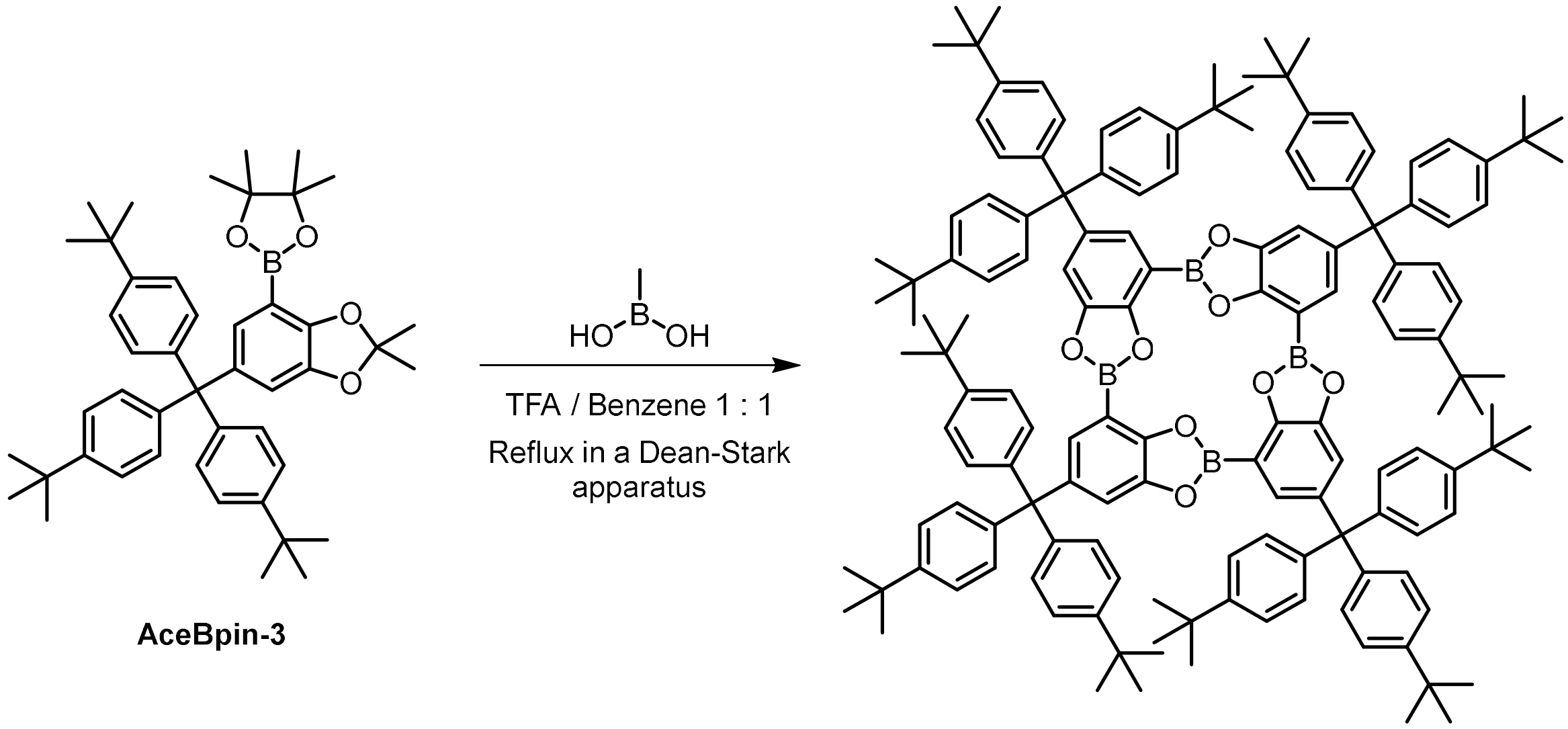

AceBpin-3 **STBorph**

2,8,14,20-tetrakis(tris(4-(tert-butyl)phenyl)methyl)-4,6:10,12:16,18:22,24-tetraepoxytetrabenzo [c,g,k,o][1,5,9,13]tetraoxa[2,6,10,14]tetraboracyclohexadecine (STBorph). **AceBpin-3** (300.3 mg, 0.456 mmol) and methylboronic acid (27.3 mg, 0.456 mmol) were placed in a round-bottom flask equipped with a Dean-Stark apparatus with an 8 mL trap. A premixed benzene/TFA (1:1 v/v, 15 mL) solution was added and immediately heated to reflux. After ~40 min, the solvent collected in the Dean–Stark trap was completely drained and the remaining solvent in the flask was further collected in the trap until the reaction mixture was dried out. The oily residue was allowed to cool to room temperature to which acetonitrile (2 x 5 mL) was added and the mixture was stirred for 5 min, resulting in the precipitation of a white solid. The solid was collected and washed with additional acetonitrile (3 x 10 mL) and dried on filter paper, resulting in **STBorph** as a white solid (213.5 mg, 0.101 mmol, 89%), which was used without further purification. ^{1}H NMR (400 MHz, $CDCl_3$) δ 7.63 (*d*, J = 1.9 Hz, 1H), 7.28 (*d*, J = 1.9 Hz, 1H), 7.24 (*d*, J = 8.6 Hz, 6H), 7.10 (*d*, J = 8.6 Hz, 6H), 1.29 (s, 27H); ^{13}C NMR (101 MHz, $CDCl_3$) δ 152.7, 148.7, 147.2, 143.8, 143.0, 131.8, 130.9, 124.4, 119.9, 63.9, 34.5, 31.5 (One aromatic

carbon signal was not observed, presumably due to quadrupolar broadening from the adjacent boron atom). HRMS (ESI): $[C_{148}H_{164}B_4O_8]^+$, *m*/*z* = 2113.3185 Da (exp), 2113.2868 Da (calc). $[C_{148}H_{164}B_4O_8Li]^+$, *m*/*z* = 2121.3036 Da (exp), 2121.2992 Da (calc).

Comparison of macrocyclic lithium ionophores

Table S1. Survey of macrocycle-based lithium ionophores compared to this work.

Ionophore	**Guest salt**	**Complex Equiv.**	**Solvent**	**Method**	**Binding constant K_a**	**Selectivity**	**Ref.**
$A(AA)_2A$	LiPic	1:1	$CDCl_3$ saturated with D_2O	NMR	$> 7 \times 10^{16}$ M^{-1}	Li:Na 580:1	10
1a	$LiClO_4$	1:1	$CHCl_3$: MeCN (1:1)	UV–vis	$1.92 (\pm 0.05) \times 10^4$ M^{-1}	Li:Na 33:1; Li:K 188;1	11
UC-3	$LiClO_4$	1:2	$CHCl_3$: MeCN (5:1)	UV–vis	$4.92 (\pm 1.16) \times 10^5$ M^{-2}	nr.	12
UC-3	$LiClO_4$	1:2	MeCN	FL	3.0×10^7 M^{-2}	Qual. 850 eqiv. Na will respond	12
UC-4	$LiClO_4$	1:1	MeCN	FL	2.8×10^3 M^{-1}	Nr.	12
H2	LiCl	1:1	MeCN	NMR	$2.01 (\pm 0.1) \times 10^5$ M^{-1}	(Li:Na:K:Mg:Ca): 1.0 : 0.61 : 0.10 : 1.11 : 0.51	13
H1	LiCl	1:1	MeCN	NMR	$5.55 (\pm 0.35) \times 10^2$ M^{-1}	(Li:Na:K:Mg:Ca): 1.0 : 0.59 : 0.10 : 1.10 : 0.50	13
$(C_6H_6)Ru_2$	LiOAc	1:1	H_2O (phosphate buffer, pH 7.0)	NMR	$2.3 (\pm 0.4) \times 10^3$ M^{-1}	Li > Na 10000:1	14
(*p*-$MeC_6H_4iPr)Ru_3$	LiOAc	1:1	D_2O	NMR	$8.4 (\pm 0.6) \times 10^2$ M^{-1}	Li > Na 10000:1	14
macrocycle; anthraquinone	$LiClO_4$	1:2	MeCN	UV–vis	$K_1 = 2.17 \times 10^2$ M^{-1} $K_2 = 1.24 \times 10^4$ M^{-1}	qualitative analysis No binding	15
DB14C4	$LiClO_4$	1:1	$CDCl_3$:CD_3CN (1:1)	NMR	2.36×10^3 M^{-1}	Li:Na 10.35:1	16
DB15C5	$LiClO_4$	1:1	$CDCl_3$:CD_3CN (1:1)	NMR	6.1×10^2 M^{-1}	Li:Na 1.62:1	
Aza crown ether	$LiClO_4$	1:1	MeCN	NMR	$K = 1.27 \times 10^2$ M^{-1}	quali only. Na was interacting with Li	17
Chiral diaza-9-crown-3 derivative	$LiClO_4$	1:1	MeCN	FL	$K = 2.5 \times 10^5$ M^{-1}	Li (all other cations evaluated insignificant change), several salts were used, but non-conclusive.	18
Thiacalix[4]crown	$LiClO_4$	1:1	Dry THF	FL	7.8×10^4 M^{-1}	Hg > Li	19
Benzo-9-crown-3 polymer	Li_2S	effective sandwich complex	H_2O/DCM	Liquid-liquid extraction (UV–vis)	$K_{ex} = 7.6 \times 10^6$	$Li^+ > Na^+, K^+$	20
Calix[4]Pyrroles	LiCl	1:1	THF-d_8/D_2O (9:1, v/v)	NMR	$(8.3 \pm 0.3) \times 10^2$ M^{-1}	in liquid-liquid extraction Na > Li (Li:Na 1:14.5) in Solid-liquid extraction Li > Na	21
**tBuBorph**	**LiTDDS**	**1:1**	**1,4-dioxane**	**UV-vis**	**$1.8 \pm 0.4 \times 10^6$ M^{-1}**	**-**	**This Work**
**STBorph**	**LiOTf**	**1:1**	**CPME**	**UV–vis**	**$3.9 \pm 2.0 \times 10^5$ M^{-1}**	**Li:Na > 3000:1 Li-selective extraction from mixed dry salts**	

Optimization of borphyrin formation

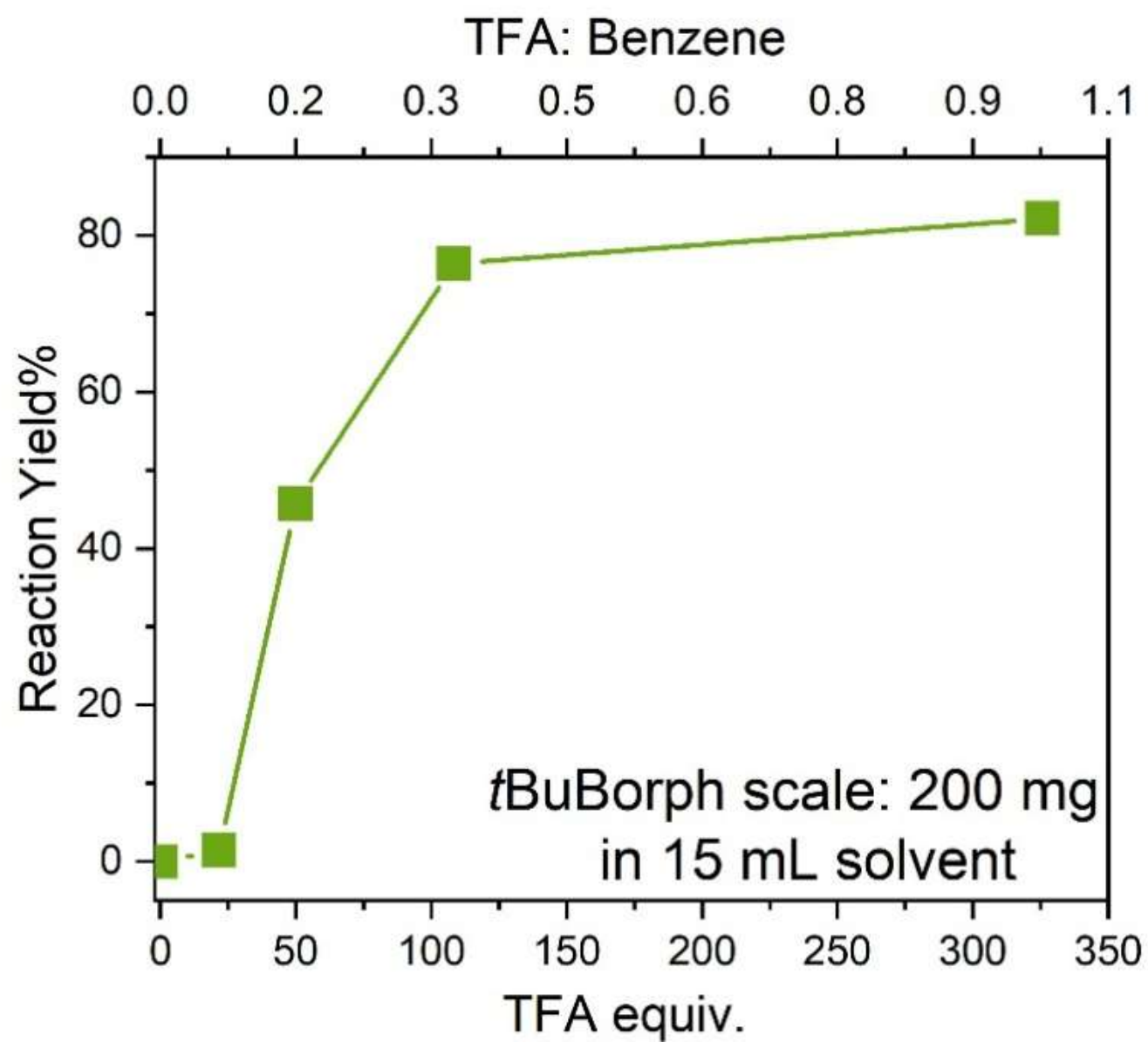

Fig. S1. Optimizing the role of TFA concentration in **tBuBorph** formation. Yields were determined by filtering the solids from the reaction mixture and washing with excess MeCN. The solvent volume was kept constant at 15 mL to maintain a consistent concentration of **tBuBorph** (200 mg / 15 mL) throughout the experiments.

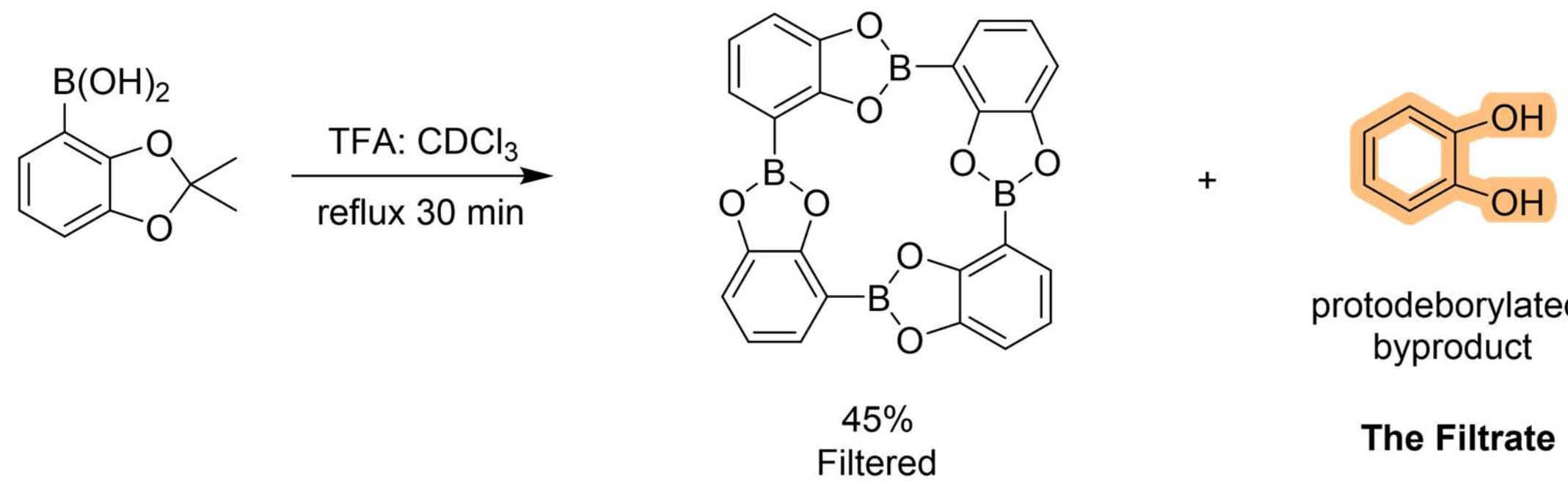

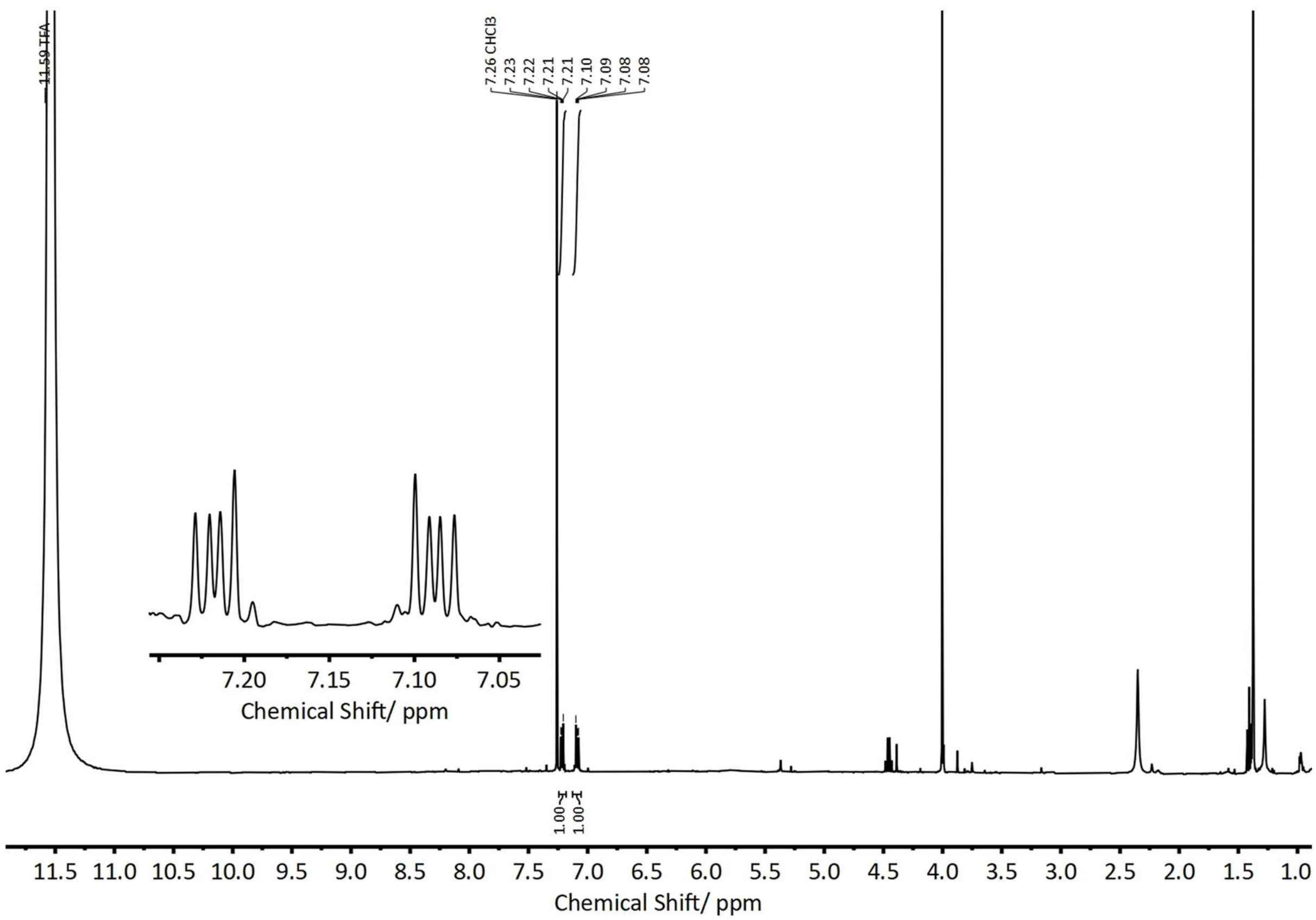

Fig. S2. Scheme of the reaction of unprotected (2,2-dimethyl-1,3-benzodioxol-4-yl)- boronic acid (**AceBOH**; top) and ^{1}H NMR spectrum ($CDCl_3$, 400 MHz) of the reaction filtrate residue after filtration of **HBorph** (45% isolated). The reaction condition was identical to that of **HBorph** as described in the synthesis section above. The results suggested that the major side-reaction (~55%) is the protodeborylation (produces catechol).

IR Spectra of borphyrins and AceBpin Precursors

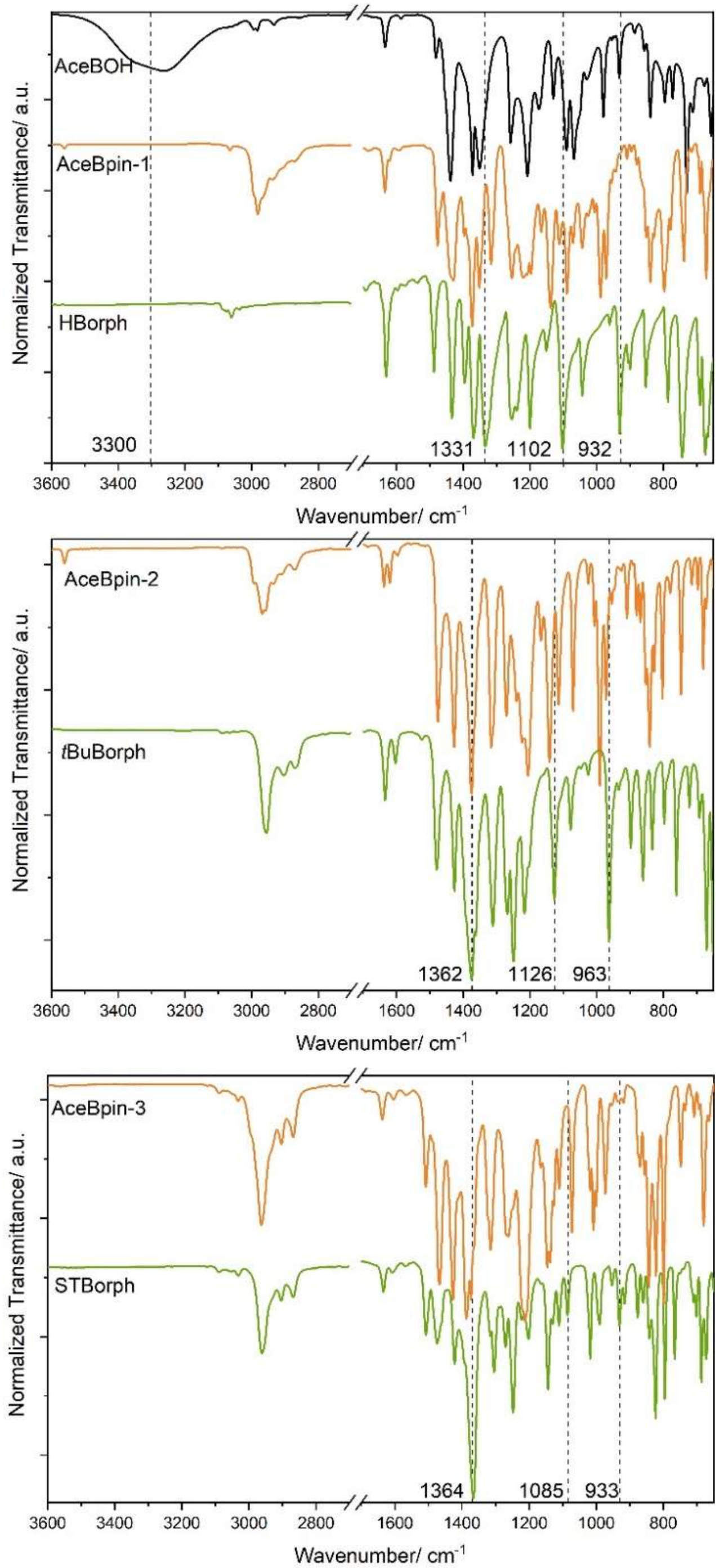

Fig. S3. IR spectra of the borphyrins and comparison with their corresponding **AceBpin** precursors and **AceBOH** precursor for **HBorph** (black).

Thermogravimetric Analysis

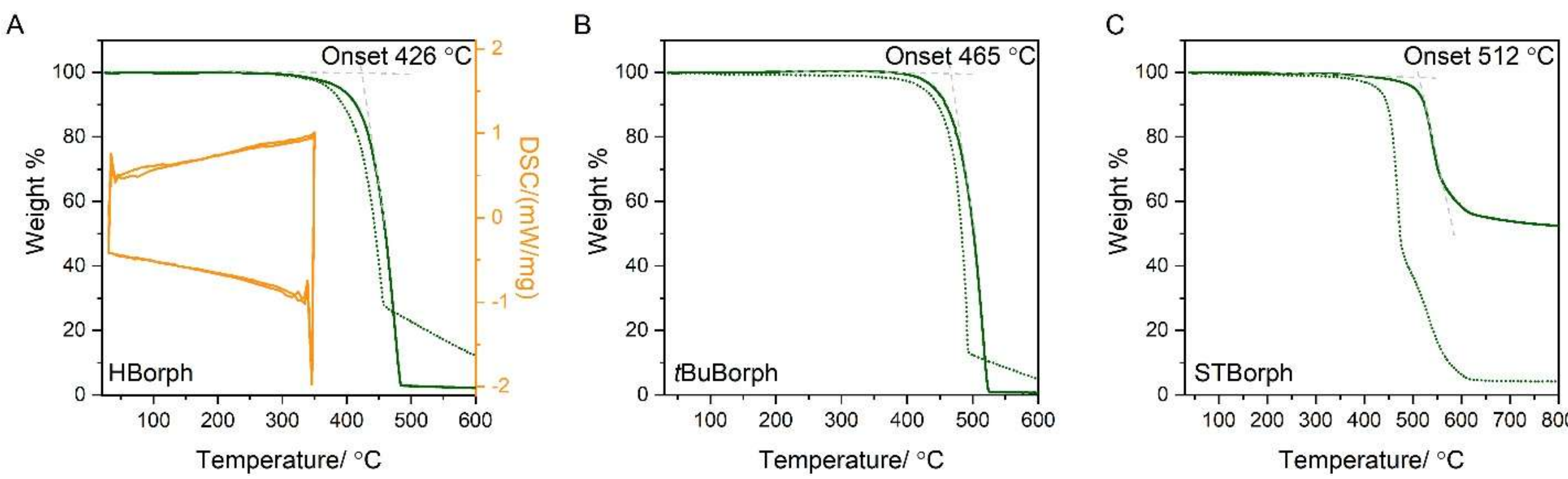

Fig. S4. TGA analysis (green) of (**A**) **HBorph** (**B**) **tBuBorph** and (**C**) **STBorph** under nitrogen (solid line) and in air flow (dotted). The inset of (**A**) shows the DSC analysis of **HBorph** (orange, right axis). Samples were analyzed at a heating rate of 10 °C min^{-1}.

Computational details and UV–vis extinction coefficients

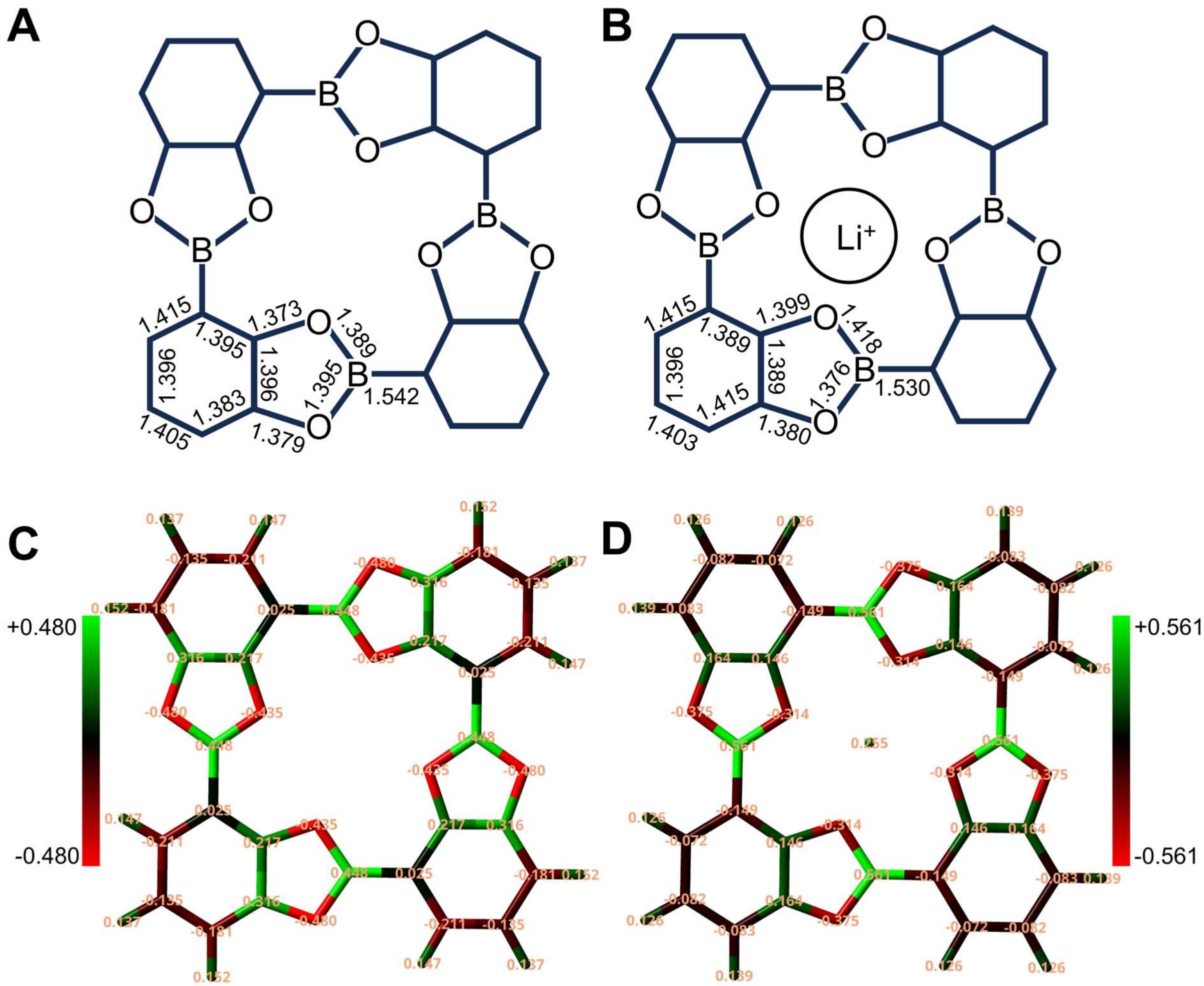

Fig. S5. Wiberg bond index analysis (the bond lengths are written on top of the corresponding bond) of the optimized structure simulated with density functional theory (DFT; B3LYP 6-31-G(d)) for (**A**) the **HBorph** and (**B**) [Li**HBorph**]$^{+}$ complex. Mulliken charge distribution analysis (the charge values are written on each atom) of (**C**) **HBorph** and (**D**) [Li**HBorph**]$^{+}$ extracted from the same DFT calculations.

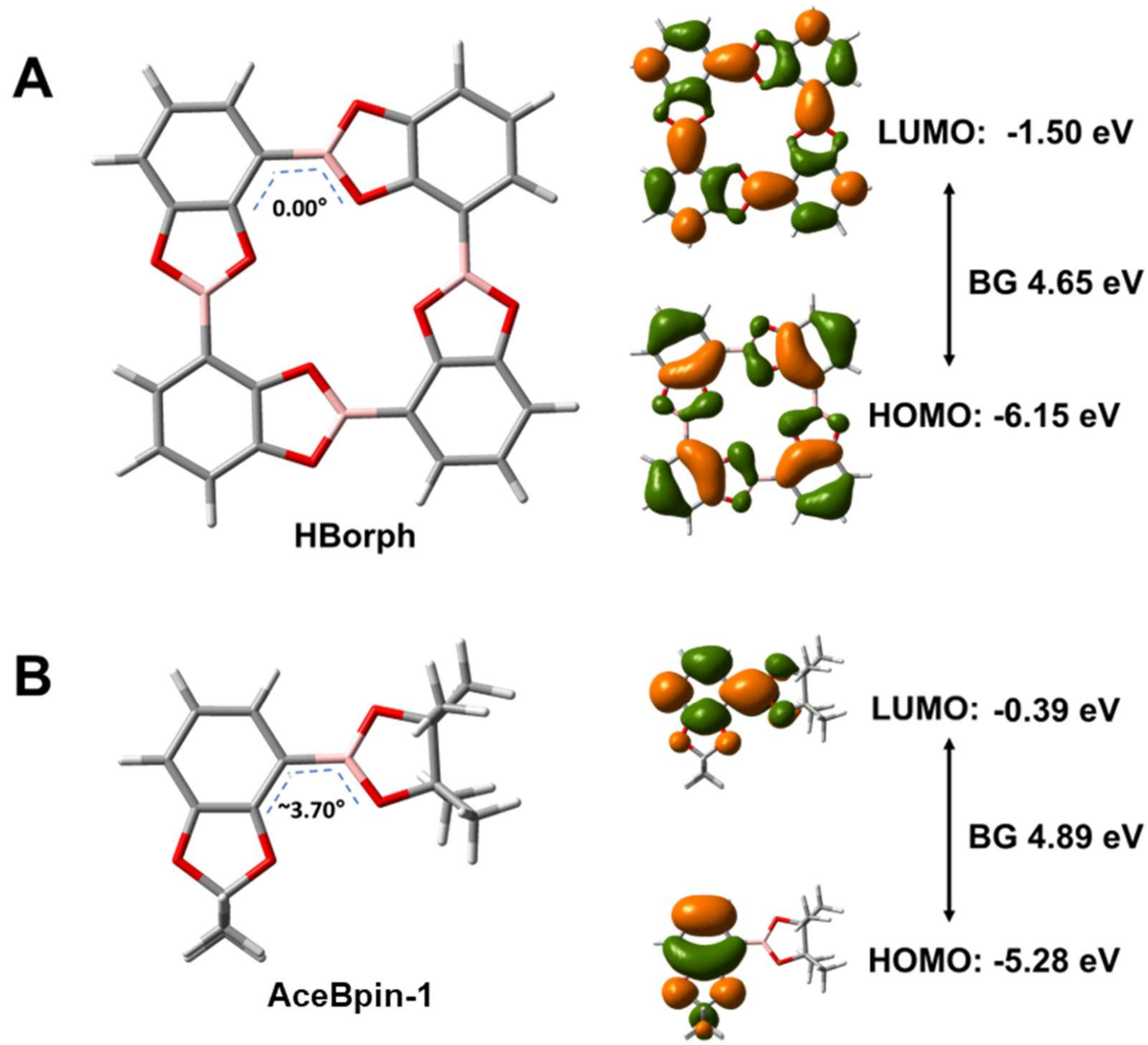

Fig. S6. The optimized geometry (left) and frontier molecular orbitals(right) of (**A**) **HBorph** and (**B**) **AceBpin-1** obtained from gas phase density functional theory (DFT; B3LYP 6-31G(d)). The inset numbers in optimized geometries are the dihedral angle between C_{Ar}— C_{Ar}—B—O, showing the planarity of borphyrin structure as compared to slightly twisted boronic ester in **AceBpin-1**.

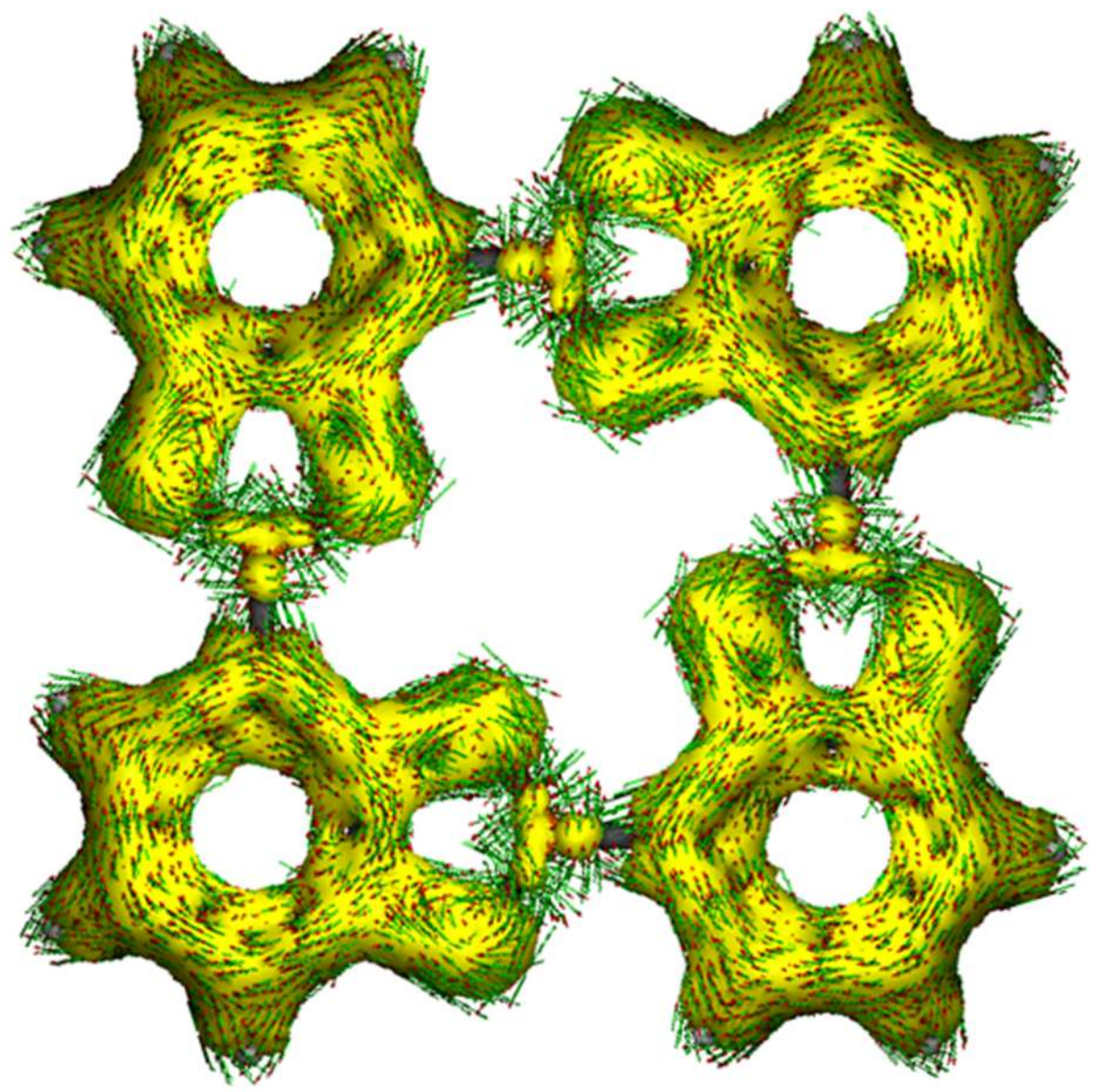

Fig. S7. Anisotropy of the induced current density (AICD) map of the borphyrin **HBorph** showing the node at C-B-O center hampering the π-conjugation of the borphyrin core.

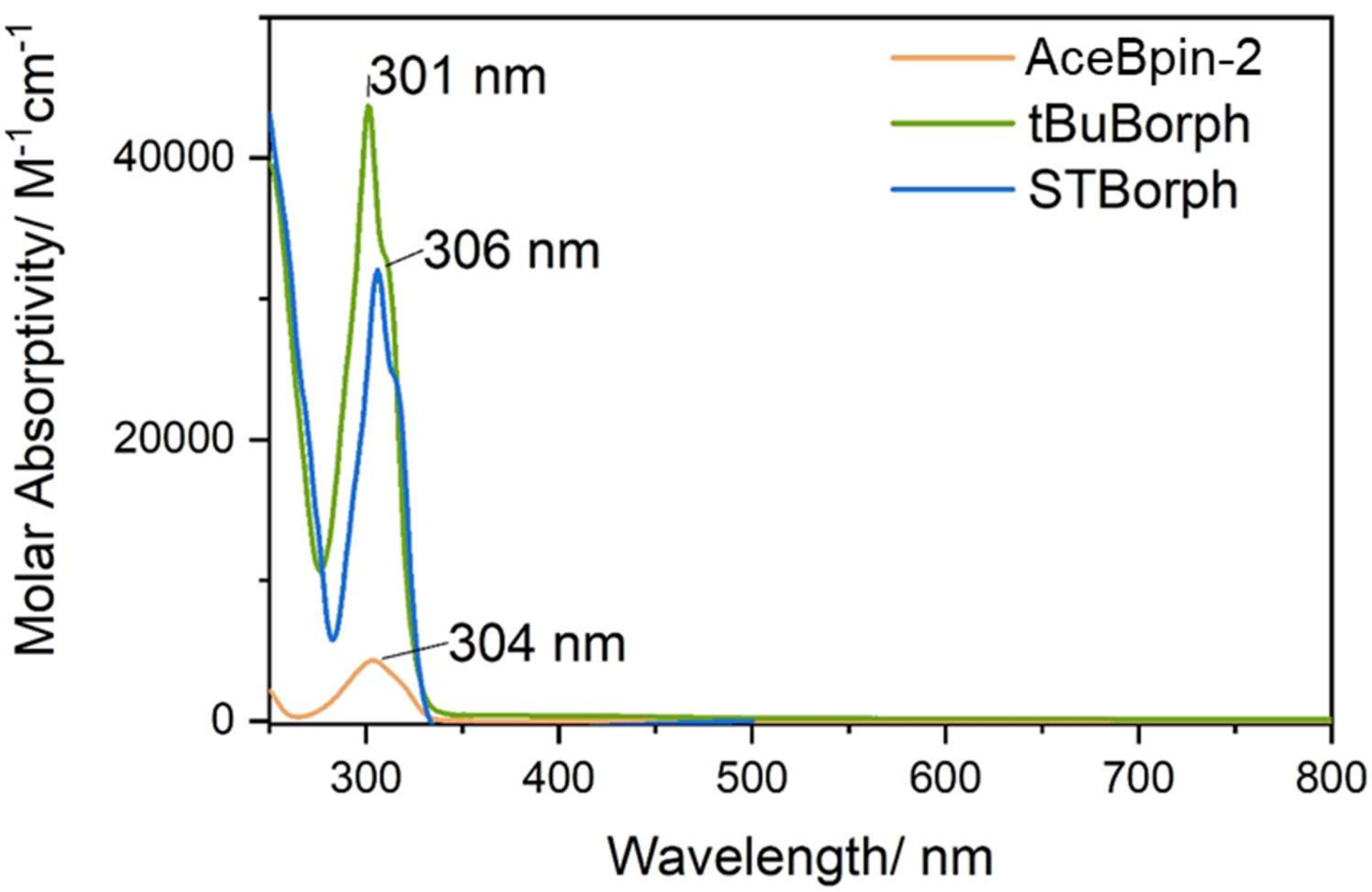

Fig. S8. Extinction coefficient of **AceBpin-2**, **tBuBorph** and **STBorph** obtained in dioxane at room temperature in a 1 cm quartz cell.

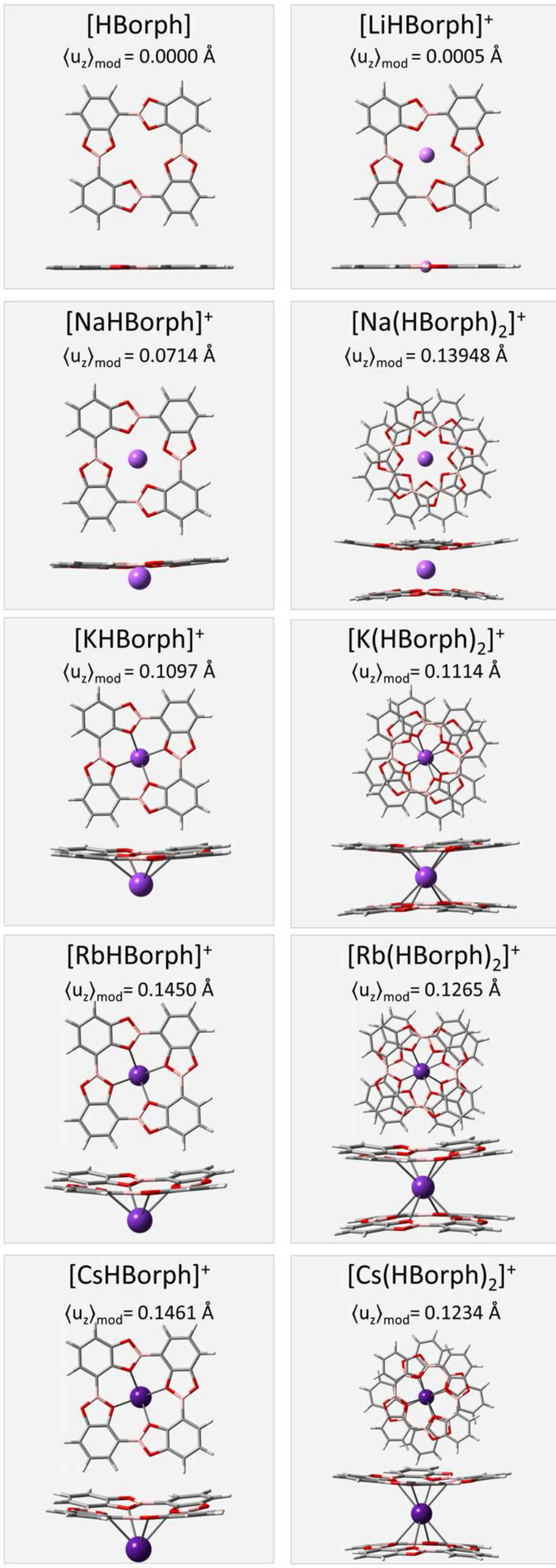

Fig. S9. DFT-optimized geometries (B3LYP 6-31G(d)) of borphyrin complexes with different alkali metals in either ML^+ or ML_2^+ configurations. The mean out-of-plane displacement ($\langle u_z \rangle_{mod}$) was calculated from these optimized geometries by fitting a least-squares mean plane (using Mercury software) to the borphyrin scaffold (all atoms except H and the metal center) and averaging the displacement of all scaffold atoms from this plane. For ML_2^+ complexes, each

borphyrin unit was analyzed separately, and the reported value represents the average of the individual $\langle u_z \rangle_{mod}$ values for each scaffold.

Table S2. Summary of the alkali metal + **HBorph** complexes' calculations. The calculations are done with B3LYP method in gaussian G016.revC1. The coordinates of the optimized structures are attached as supplementary data.

Complex	Basis set	Energy (Hartree)	# Imaginary freq.
[Hborph]	6-31G(d)	-1623.43440	0
$[LiHBorph]^+$	6-31G(d)	-1630.87500	0
$[NaHBoph]^+$	6-31G(d)	-1785.62630	0
$[Na(HBorph)_2]^+$	6-31G(d)	-3409.10420	0
$[KHBorph]^+$	6-31G(d)	-2223.22900	0
$[K(HBorph)_2]^+$	6-31G(d)	-3846.71220	0
$[RbHBorph]^+$	LANL2DZ	-1646.94370	0
$[Rb(HBorph)_2]^+$	LANL2DZ	-3270.16280	2
$[CsHBorph]^+$	LANL2DZ	-1642.95680	0
$[Cs(HBorph)_2]^+$	LANL2DZ	-3266.16780	0

UV–vis titration

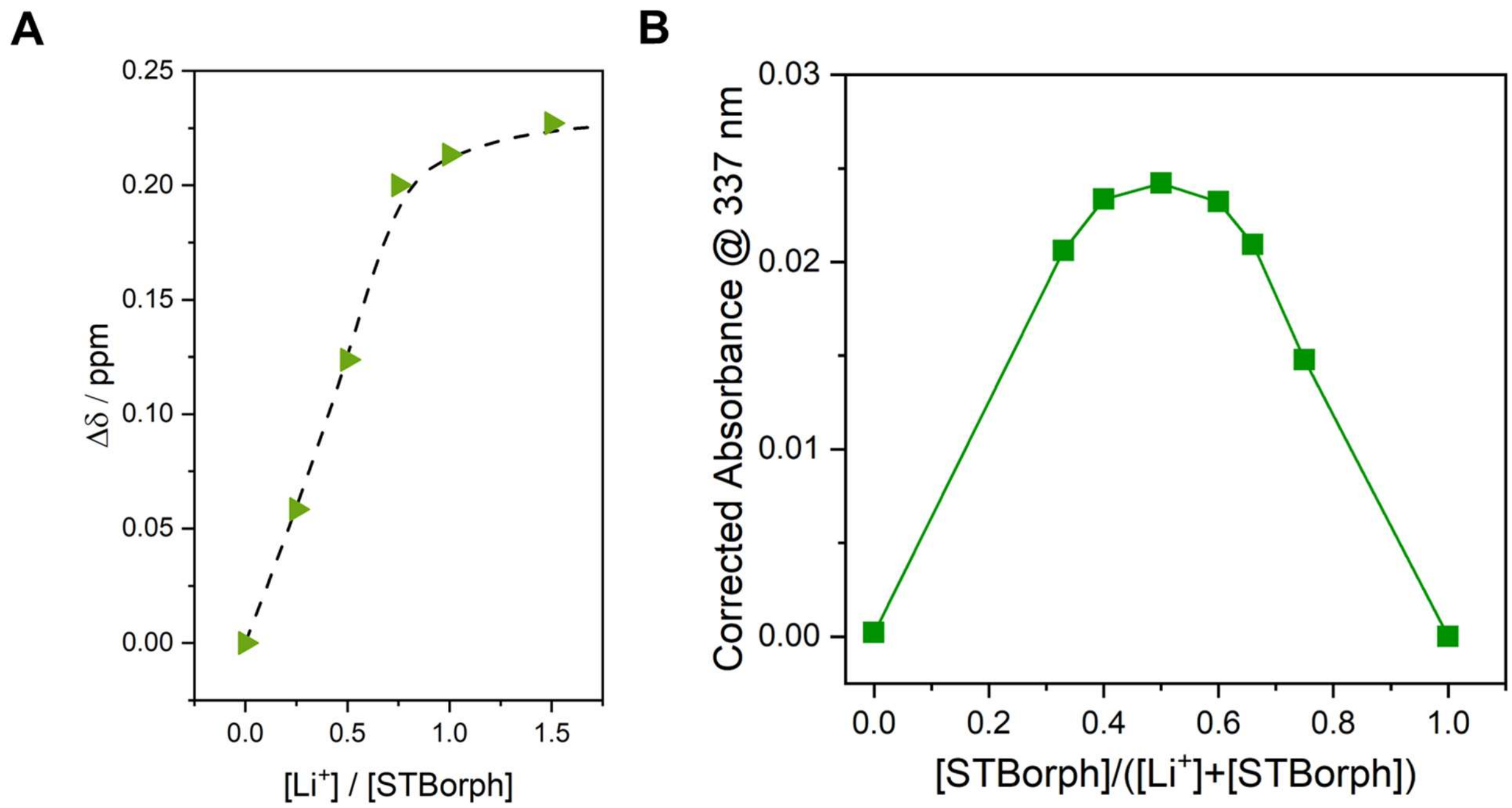

Fig. S10. Determination of stoichiometry of Li^+ : **STBorph**. (**A**) ^1H NMR titration of **STBorph** (obtained from the NMR spectra in Figure 2C) and (**B**) Job's plot of the **STBorph** and $LiBF_4$, obtained via UV–vis spectroscopy in THF, both suggesting the 1:1 complexation of the borphyrin with lithium.

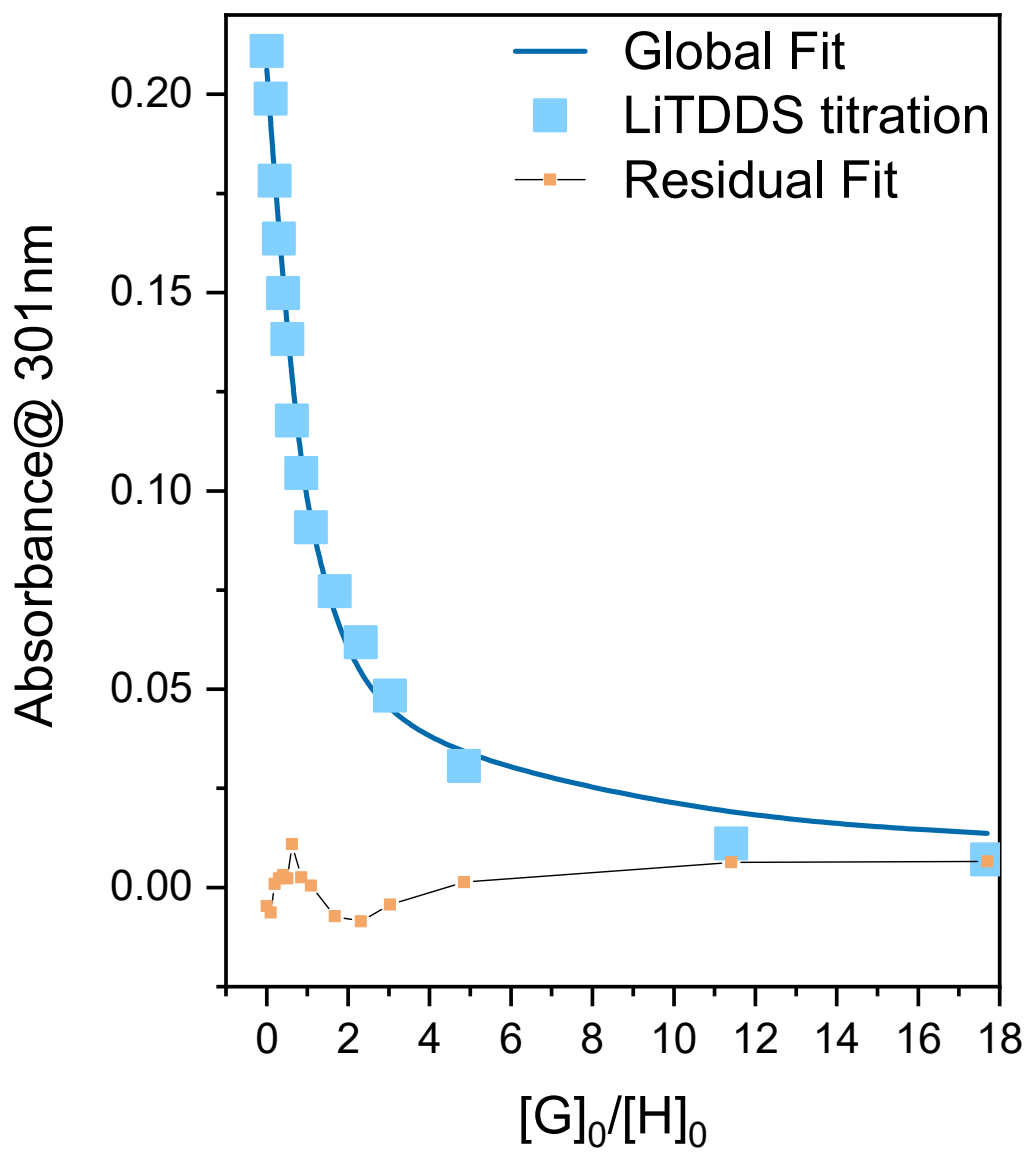

Fig. S11. Global fitting data for the UV–vis titration of **tBuBorph** ([**tBuBorph**] = 2 μM) in 1,4-dioxane with LiTDDS added from a stock solution in 1,4-dioxane ([LiTDDS] = 0.1 mM). The titration data were fitted using a 1:1 binding model. See Fig. 2D of the main manuscript for the titration curve.

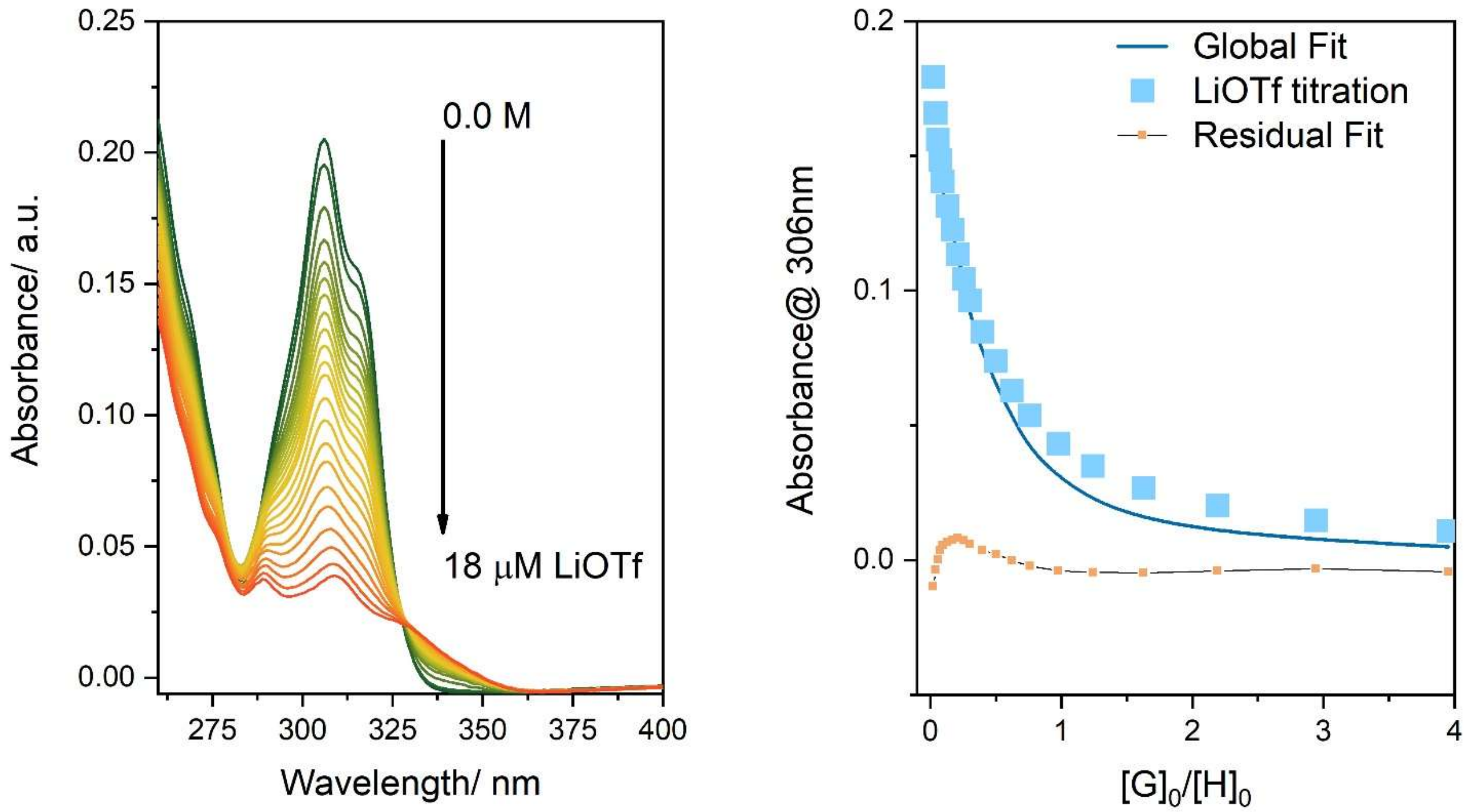

Fig. S12. UV–vis titration of **STBorph** ([**STBorph**] = 2 μM) in CPME with LiOTf added from a CPME stock solution ([LiOTf] = 0.1 mM), together with the corresponding global fitting data.

Three independent titrations gave an average association constant of $K_a = (3.9 \pm 2.0) \times 10^5\ M^{-1}$ for the **STBorph**⊃Li^+ complex using a 1:1 binding model.

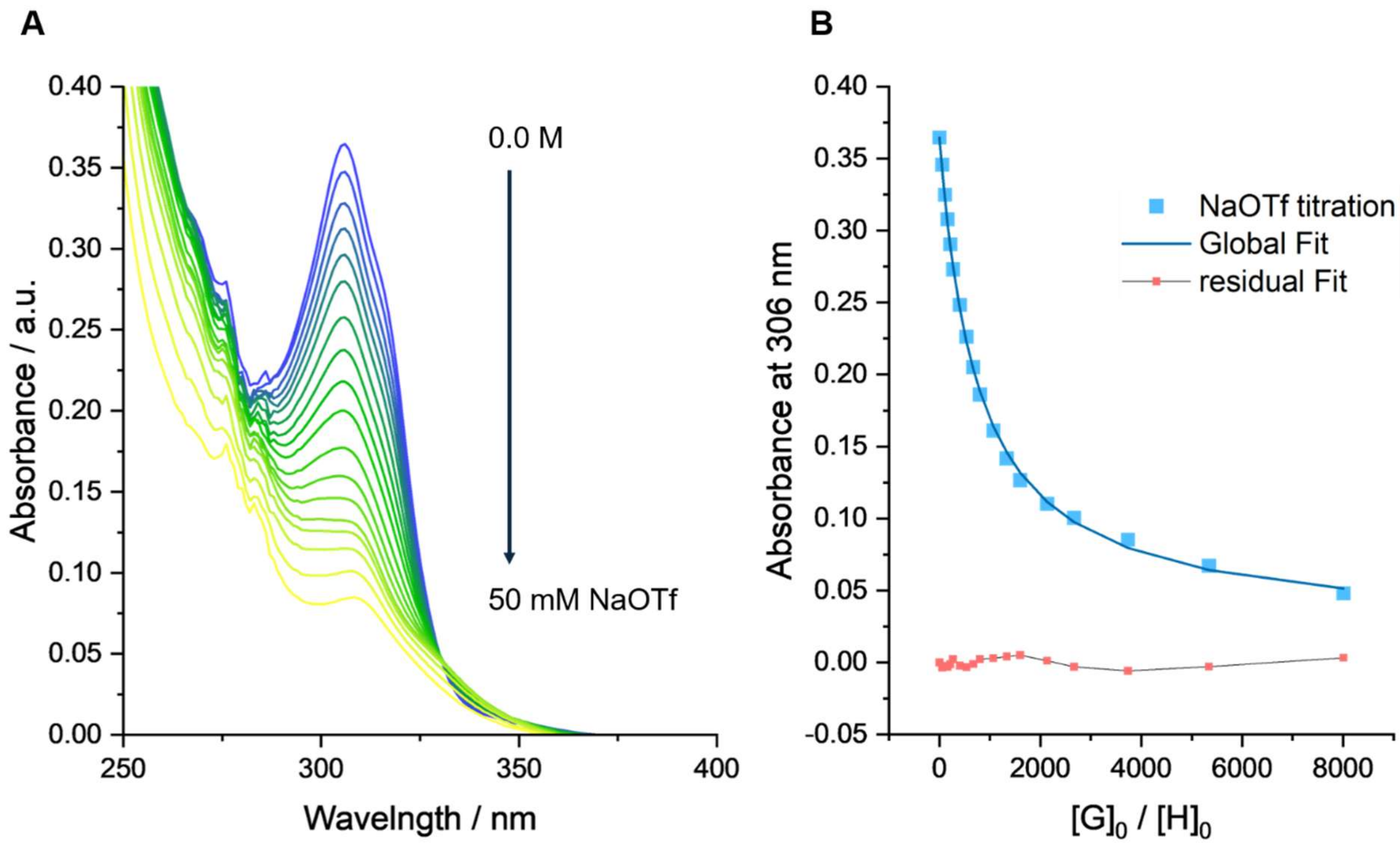

Fig S13. UV–vis titration of **STBorph** with NaOTf. (**A**) UV–vis spectra of **STBorph** ([**STBorph**] = 10 μM) in THF upon addition of NaOTf from a concentrated THF stock solution at 25 ˚C. The final NaOTf concentration was 50.2 mM. (**B**) Representative global fitting data for the NaOTf titration, monitored at 306 nm, using a 1:1 binding model. The residuals are shown below the fit. Three independent titrations gave an apparent association constant of $K_a = (0.9 \pm 0.3) \times 10^2\ M^{-1}$ for Na^+ binding. A higher-order 2:1 binding model was also evaluated for the three independent Na^+ titrations. However, the apparent fitted constants returned by BindFit were not consistent across the datasets: K_{11} = ~90 M^{-1} (err. ~6%) and K_{21} = ~2.9 × 10^4 M^{-1} (err. ~30%) for titration 1; K_{11} = ~231 M^{-1} (err. ~12%) and K_{21} = ~7.5 × 10^{-7} M^{-1} (err. ~23%) for titration 2; and K_{11} = ~9.4 × 10^{14} M^{-1} (err. >10^8%) and K_{21} = ~4.9 × 10^{18} M^{-1} (err. >10^8%) for titration 3. These values

indicate that the 2:1 model is poorly constrained and physically unrealistic under these conditions. Therefore, the Na^+ response was reported using an apparent 1:1 binding model. It is noteworthy that at such high concentrations, even scarce Li^+ impurities (at least 10 ppm in metal assay Na salts), and viscosity changes may contribute to the observed response.

UV–vis solid extraction procedure

A 5 g sea-salt–mimicking mixture was prepared containing 0.2 : 10500 : 380 : 1250 : 400 of Li^+ : Na^+ : K^+ : Mg^{2+} : Ca^{2+} ions, respectively. A control salt mixture (5 g) was prepared using the same ionic ratios but without lithium. Approximately 10 mL of deionized water was added to each mixture to ensure homogeneous mixing, followed by drying in an oven at 160 °C overnight. Separately, approximately 3.0 mg of **STBorph** was dissolved in 5 mL of cyclopentyl methyl ether (CPME). The resulting solution was diluted tenfold by transferring a 1 mL aliquot and diluting to a final volume of 10 mL with CPME. UV–vis absorption measurements were carried out using an Agilent Cary Series NIR spectrophotometer operated in single-beam (“Single Front”) mode with a 1 cm path-length quartz cuvette. A baseline correction was performed using CPME as the blank. Subsequently, 3 mL of the **STBorph** solution was transferred to the cuvette to record the initial UV–vis spectrum. The solution was then returned to the original scintillation vial, and approximately 110 mg of the lithium-free sea-salt mixture was added. The vial was shaken for 30 s, after which 3 mL of the solution was transferred to the cuvette using a syringe equipped with a 0.2 µm PTFE syringe filter, and the UV–vis spectrum was recorded. This procedure was repeated with incremental additions of 220 mg, 550 mg, and 1.10 g of the salt mixture. Following completion of the control experiments, the entire procedure was repeated under identical conditions using the lithium-containing sea-salt mixture.

UV–vis binding studies with different lithium salts

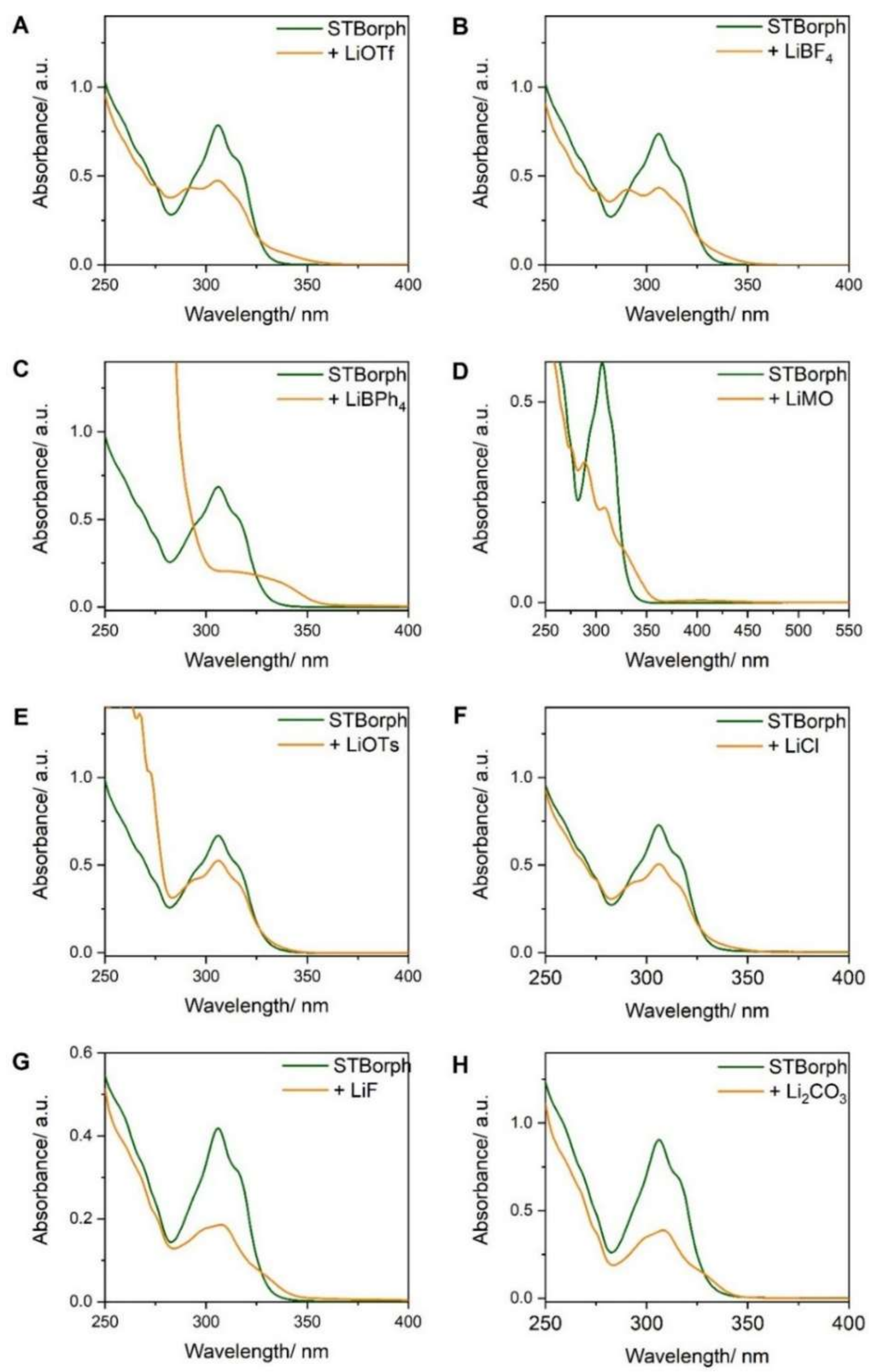

Fig. S14. Lithium complexation to various lithium salts including ether soluble (**A**-**E**) and ether insoluble salts (F-H). In a typical experiment, a ~3 μM solution of **STBorph** in cyclopentyl methyl ether (CPME) solvent was freshly prepared, 3 mL was transferred to the quartz cuvette to record

the initial UV–vis spectrum. The solution was then added into a clean scintillation vial, and approximately 0.1 mg of lithium salts were added. The vial was shaken for 30 s (~5 min in case of **F**-**H**), after which the solution was transferred back to the cuvette and the UV–vis spectrum was recorded again. (Note: part **D** is shown with a different scale as the counter ion absorbs in the visible range)

UV–vis evaluation of lithium binding in the presence of excess Na^+

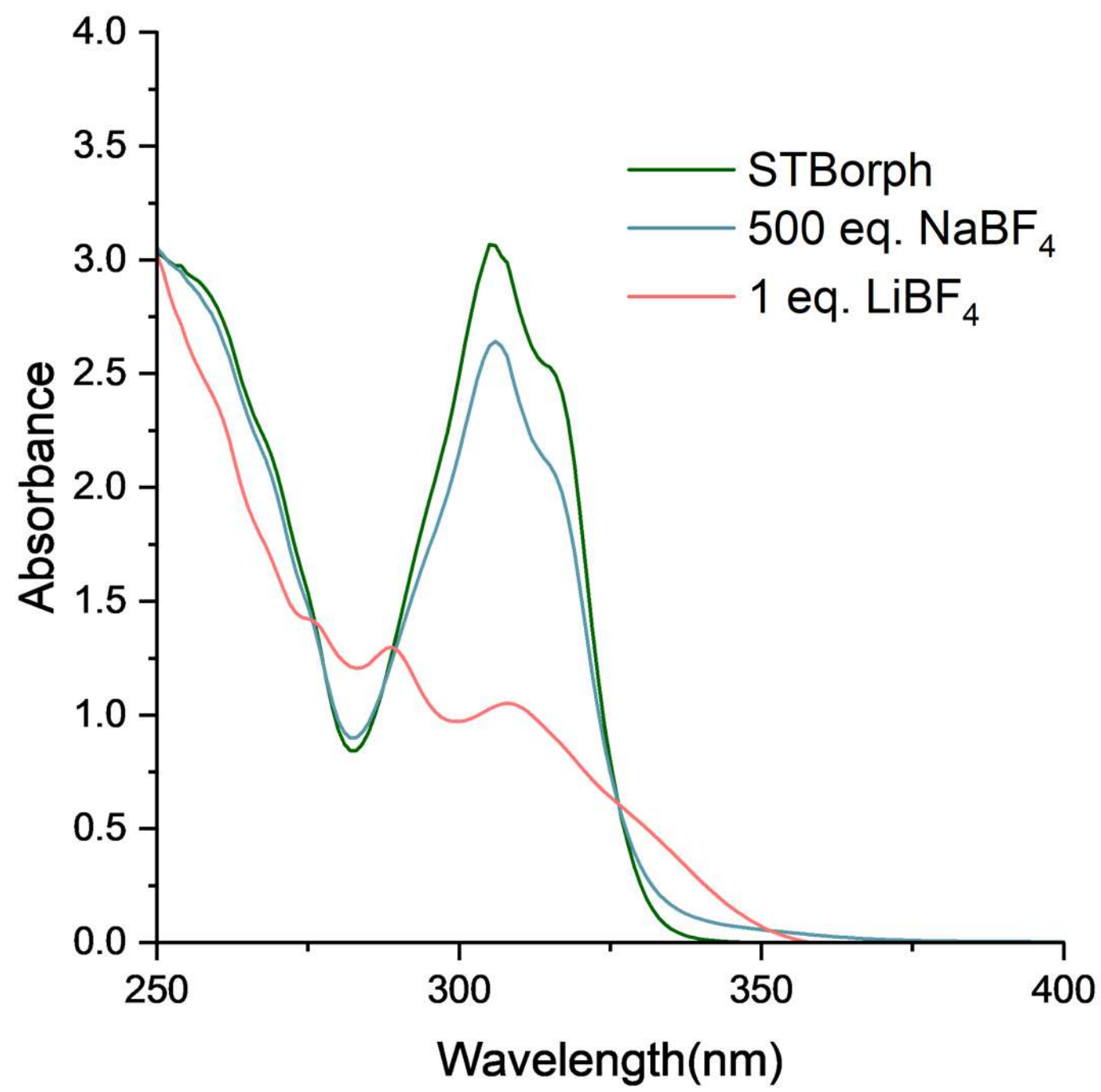

Fig. S15. UV–vis spectrum of **STBorph** ([**STBorph**] = 0.100 mM) in CPME after exposure to excess solid $NaBF_4$ (nominally 500 equiv. relative to **STBorph**; not fully dissolved under these conditions), followed by addition of $LiBF_4$ (1 equiv. relative to **STBorph**). Only a small spectral change was observed after exposure to $NaBF_4$, whereas subsequent $LiBF_4$ addition produced a pronounced spectral response consistent with preferential Li^+ binding. According to the certificate of analysis, the $NaBF_4$ used in this experiment (trace-metal basis 99%, Sigma-Aldrich, batch

MKCW8418) contained trace Li^+ impurities (2.3 ppm), which may account for the small spectral change observed before intentional $LiBF_4$ addition.

Borph-Li complex NMR characterization

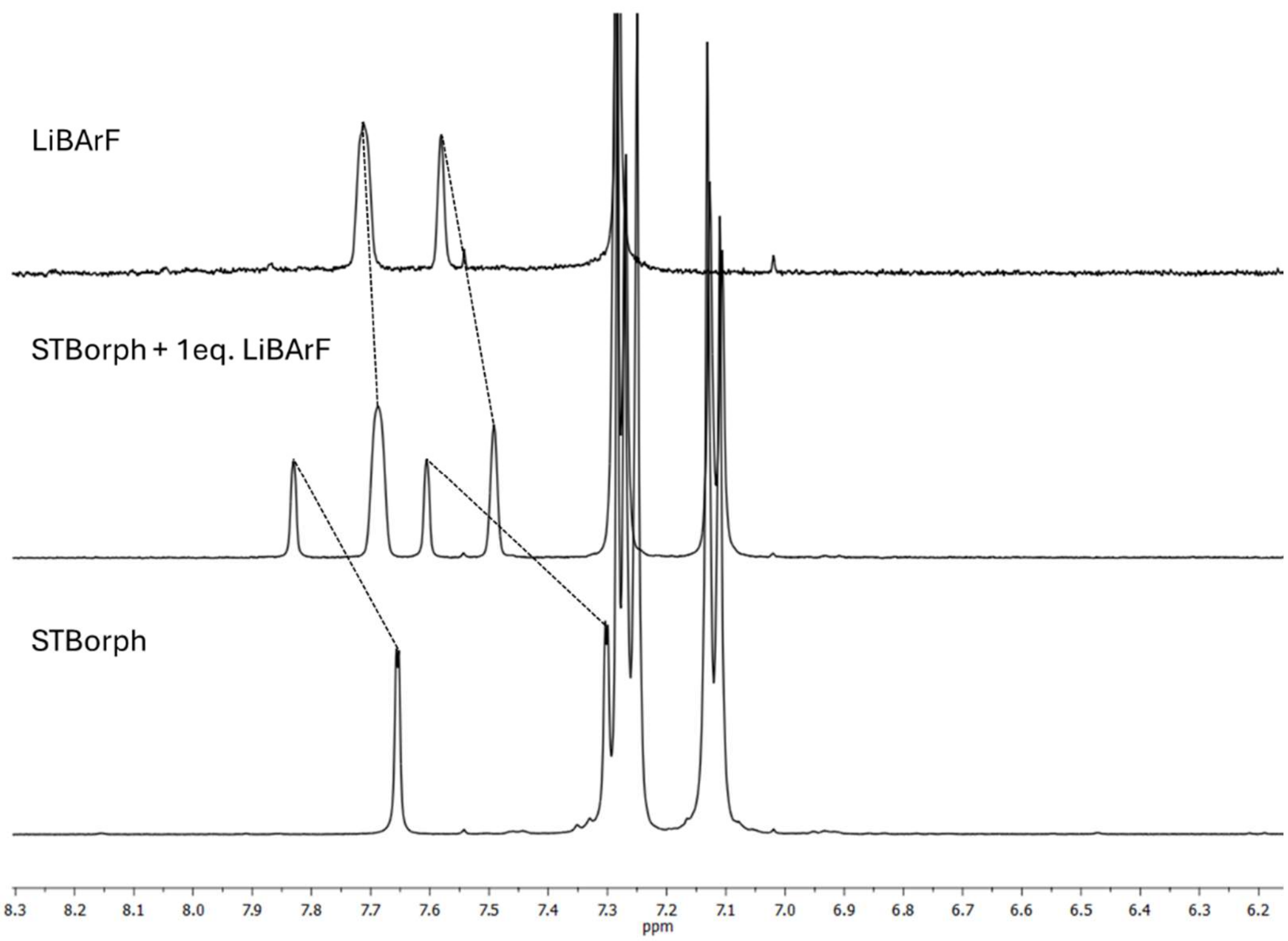

Fig. S16. Partial ^{1}H NMR (400 MHz, THF-d_8) spectra (aromatic region) of **STBorph** ([**STBorph**] = 1.0 mM; bottom), **STBorph** with 1 equiv of LiBArF (middle), and LiBArF alone (top). LiBArF was added to enhance the solubility of the lithium salt. Upon Li^{+} addition, pronounced chemical shift changes are observed for **STBorph**, indicating strong host-guest interactions in solution.

Crystallization trials of Li^{+}-bound borphyrin complexes

Crystallization of Li^{+}-bound borphyrin complexes was pursued over approximately two years using a range of lithium salts, borphyrin derivatives, and solvent systems. A focused 96-sample screen was also performed under rigorously dry and inert-atmosphere conditions using

selected promising conditions. No single crystals of a borphyrin-containing Li^+ complex suitable for single-crystal X-ray diffraction were obtained.

Table S3. Summary of crystallization trials for Li^+-bound borphyrin complexes, including a focused 96-sample screen performed under dry and inert-atmosphere conditions.

#	Compound	Amount (mg)	Amount (μmol)	Good Solvent	Poor Solvent	Li Salt	Li (equiv)	Li (μmol)
**1-1**	**tBuBorph**	0.5	0.718	$CHCl_3$	—	$LiNTf_2$	3	2.154
**1-2**	**tBuBorph**	0.5	0.718	$CHCl_3$	—	LiBArF	3	2.154
**1-3**	**tBuBorph**	0.5	0.718	$CHCl_3$	—	LiOTf	3	2.154
**1-4**	**tBuBorph**	0.5	0.718	$CHCl_3$	—	LiI	3	2.154
**1-5**	**tBuBorph**	0.5	0.718	THF	—	$LiNTf_2$	3	2.154
**1-6**	**tBuBorph**	0.5	0.718	THF	—	LiBArF	3	2.154
**1-7**	**tBuBorph**	0.5	0.718	THF	—	LiOTf	3	2.154
**1-8**	**tBuBorph**	0.5	0.718	THF	—	LiI	3	2.154
**1-9**	**tBuBorph**	0.5	0.718	$CHCl_3$	Hexanes	$LiNTf_2$	3	2.154
**1-10**	**tBuBorph**	0.5	0.718	$CHCl_3$	Hexanes	LiBArF	3	2.154
**1-11**	**tBuBorph**	0.5	0.718	$CHCl_3$	Hexanes	LiOTf	3	2.154
**1-12**	**tBuBorph**	0.5	0.718	$CHCl_3$	Hexanes	LiI	3	2.154
**1-13**	**tBuBorph**	0.5	0.718	$CHCl_3$	MeCN	$LiNTf_2$	3	2.154
**1-14**	**tBuBorph**	0.5	0.718	$CHCl_3$	MeCN	LiBArF	3	2.154
**1-15**	**tBuBorph**	0.5	0.718	$CHCl_3$	MeCN	LiOTf	3	2.154
**1-16**	**tBuBorph**	0.5	0.718	$CHCl_3$	MeCN	LiI	3	2.154
**1-17**	**tBuBorph**	0.5	0.718	THF	Hexanes	$LiNTf_2$	3	2.154
**1-18**	**tBuBorph**	0.5	0.718	THF	Hexanes	LiBArF	3	2.154
**1-19**	**tBuBorph**	0.5	0.718	THF	Hexanes	LiOTf	3	2.154
**1-20**	**tBuBorph**	0.5	0.718	THF	Hexanes	LiI	3	2.154
**1-21**	**tBuBorph**	0.5	0.718	THF	MeCN	$LiNTf_2$	3	2.154
**1-22**	**tBuBorph**	0.5	0.718	THF	MeCN	LiBArF	3	2.154
**1-23**	**tBuBorph**	0.5	0.718	THF	MeCN	LiOTf	3	2.154
**1-24**	**tBuBorph**	0.5	0.718	THF	MeCN	LiI	3	2.154
**1-25**	**STBorph**	1	0.473	$CHCl_3$	—	$LiNTf_2$	3	1.419
**1-26**	**STBorph**	1	0.473	$CHCl_3$	—	LiBArF	3	1.419
**1-27**	**STBorph**	1	0.473	$CHCl_3$	—	LiOTf	3	1.419
**1-28**	**STBorph**	1	0.473	$CHCl_3$	—	LiI	3	1.419
**1-29**	**STBorph**	1	0.473	THF	—	$LiNTf_2$	3	1.419
**1-30**	**STBorph**	1	0.473	THF	—	LiBArF	3	1.419
**1-31**	**STBorph**	1	0.473	THF	—	LiOTf	3	1.419
**1-32**	**STBorph**	1	0.473	THF	—	LiI	3	1.419
**1-33**	**STBorph**	1	0.473	$CHCl_3$	Hexanes	$LiNTf_2$	3	1.419
**1-34**	**STBorph**	1	0.473	$CHCl_3$	Hexanes	LiBArF	3	1.419
**1-35**	**STBorph**	1	0.473	$CHCl_3$	Hexanes	LiOTf	3	1.419
**1-36**	**STBorph**	1	0.473	$CHCl_3$	Hexanes	LiI	3	1.419
**1-37**	**STBorph**	1	0.473	$CHCl_3$	MeCN	$LiNTf_2$	3	1.419
**1-38**	**STBorph**	1	0.473	$CHCl_3$	MeCN	LiBArF	3	1.419
**1-39**	**STBorph**	1	0.473	$CHCl_3$	MeCN	LiOTf	3	1.419
**1-40**	**STBorph**	1	0.473	$CHCl_3$	MeCN	LiI	3	1.419
**1-41**	**STBorph**	1	0.473	THF	Hexanes	$LiNTf_2$	3	1.419
**1-42**	**STBorph**	1	0.473	THF	Hexanes	LiBArF	3	1.419

**1-43**	**STBorph**	1	0.473	THF	Hexanes	LiOTf	3	1.419
**1-44**	**STBorph**	1	0.473	THF	Hexanes	LiI	3	1.419
**1-45**	**STBorph**	1	0.473	THF	MeCN	$LiNTf_2$	3	1.419
**1-46**	**STBorph**	1	0.473	THF	MeCN	LiBArF	3	1.419
**1-47**	**STBorph**	1	0.473	THF	MeCN	LiOTf	3	1.419
**1-48**	**STBorph**	1	0.473	THF	MeCN	LiI	3	1.419
**2-1**	**STBorph**	1	0.473	Dioxane	—	$LiNTf_2$	3	1.419
**2-2**	**STBorph**	1	0.473	Dioxane	—	LiBArF	3	1.419
**2-3**	**STBorph**	1	0.473	Dioxane	—	LiOTf	3	1.419
**2-4**	**STBorph**	1	0.473	Dioxane	—	LiI	3	1.419
**2-5**	**STBorph**	1	0.473	1,2-DCE	—	$LiNTf_2$	3	1.419
**2-6**	**STBorph**	1	0.473	1,2-DCE	—	LiBArF	3	1.419
**2-7**	**STBorph**	1	0.473	1,2-DCE	—	LiOTf	3	1.419
**2-8**	**STBorph**	1	0.473	1,2-DCE	—	LiI	3	1.419
**2-9**	**STBorph**	1	0.473	Dioxane	Et_2O	$LiNTf_2$	3	1.419
**2-10**	**STBorph**	1	0.473	Dioxane	Et_2O	LiBArF	3	1.419
**2-11**	**STBorph**	1	0.473	Dioxane	Et_2O	LiOTf	3	1.419
**2-12**	**STBorph**	1	0.473	Dioxane	Et_2O	LiI	3	1.419
**2-13**	**STBorph**	1	0.473	Dioxane	Pentane	$LiNTf_2$	3	1.419
**2-14**	**STBorph**	1	0.473	Dioxane	Pentane	LiBArF	3	1.419
**2-15**	**STBorph**	1	0.473	Dioxane	Pentane	LiOTf	3	1.419
**2-16**	**STBorph**	1	0.473	Dioxane	Pentane	LiI	3	1.419
**2-17**	**STBorph**	1	0.473	1,2-DCE	Et_2O	$LiNTf_2$	3	1.419
**2-18**	**STBorph**	1	0.473	1,2-DCE	Et_2O	LiBArF	3	1.419
**2-19**	**STBorph**	1	0.473	1,2-DCE	Et_2O	LiOTf	3	1.419
**2-20**	**STBorph**	1	0.473	1,2-DCE	Et_2O	LiI	3	1.419
**2-21**	**STBorph**	1	0.473	1,2-DCE	Pentane	$LiNTf_2$	3	1.419
**2-22**	**STBorph**	1	0.473	1,2-DCE	Pentane	LiBArF	3	1.419
**2-23**	**STBorph**	1	0.473	1,2-DCE	Pentane	LiOTf	3	1.419
**2-24**	**STBorph**	1	0.473	1,2-DCE	Pentane	LiI	3	1.419
**2-25**	**tBuBorph**	0.5	0.718	Dioxane	—	$LiNTf_2$	3	2.154
**2-26**	**tBuBorph**	0.5	0.718	Dioxane	—	LiBArF	3	2.154
**2-27**	**tBuBorph**	0.5	0.718	Dioxane	—	LiOTf	3	2.154
**2-28**	**tBuBorph**	0.5	0.718	Dioxane	—	LiI	3	2.154
**2-29**	**tBuBorph**	0.5	0.718	1,2-DCE	—	$LiNTf_2$	3	2.154
**2-30**	**tBuBorph**	0.5	0.718	1,2-DCE	—	LiBArF	3	2.154
**2-31**	**tBuBorph**	0.5	0.718	1,2-DCE	—	LiOTf	3	2.154
**2-32**	**tBuBorph**	0.5	0.718	1,2-DCE	—	LiI	3	2.154
**2-33**	**tBuBorph**	0.5	0.718	Dioxane	Et_2O	$LiNTf_2$	3	2.154
**2-34**	**tBuBorph**	0.5	0.718	Dioxane	Et_2O	LiBArF	3	2.154
**2-35**	**tBuBorph**	0.5	0.718	Dioxane	Et_2O	LiOTf	3	2.154
**2-36**	**tBuBorph**	0.5	0.718	Dioxane	Et_2O	LiI	3	2.154
**2-37**	**tBuBorph**	0.5	0.718	Dioxane	Pentane	$LiNTf_2$	3	2.154
**2-38**	**tBuBorph**	0.5	0.718	Dioxane	Pentane	LiBArF	3	2.154
**2-39**	**tBuBorph**	0.5	0.718	Dioxane	Pentane	LiOTf	3	2.154
**2-40**	**tBuBorph**	0.5	0.718	Dioxane	Pentane	LiI	3	2.154
**2-41**	**tBuBorph**	0.5	0.718	1,2-DCE	Et_2O	$LiNTf_2$	3	2.154
**2-42**	**tBuBorph**	0.5	0.718	1,2-DCE	Et_2O	LiBArF	3	2.154
**2-43**	**tBuBorph**	0.5	0.718	1,2-DCE	Et_2O	LiOTf	3	2.154
**2-44**	**tBuBorph**	0.5	0.718	1,2-DCE	Et_2O	LiI	3	2.154
**2-45**	**tBuBorph**	0.5	0.718	1,2-DCE	Pentane	$LiNTf_2$	3	2.154
**2-46**	**tBuBorph**	0.5	0.718	1,2-DCE	Pentane	LiBArF	3	2.154
**2-47**	**tBuBorph**	0.5	0.718	1,2-DCE	Pentane	LiOTf	3	2.154
**2-48**	**tBuBorph**	0.5	0.718	1,2-DCE	Pentane	LiI	3	2.154

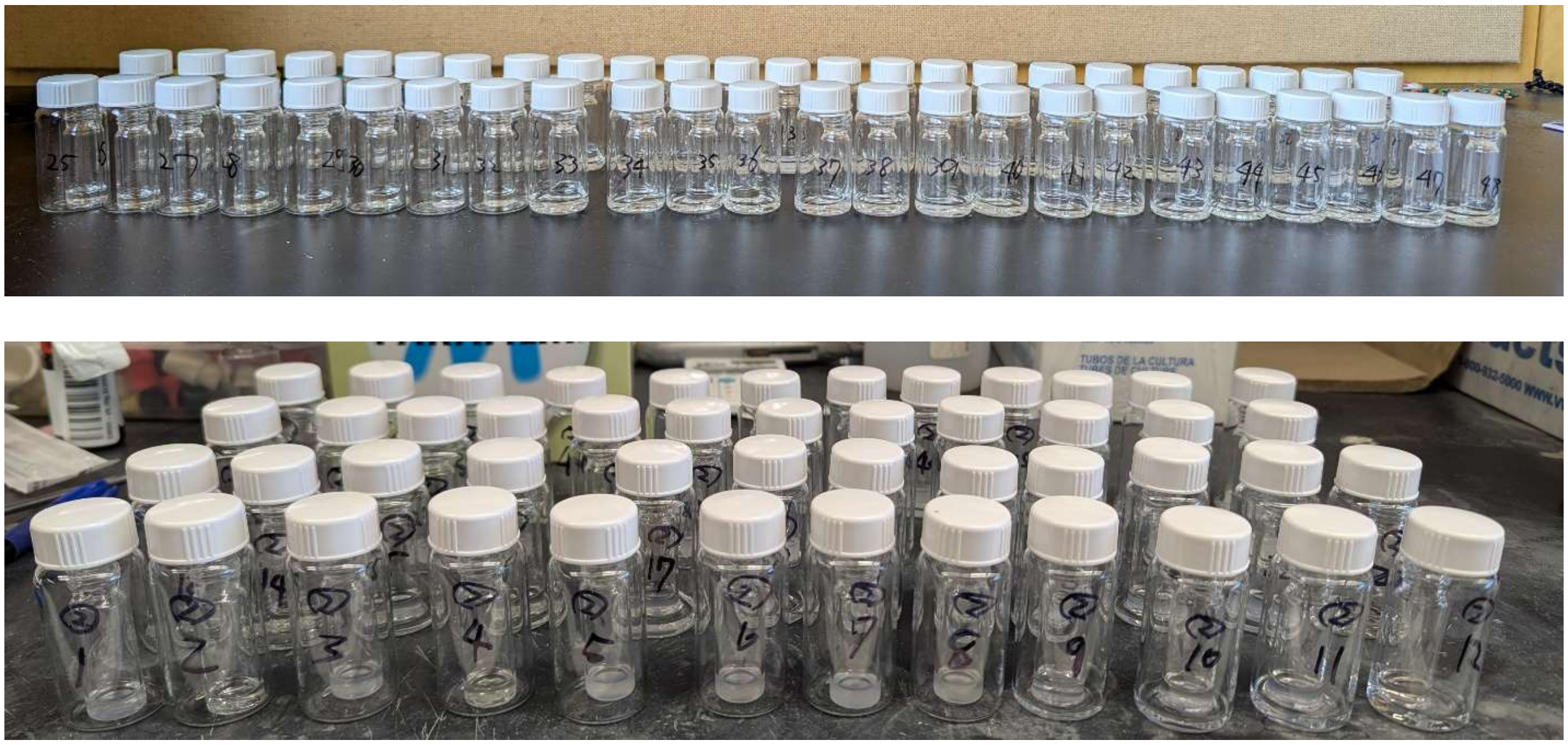

Fig. S17. Photograph of the full 96-sample crystallization screen for Li^{+}-bound borphyrin complexes prepared under dry and inert-atmosphere conditions.

<u>Control experiments to evaluate salt transfer into CPME</u>

A control sea-salt–mimicking solid mixture containing LiCl, NaCl, KCl, $MgSO_4$, and $CaSO_4$ was prepared based on ppm-equivalent cation mass concentration ratios of Li^{+}, Na^{+}, K^{+}, Mg^{2+}, and Ca^{2+} of 0.2 : 10500 : 380 : 1250 : 400, respectively, using the corresponding salts. The salts were thoroughly homogenized in deionized water, followed by evaporation to dryness in an oven overnight to ensure uniform distribution of all components. The resulting solid salt mixture was then subjected to extraction with CPME (10 mL) under vigorous stirring for 10 s, and this extraction procedure was repeated twice. The combined organic phases were subsequently back-extracted with 2% HNO_3 (5 mL × 2). The resulting aqueous extracts were analyzed by ICP-MS to quantify the amount of each metal cation transferred during the extraction. All other ICP-MS

experiments described in this work were conducted under analogous conditions using CPME as the extraction solvent, either in the absence or presence of borphyrin, to enable direct comparison of metal ion transfer with and without host-mediated complexation. Extracted ion percentages were calculated relative to the total amount of each cation initially present in the salt mixture.

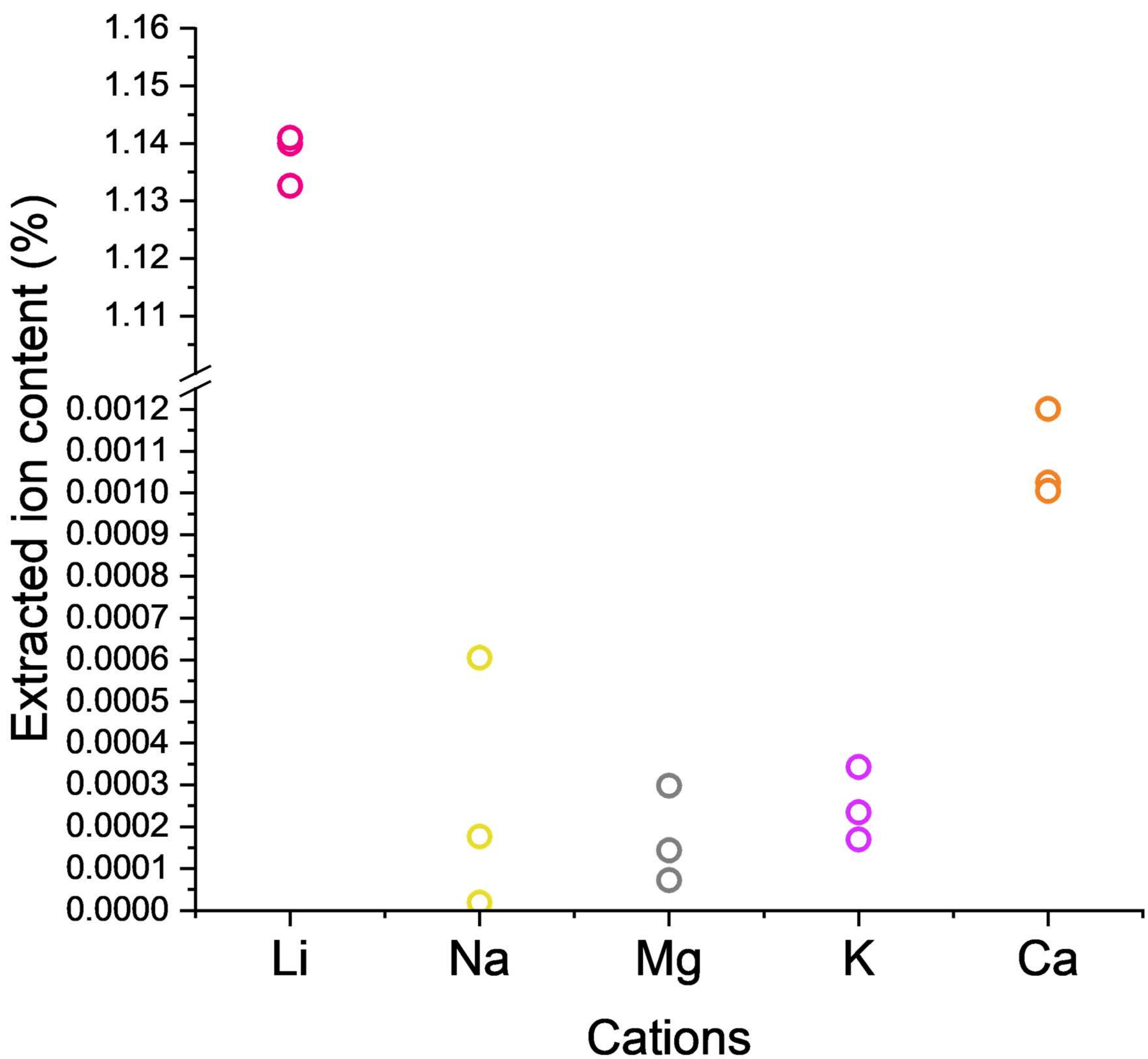

Fig. S18. Ion-content extracted percentage of alkali and alkaline earth metal cations extracted into CPME in the absence of borphyrin, calculated relative to the initial amount of each cation present in the salt mixture (initial Li^+ loading ≈ 4.79 ppm). Because the percentages are referenced to this low initial concentration, even a value of 1% corresponds to only ~0.048 ppm (~48 ppb) in absolute concentration. It is noteworthy that, all ions exhibit negligible solubility under the extraction conditions. The apparently higher relative value observed for Li^+ arises from its low initial loading, which places the measured Li^+ signal near the ICP-MS detection limit; this does not indicate appreciable lithium solubility in CPME in the absence of borphyrin.

Table S4. Ion-containing percentage of alkali and alkaline earth metal cations extracted into CPME in the absence of borphyrin.

Cation	Trial 1 (%)	Trial 2 (%)	Trial 3 (%)	Average (%)	S.D.
**Li^+**	1.14	1.14	1.13	1.14	0.013
**Na^+**	6.05×10^{-4}	1.77×10^{-4}	1.89×10^{-4}	2.67×10^{-4}	3.03×10^{-4}
**Mg^{2+}**	2.98×10^{-4}	1.44×10^{-4}	7.22×10^{-5}	1.71×10^{-4}	1.16×10^{-4}
**K^+**	3.43×10^{-4}	2.34×10^{-4}	1.69×10^{-4}	2.49×10^{-4}	8.78×10^{-5}
**Ca^{2+}**	1.20×10^{-3}	1.02×10^{-3}	1.01×10^{-3}	1.08×10^{-3}	1.08×10^{-4}

Protodeborylation of **tBuBorph** upon aqueous extraction

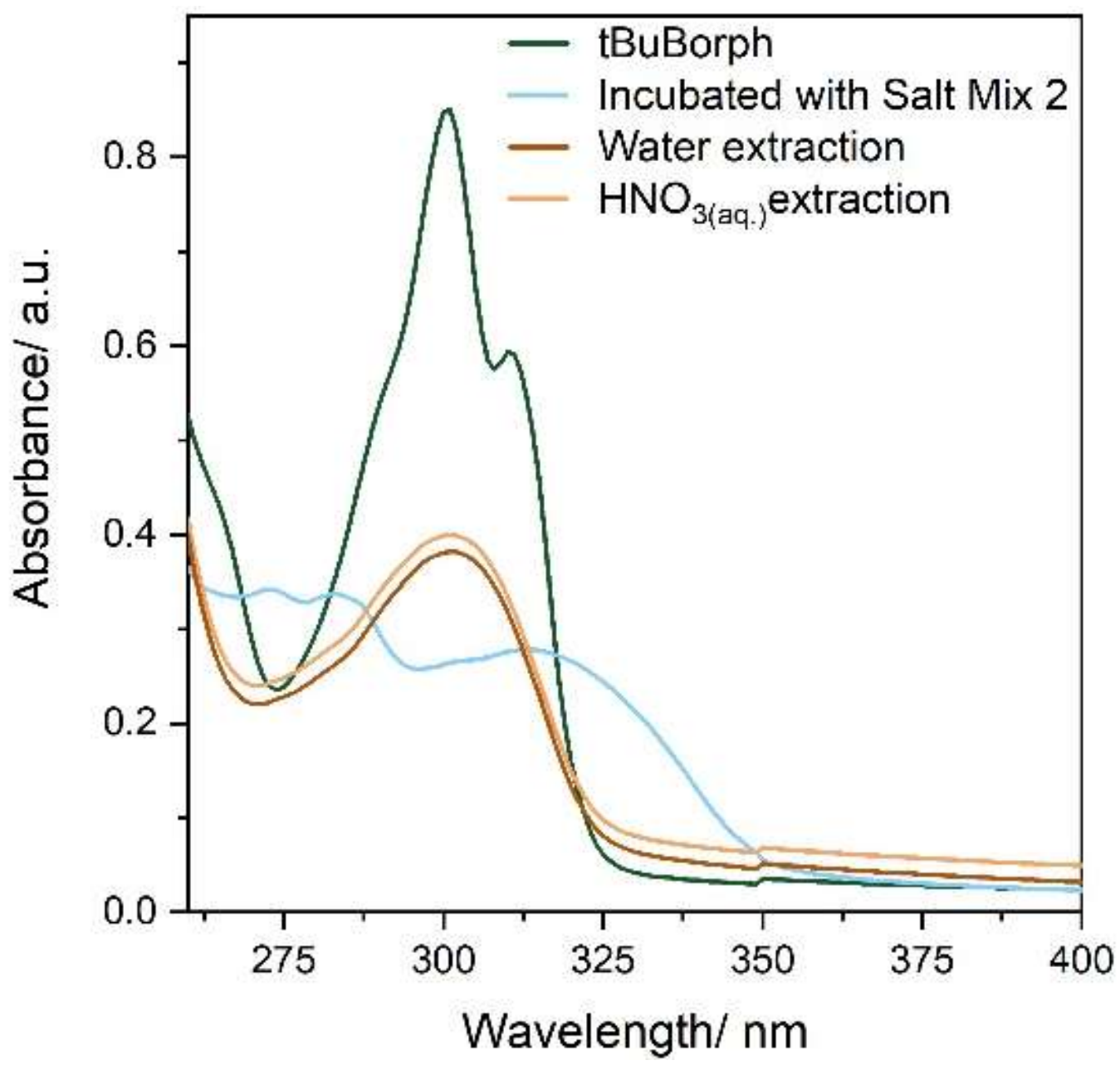

Fig. S19. UV–vis spectroscopy of **tBuBorph** ([**tBuBorph**] = 15 μM) in CPME (green) and upon exposure to Salt Mix 2 (alkali and alkaline earth metals; blue). The incubated solution was then extracted in water, and UV–Vis spectra of the organic phase show the decomposition of borphyrin into catechol (protodeborylation) upon extraction with water (maroon) or acidic solution (orange).

SCXRD analyses

HBorph

Crystal Data and Experimental

Experimental. Colorless needle-shaped crystals of **HBorph** were obtained from sublimation of **HBorph**. A suitable crystal with dimensions $0.63 \times 0.07 \times 0.03$ mm^3 was selected and mounted on a Bruker APEX-II CCD diffractometer. The crystal was kept at a steady T = 296.15(2) K during data collection. Data were measured using ω and ϕ scans of 0.5° per frame for 20 s using MoKα radiation (TRIUMPH monochromator, sealed X-ray tube, 45 kV, 30 mA). The total number of runs and images was based on the strategy calculation from the program APEX4. The maximum resolution that was achieved was Θ = 26.775° (0.82 Å). The structure was solved in the olex2.solve 1.5[22] solution program using iterative methods and by using Olex2[23] as the graphical interface. The model was refined with XL[24] using full matrix least squares minimization on F^2.

Crystal Data. $C_{12}H_6B_2O_4$, M_r = 235.79, triclinic, P-1 (No. 2), a = 4.8545(7) Å, b = 9.491(2) Å, c = 11.6351(16) Å, a = 111.023(7)°, b = 90.650(4)°, g = 98.320(6)°, V = 493.99(15) Å^3, T = 296.15 K, Z = 2, Z' = 1, m(MoK$_a$) = 0.115, 10227 reflections measured, 1869 unique (R_{int} = 0.0281) which were used in all calculations. The final wR_2 was 0.2276 (all data) and R_1 was 0.0848 (I≥2s(I)).

Structure solution and refinement

The unit cell was refined using SAINT V8.40B[25] on 654 reflections, 6% of the observed reflections. The final completeness is 99.20 % out to 25.775° in Θ.

SADABS-2016/2[26] was used for absorption correction. wR_2 (int) was 0.0530 before and 0.0411 after correction. The ratio of minimum to maximum transmission is 0.8307. The $\lambda/2$ correction factor is not present. The absorption coefficient μ of this material is 0.115 mm^{-1} at this wavelength (λ = 0.71073 Å) and the minimum and maximum transmissions are 0.619 and 0.745.

The structure was solved and the space group *P-1* (# 2) determined by the olex2.solve 1.5[22] structure solution program using dual methods and refined by full matrix least squares minimization on F^2 using version 2019/3 of XL[24]. All non-hydrogen atoms were refined anisotropically. Hydrogen atom positions were calculated geometrically and refined using the riding model.

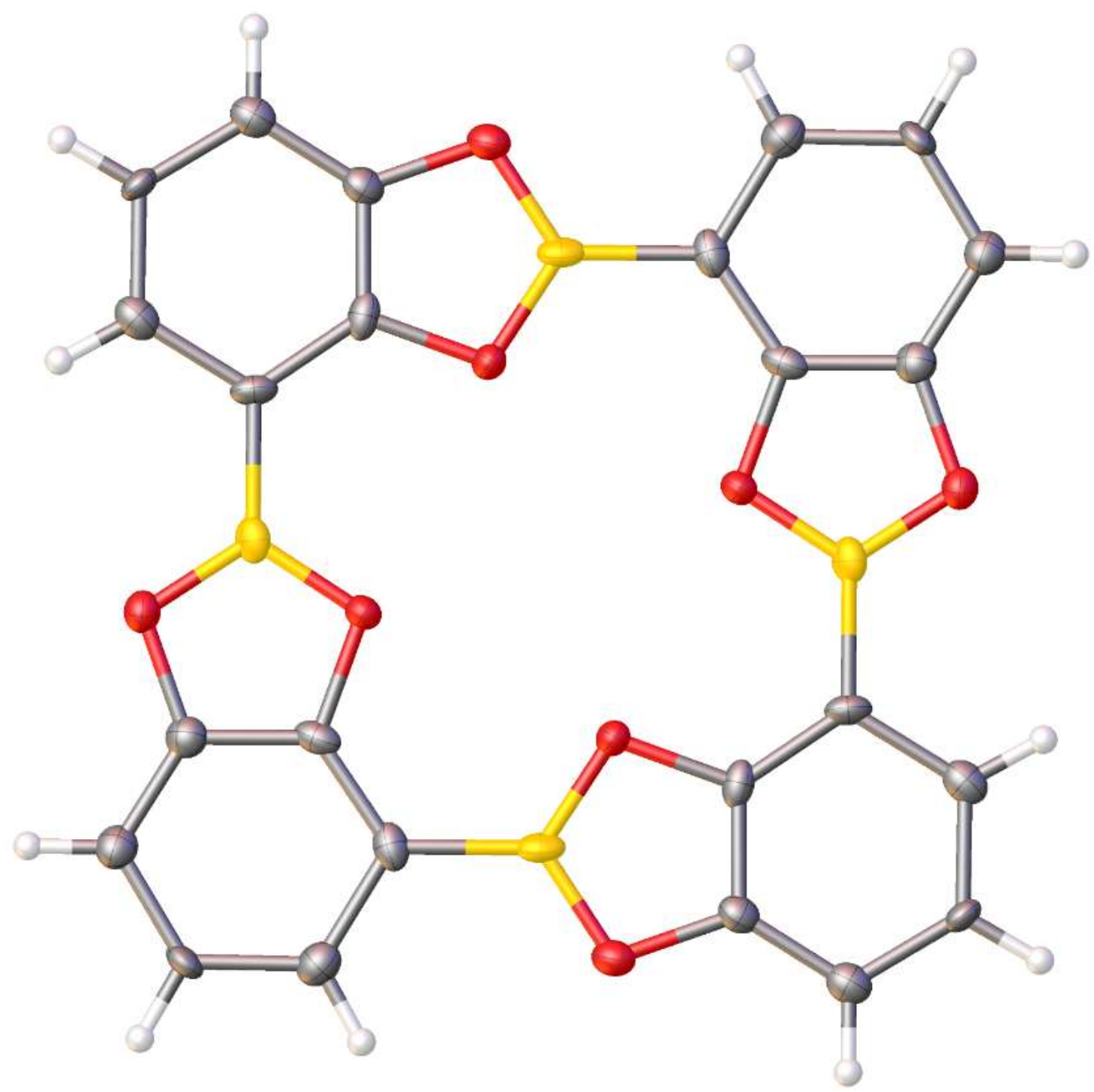

Fig. S20. Molecular structure of **HBorph** as determined by SCXRD. Ellipsoids are plotted at 50% probability level.

tBuBorph

Crystal Data and Experimental

Experimental. Colorless needle-shaped crystals of **tBuBorph** were obtained from sublimation of **tBuBorph**. A suitable crystal with dimensions 0.34 × 0.04 × 0.03 mm^3 was selected and mounted on a Bruker D8 Venture diffractometer. The crystal was kept at a steady T = 100(2) K during data collection. Data were measured using ω and ϕ scans of 1° per frame for 20 s using CuKα radiation (microfocus sealed X-ray tube, 50 kV, 1.0 mA). The total number of runs and images was based on the strategy calculation from the program APEX5. The maximum resolution that was achieved was Θ = 68.980° (0.83 Å). The structure was solved in the olex2.solve 1.5[22] solution program

using iterative methods and by using Olex2[23] as the graphical interface. The model was refined with XL[24] using full matrix least squares minimization on F^2.

Crystal Data. $C_{40}H_{44}B_4O_8$, M_r = 695.99, orthorhombic, *Ima*2 (No. 46), a = 6.6811(2) Å, b = 18.6863(6) Å, c = 31.4105(12) Å, $a = b = g = 90^{\circ}$, V = 3921.4(2) Å^3, T = 100(2) K, Z = 4, Z' = 0.5, m(Cu K_a) = 0.635, 33235 reflections measured, 3946 unique (R_{int} = 0.0956) which were used in all calculations. The final wR_2 was 0.1713 (all data) and R_1 was 0.0496 (I≥2s(I)).

Structure solution and refinement

The unit cell was refined using APEX 5 on 8896 reflections, 27% of the observed reflections. The final completeness is 99.20 % out to 25.775° in Θ.

SADABS-2016/2[26] was used for absorption correction. wR_2 (int) was 0.1512 before and 0.0906 after correction. The ratio of minimum to maximum transmission is 0.8737. The λ /2 correction factor is not present. The absorption coefficient μ of this material is 0.635 mm^{-1} at this wavelength (λ = 1.54178 Å) and the minimum and maximum transmissions are 0.857 and 0.981.

The structure was solved and the space group *Ima2* (# 46) determined by the XT[24] structure solution program using dual methods and refined by full matrix least squares minimization on F^2 using version 2019/3 of XL[24]. All non-hydrogen atoms were refined anisotropically. Hydrogen atom positions were calculated geometrically and refined using the riding model. Refined as a 2-component inversion twin.

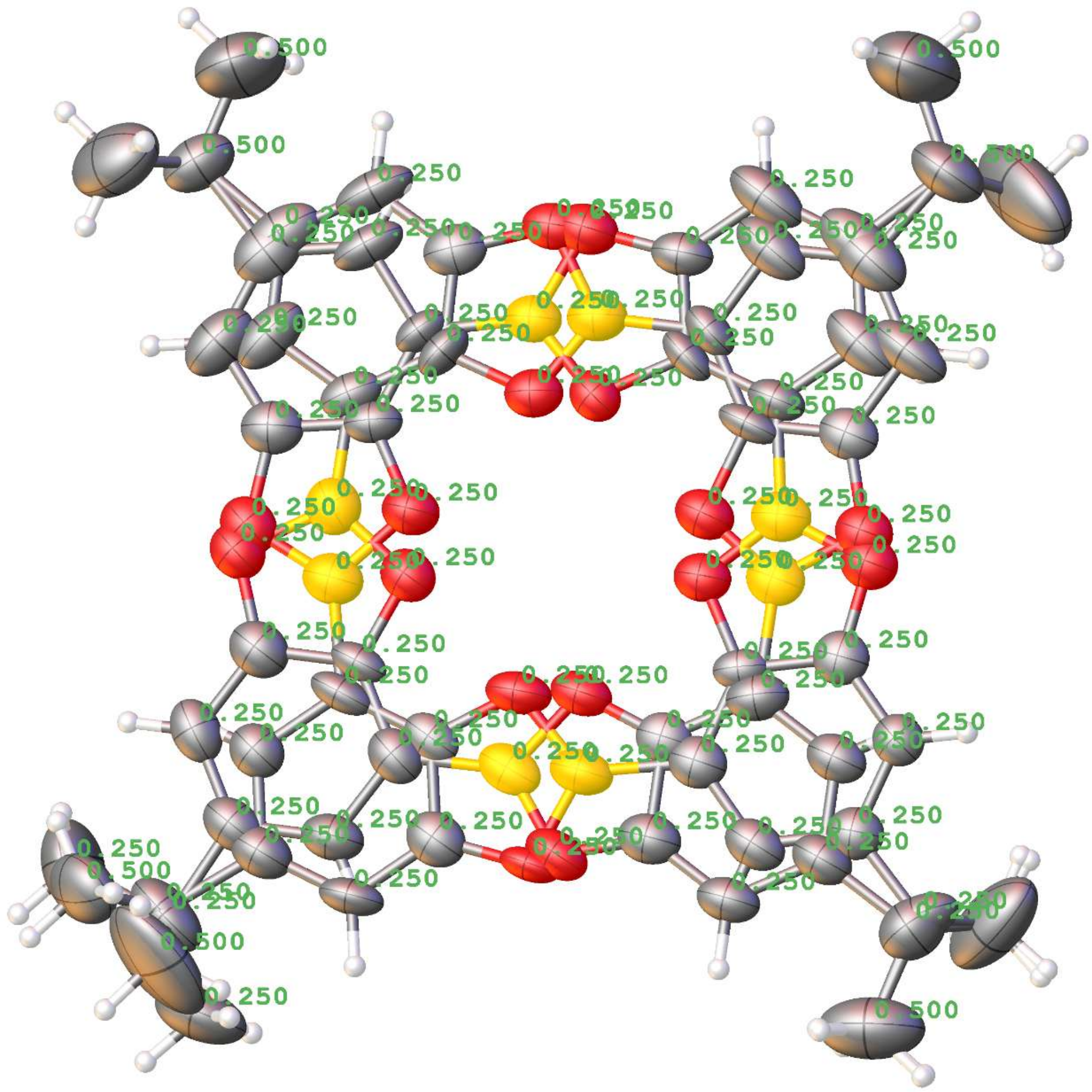

Fig. S21. Whole-molecule disorder in the crystal lattice of **tBuBorph** as determined by single-crystal X-ray diffraction, showing a 50:50 superposition of clockwise and counter-clockwise orientations of the macrocycle. Ellipsoids are plotted at 50% probability level.

STBorph

Crystal Data and Experimental

Experimental. Single colourless plate-shaped crystals of **STBorph** recrystallized from toluene by slow evaporation. A suitable crystal with dimensions $0.14 \times 0.07 \times 0.03$ mm^3 was selected and mounted on a Bruker APEX-II area detector diffractometer. The crystal was kept at a steady T = 100(2) K during data collection. Data were measured using ω and ϕ scans of 1° per frame for 20 s using CuKα radiation (Incoatec IμS Diamond II microfocus, sealed X-ray tube, 50 kV, 1.0 mA). The total number of runs and images was based on the strategy calculation from the program APEX4. The maximum resolution that was achieved was Θ = 56.041° (0.93 Å). The structure was solved in the XT[24] solution program using iterative methods and by using Olex2[23] as the graphical interface. The model was refined with XL[24] using full matrix least squares minimization on F^2.

Crystal Data. $C_{95}H_{106}B_2O_4$, M_r = 1333.41, triclinic, P-1 (No. 2), a = 15.4417(11) Å, b = 15.9529(11) Å, c = 19.1145(13) Å, a = 85.006(5)°, b = 75.458(5)°, g = 61.468(5)°, V = 4001.3(5) Å^3, T = 100(2) K, Z = 2, Z' = 1, m(CuK$_a$) = 0.495, 47774 reflections measured, 10404 unique (R_{int} = 0.2124) which were used in all calculations. The final wR_2 was 0.2563 (all data) and R_1 was 0.0868 (I≥2s(I)).

Structure solution and refinement

The unit cell was refined using SAINT V8.40B[25] on 3511 reflections, 7% of the observed reflections. The final completeness is 99.70 % out to 56.041° in Θ.

SADABS-2016/2[26] was used for absorption correction. wR_2 (int) was 0.0989 before and 0.0787 after correction. The ratio of minimum to maximum transmission is 0.8360. The λ /2 correction

factor is not present. The absorption coefficient μ of this material is 0.495 mm^{-1} at this wavelength (λ = 1.54178 Å) and the minimum and maximum transmissions are 0.824 and 0.985.

The structure was solved and the space group *P-1* (# 2) determined by the XT[24] structure solution program using dual methods and refined by full matrix least squares minimization on F^2 using version 2019/3 of XL[24]. All non-hydrogen atoms were refined anisotropically. Hydrogen atom positions were calculated geometrically and refined using the riding model.

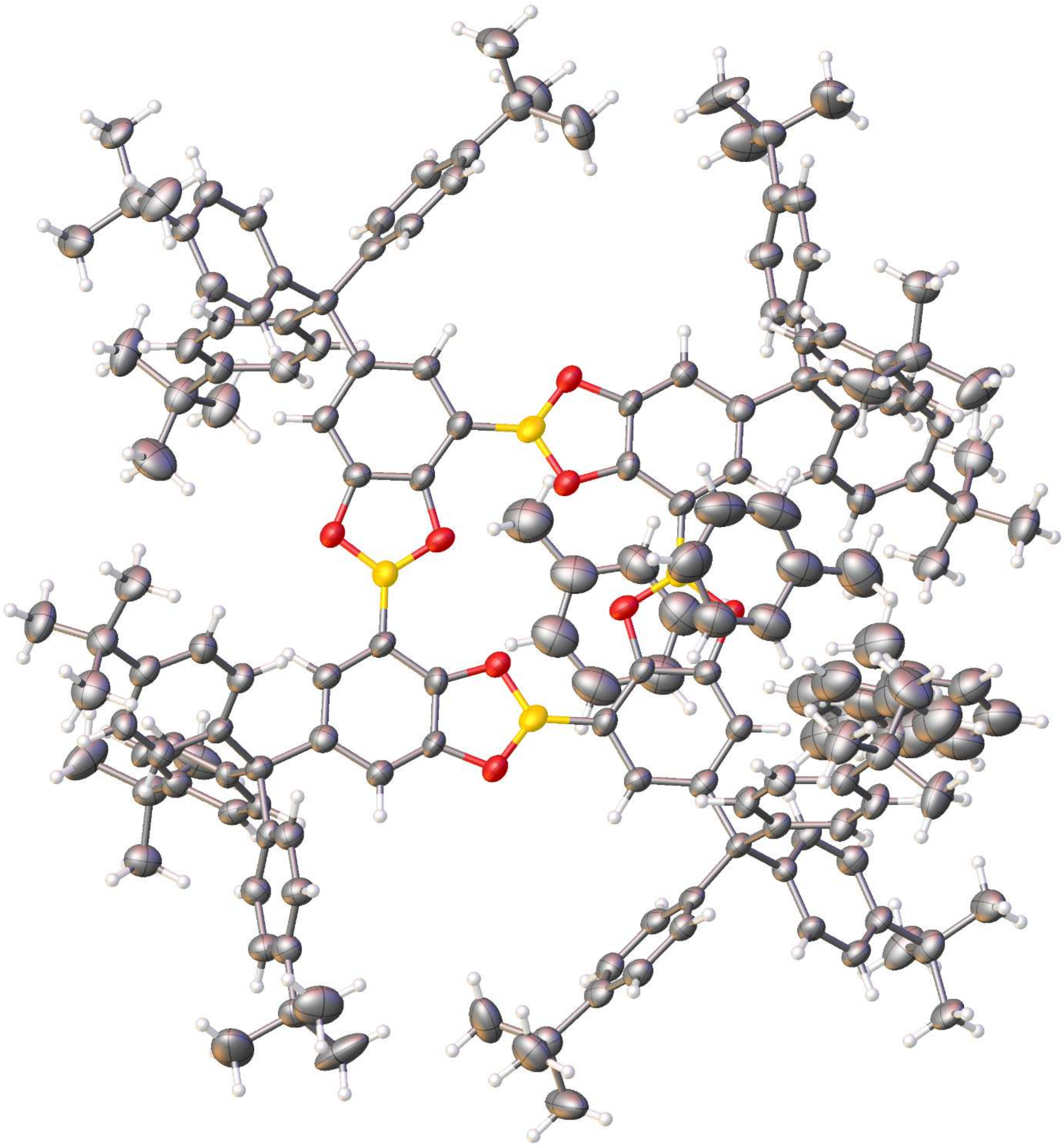

Fig. S22. Molecular structure of **STBorph** as determined by SCXRD. Ellipsoids are plotted at 50% probability level.

Table S5. Crystallographic structure refinement data for all borphyrins.

	**HBorph (mm581)**	**tBuBorph (mm647)**	**STBorph (mm617)**
Chemical formula	$C_{12}H_6B_2O_4$	$C_{40}H_{44}B_4O_8$	$C_{95}H_{106}B_2O_4$
Formula weight	235.79	695.99	1333.41
Crystal size (mm)	0.63 × 0.07 × 0.03	0.34 × 0.04 × 0.03	0.14 × 0.07 × 0.03
Temperature (K)	296.15 (2)	100(2)	100(2)
Crystal system	triclinic	orthorhombic	Triclinic
Space group	*P-1*	*Ima2*	*P*-1
Unit cell dimensions	a = 4.8545(7) Å<br>b = 9.491(2)Å<br>c = 11.6351(16)Å<br>*a* = 111.023(7)°<br>*β* = 90.650(4)°<br>γ = 98.320(6)°	*a* = 6.6811(2) Å<br>*b* = 18.6863(6) Å<br>*c* = 31.4105(12) Å<br>*a* = *β* = *γ* = 90°	*a* = 15.4417(11) Å<br>*b* = 15.9529(11) Å<br>*c* = 19.1145(13) Å<br>*a* = 85.006(5)°<br>*β* = 75.458(5)°<br>γ = 61.468(5)°
Unit Cell Volume($Å^3$)	493.99(15)	3921.4(2)	4001.3(5)
Z	2	4	2
Absorption coefficient (mm^{-1})	0.115	0.635	0.495
Total reflections collected	10227	33235	47774
Unique reflections	1869 [R_{int} = 0.0281]	3946 [R_{int} = 0.0956]	10404 [R_{int} = 0.2124]

Completeness to theta	25.775° 99.20%	25.775° 99.20%	56.041° 99.70%
Goodness-of-fit on F^2	1.044	1.048	0.983
Final R_1 value [$I>2\sigma(I)$]	0.0848	0.0495	0.0868
Final wR_2 value (all data)	0.2276	0.1713	0.2004

<u>Characterization and Analysis</u>

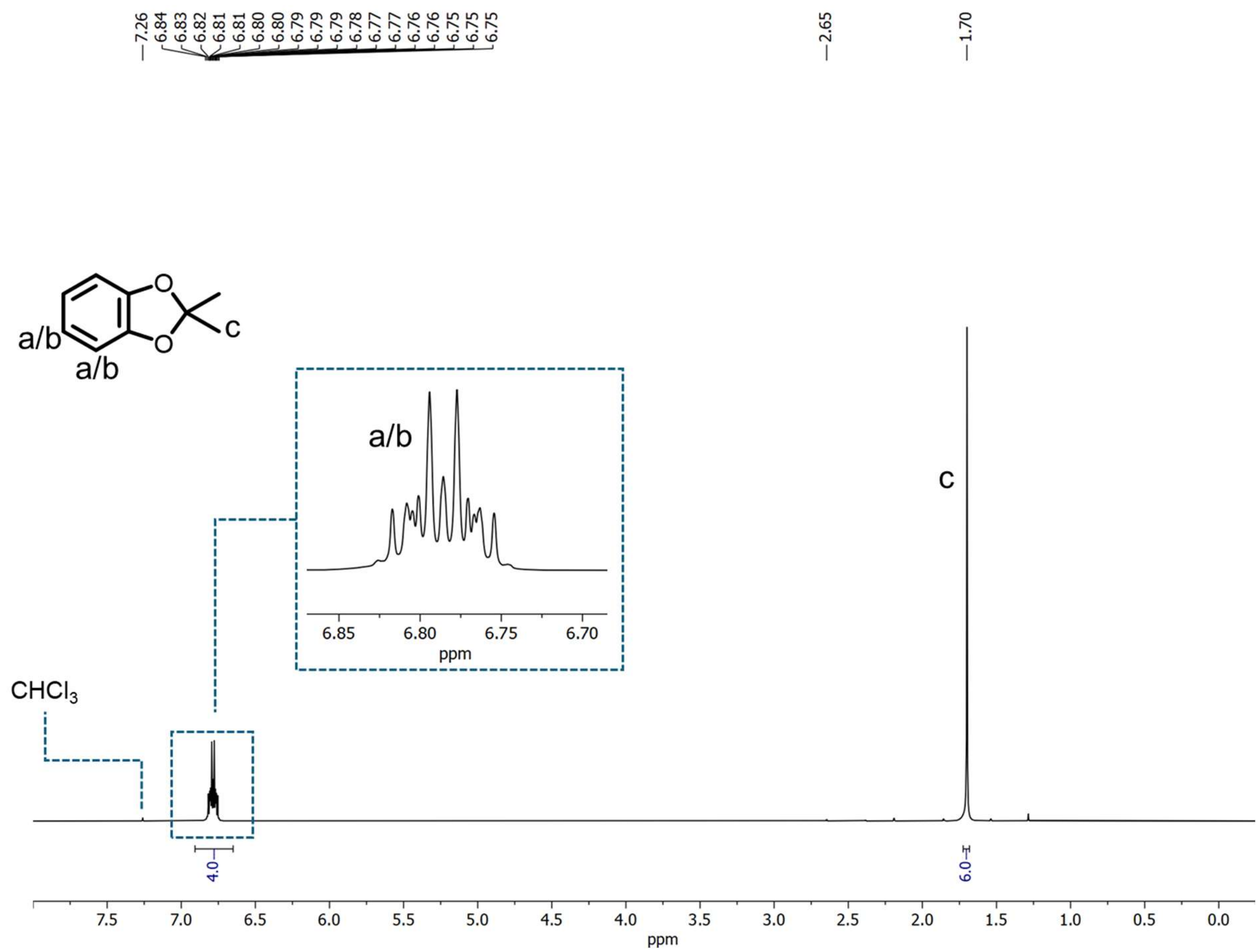

Fig. S23. ^{1}H NMR spectrum (400 MHz, chloroform-*d*) of **Ace-1**.

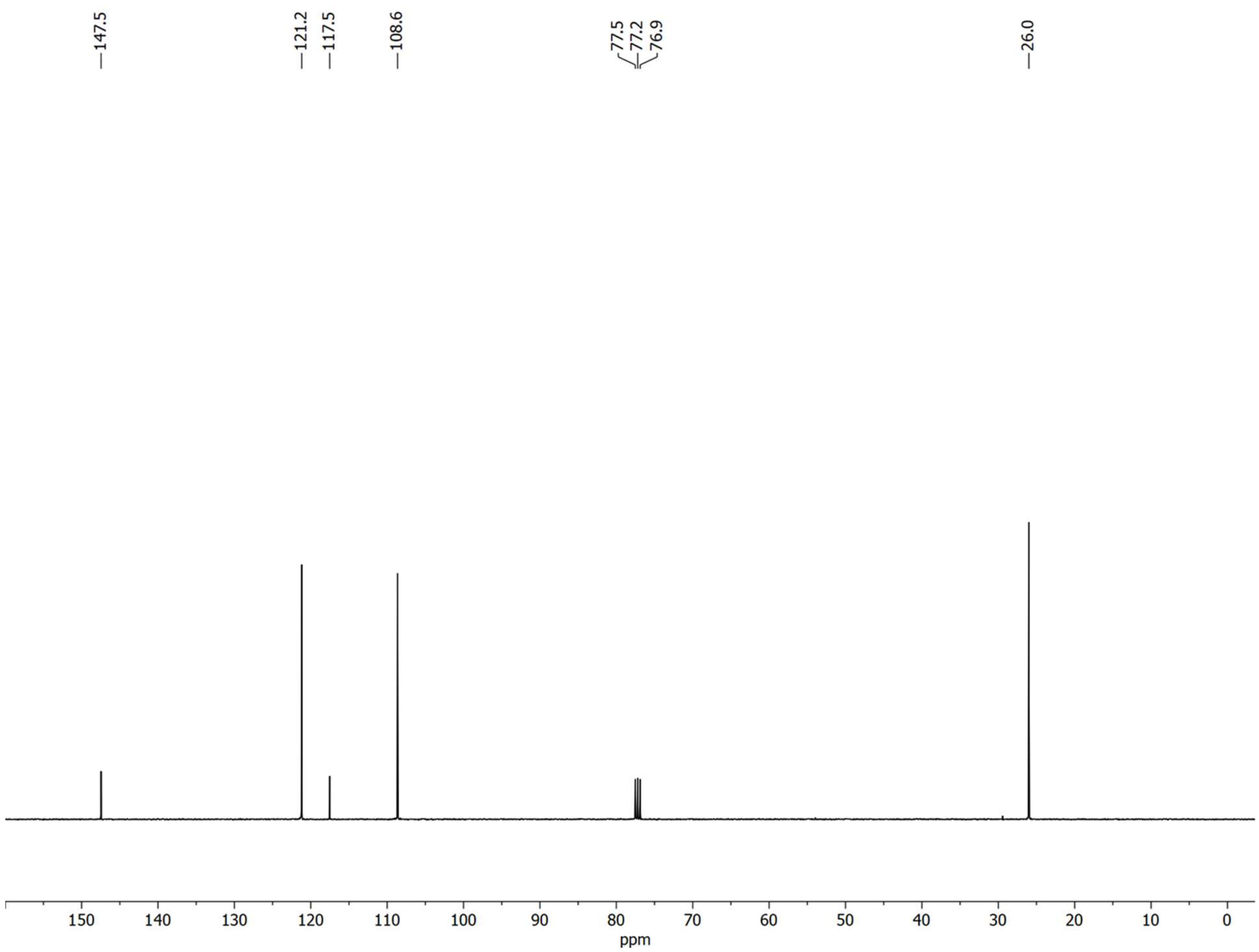

Fig. S24. $^{13}C\{^{1}H\}$ NMR spectrum (101 MHz, chloroform-*d*) of **Ace-1**.

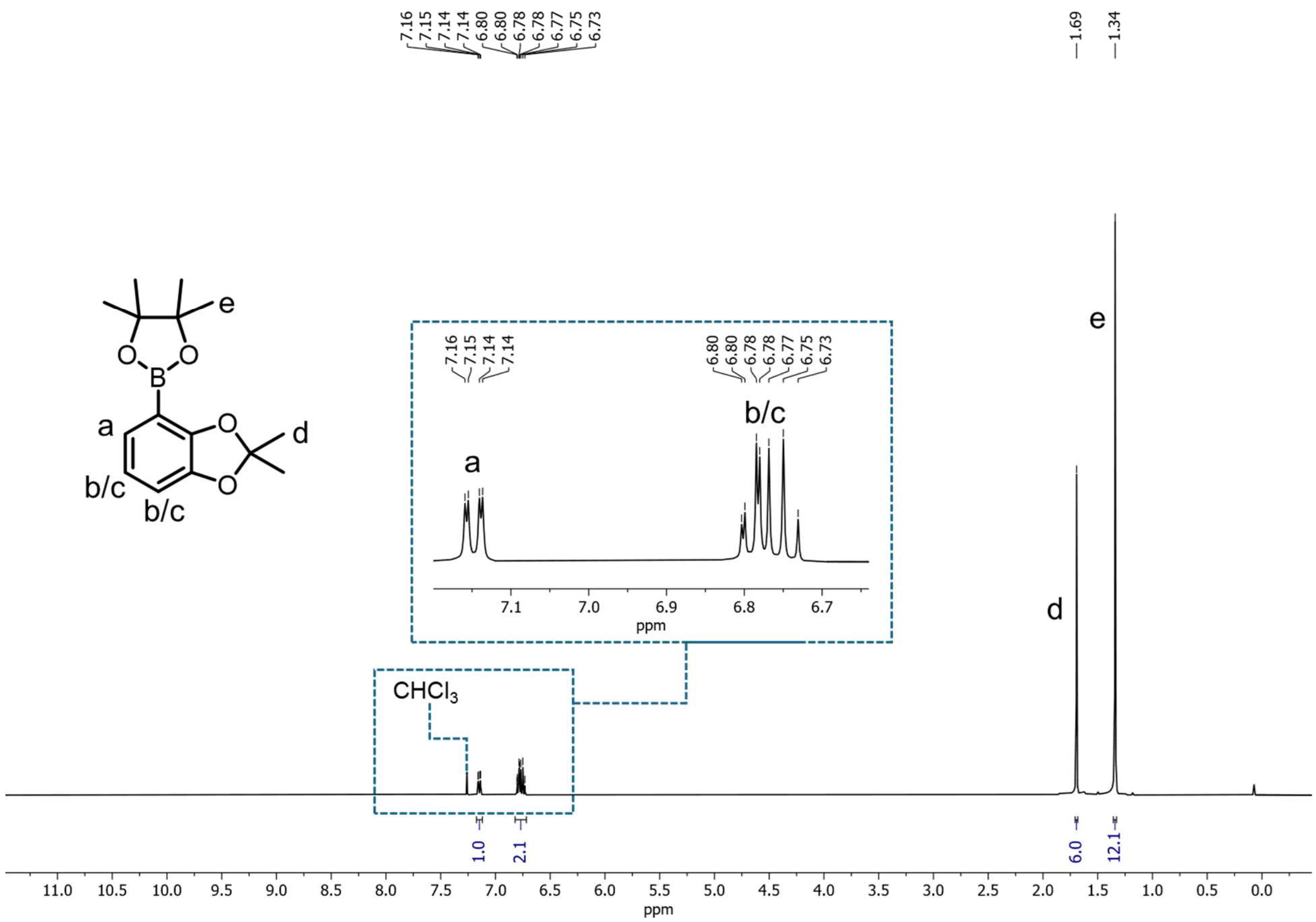

Fig. S25. ^{1}H NMR spectrum (400 MHz, chloroform-*d*) of **AceBpin-1**.

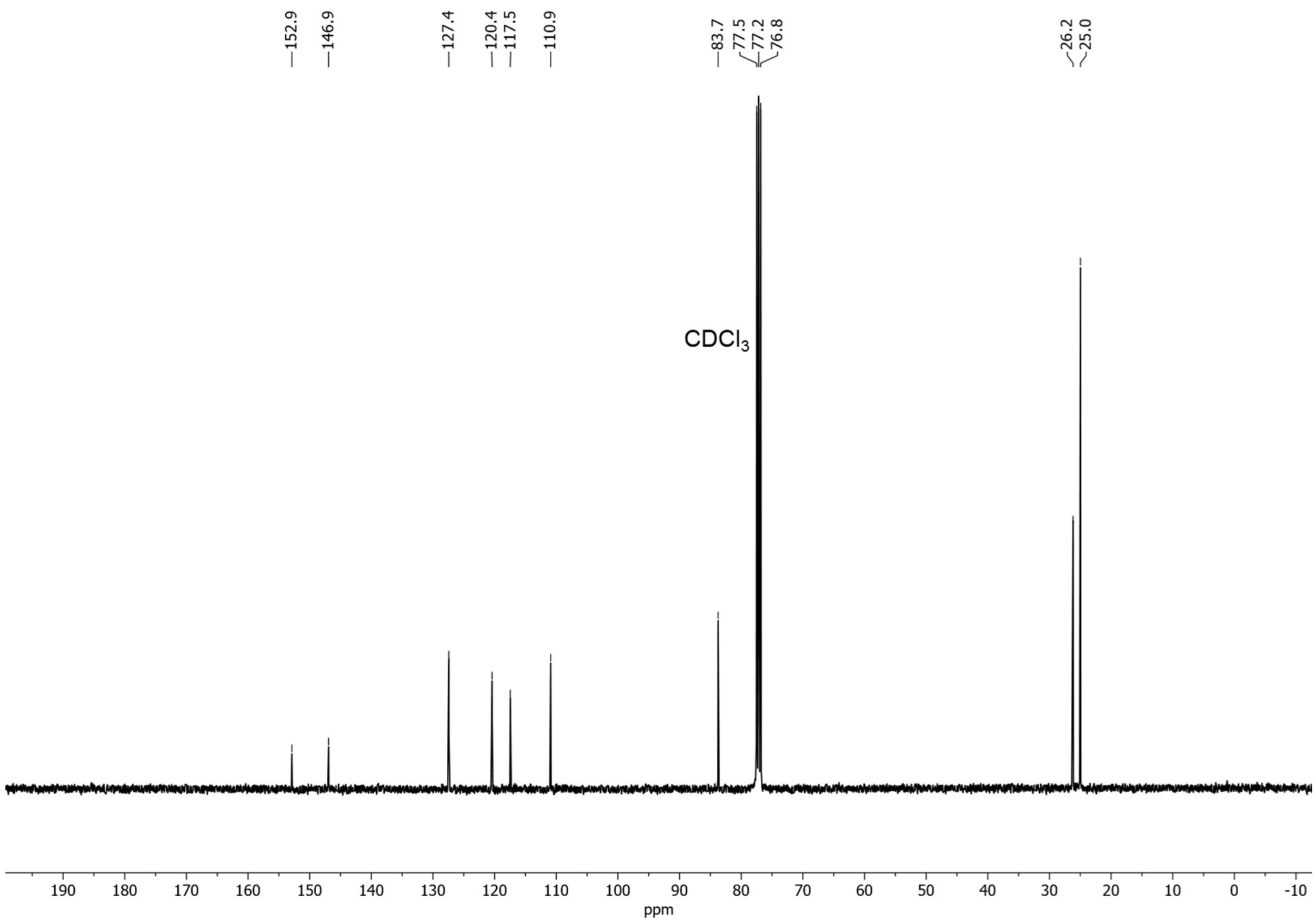

Fig. S26. $^{13}C\{^{1}H\}$ NMR spectrum (101 MHz, chloroform-*d*) of **AceBpin-1**.

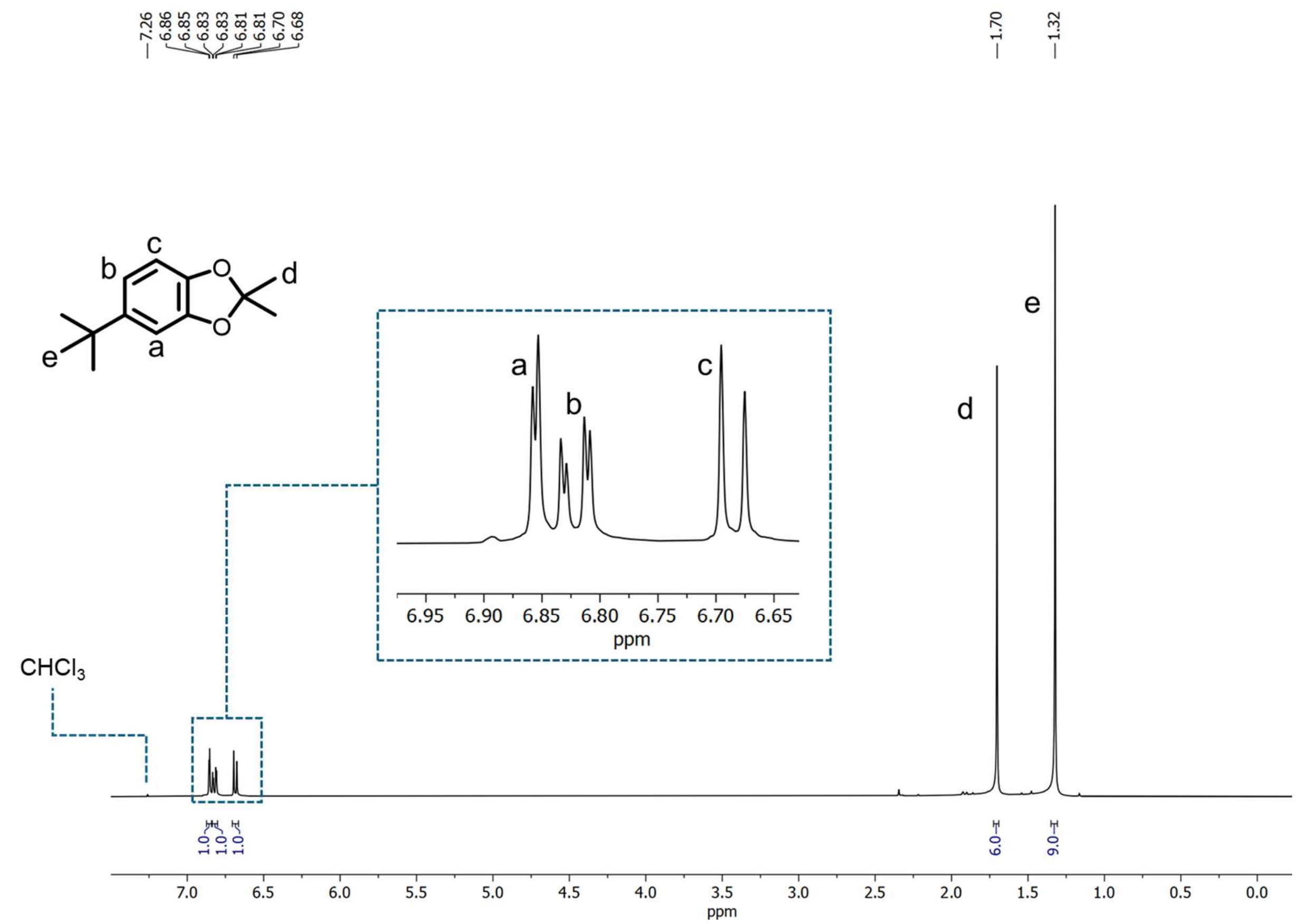

Fig. S27. ^{1}H NMR spectrum (400 MHz, chloroform-*d*) of **Ace-2**.

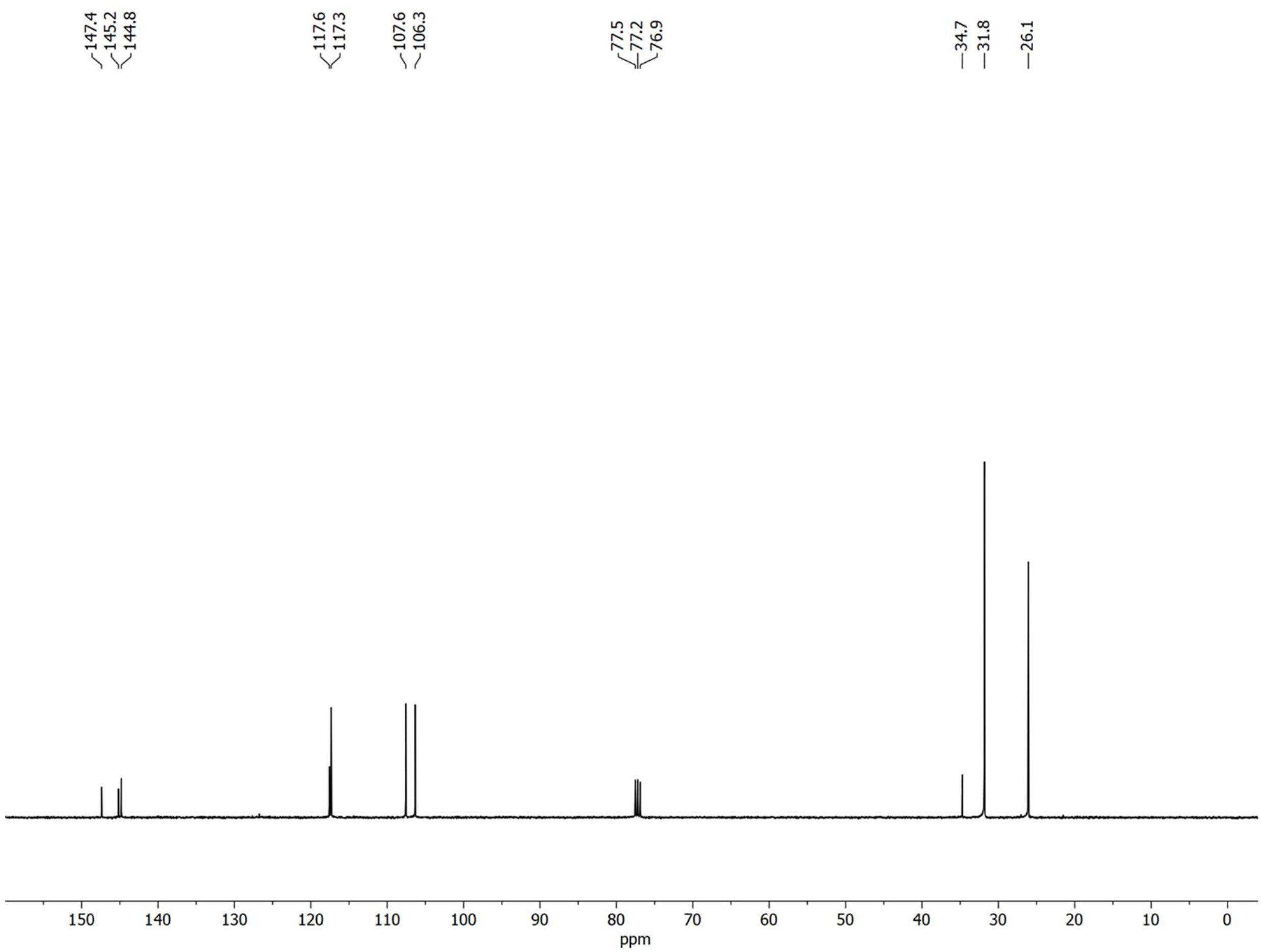

Fig. S28. $^{13}C\{^{1}H\}$ NMR spectrum (101 MHz, chloroform-*d*) of **Ace-2**.

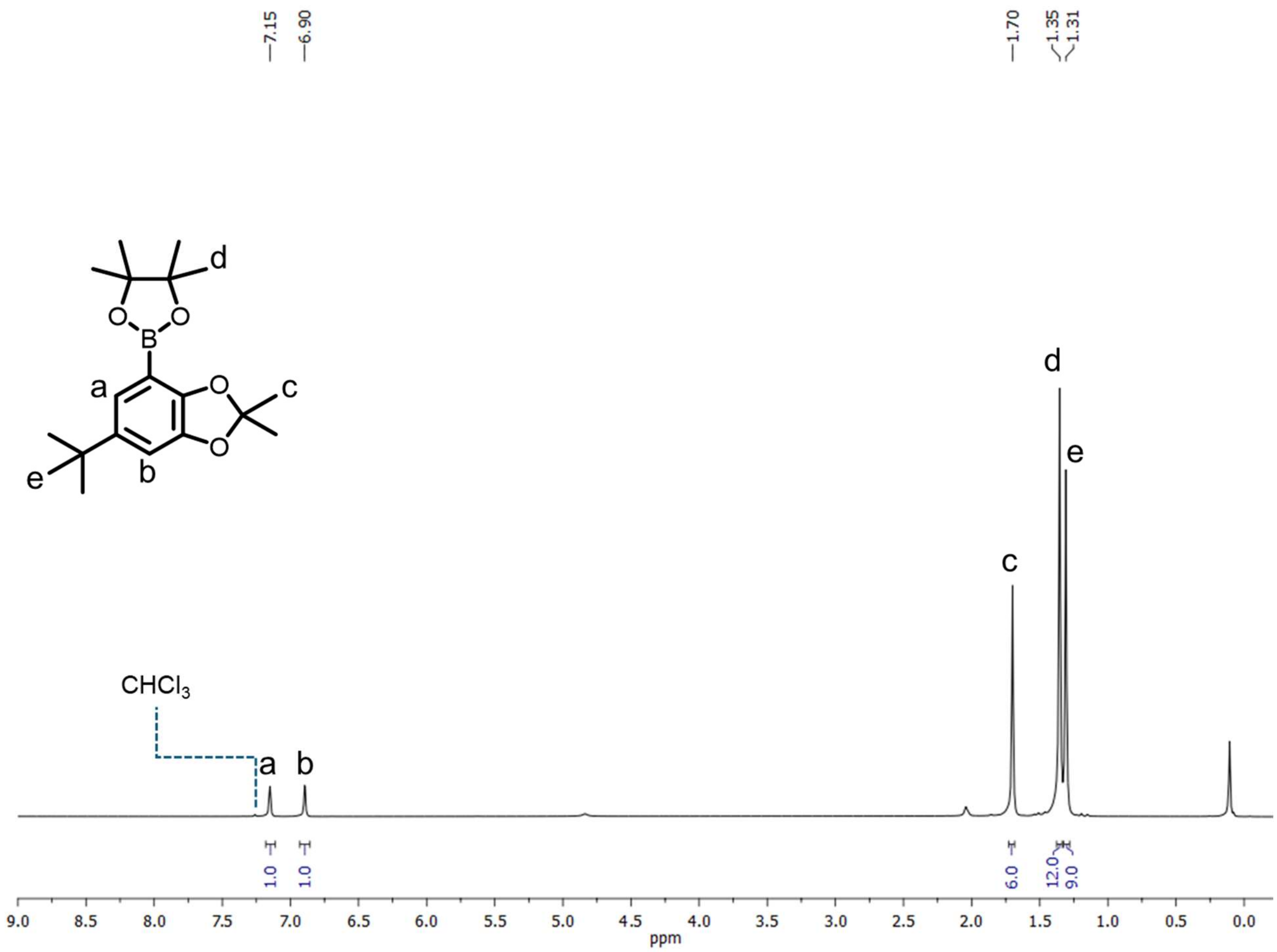

Fig. S29. ^{1}H NMR spectrum (400 MHz, chloroform-*d*) of **AceBpin-2**.

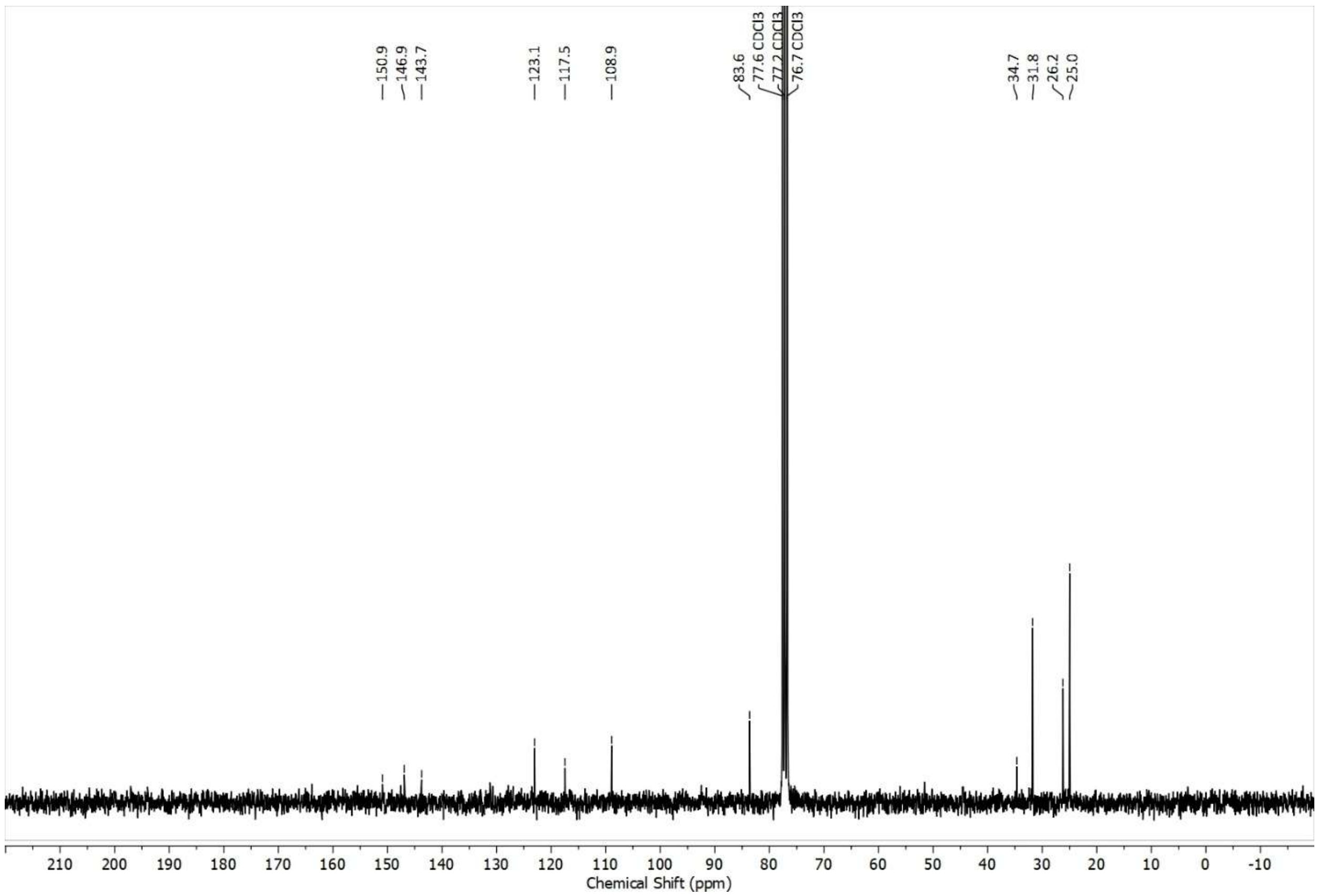

Fig. S30. $^{13}C\{^1H\}$ NMR spectrum (101 MHz, chloroform-*d*) of **AceBpin-2**.

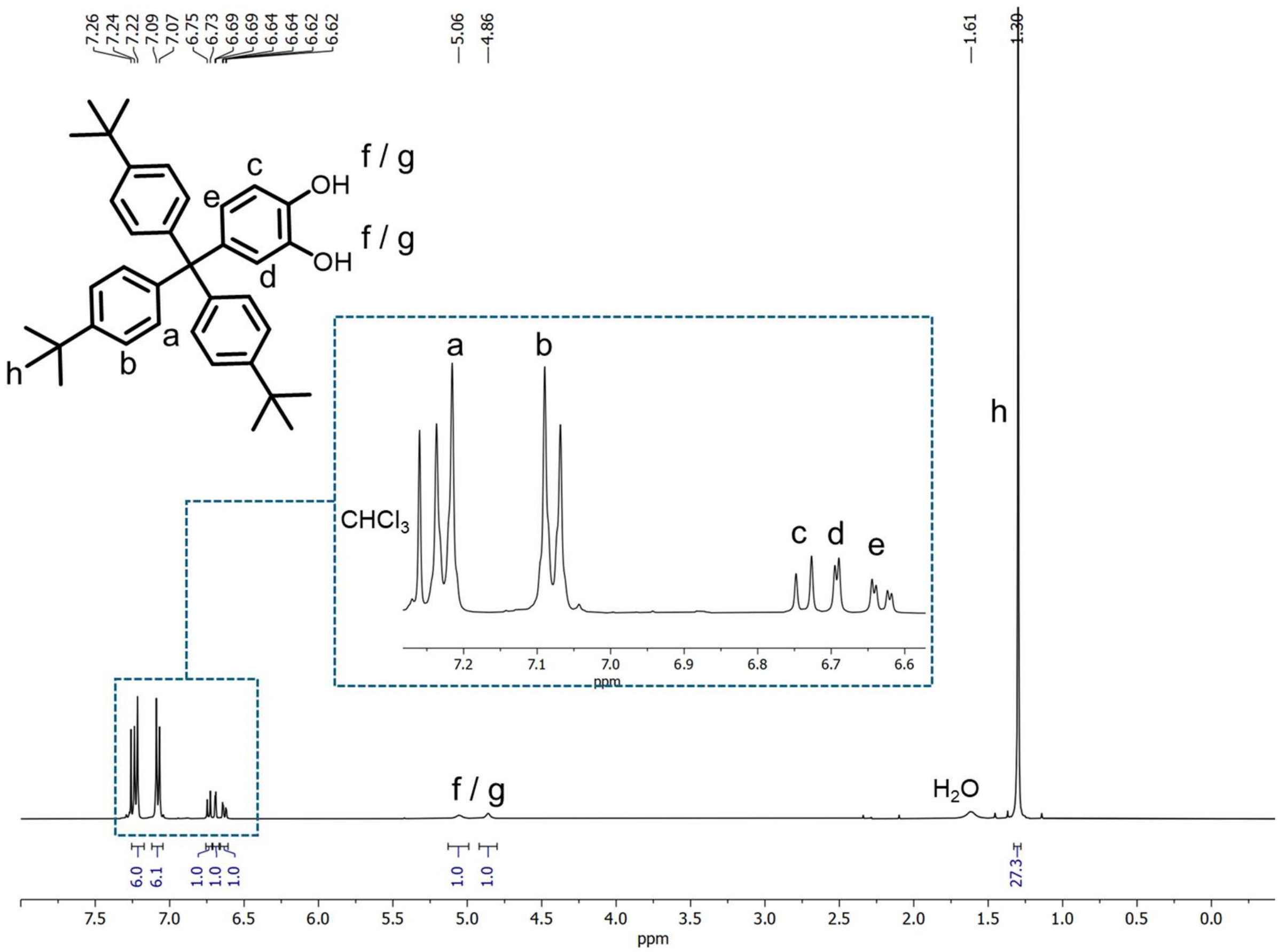

Fig. S31. ^{1}H NMR spectrum (400 MHz, chloroform-*d*) of **CAT-3**.

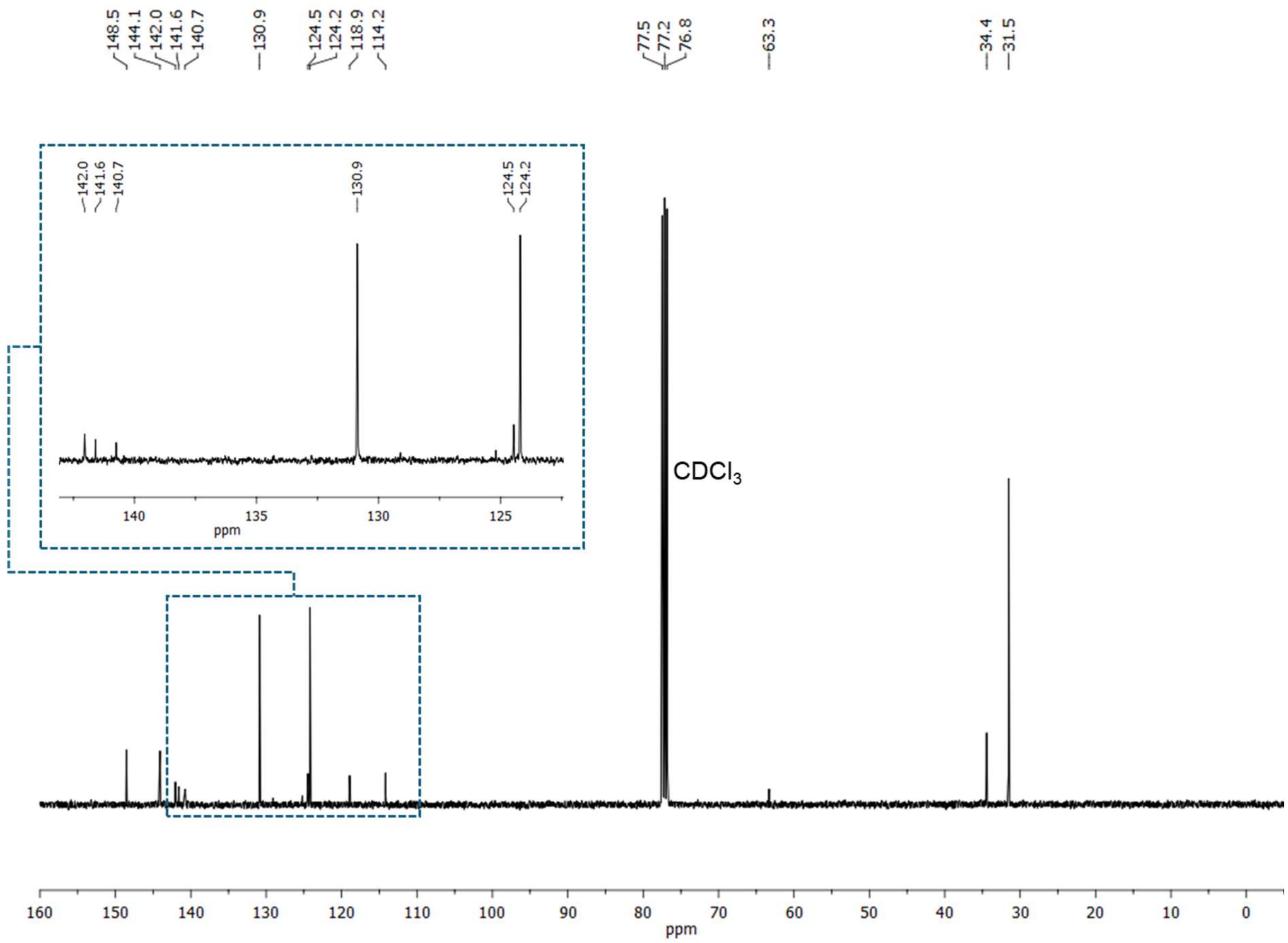

Fig. S32. $^{13}C\{^{1}H\}$ NMR spectrum (101 MHz, chloroform-*d*) of **CAT-3**.

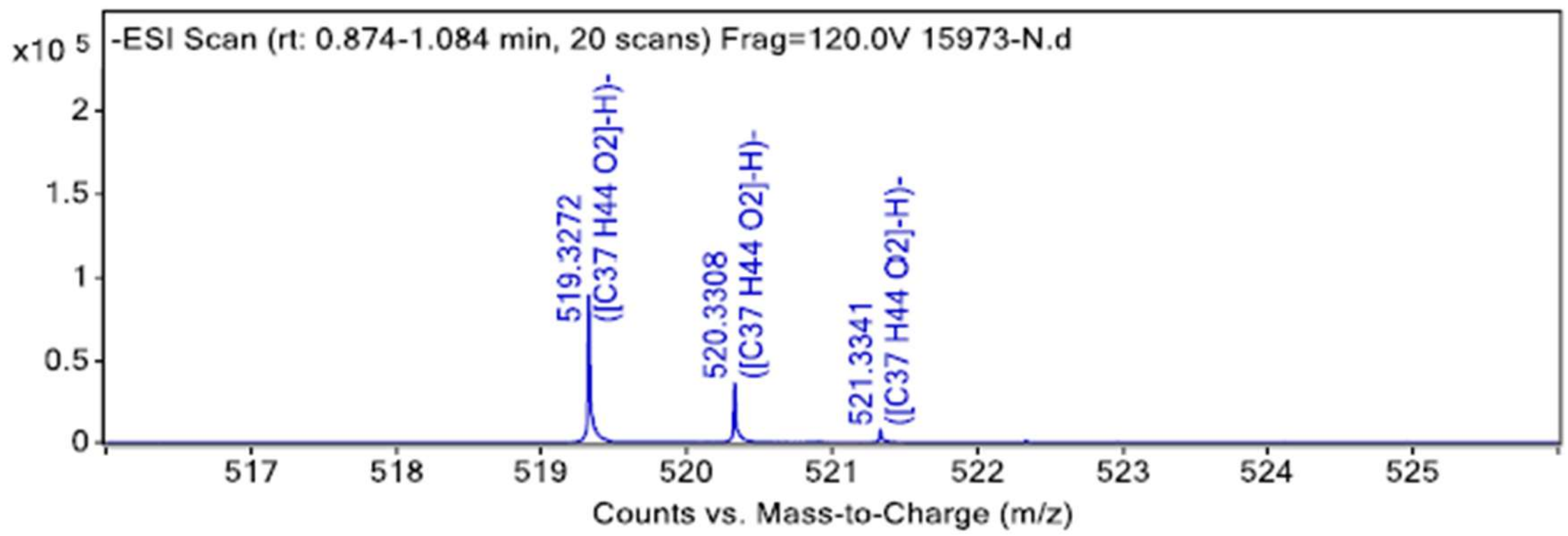

Fig. S33. High-resolution ESI-MS (HRMS) spectrum of **CAT-3**.

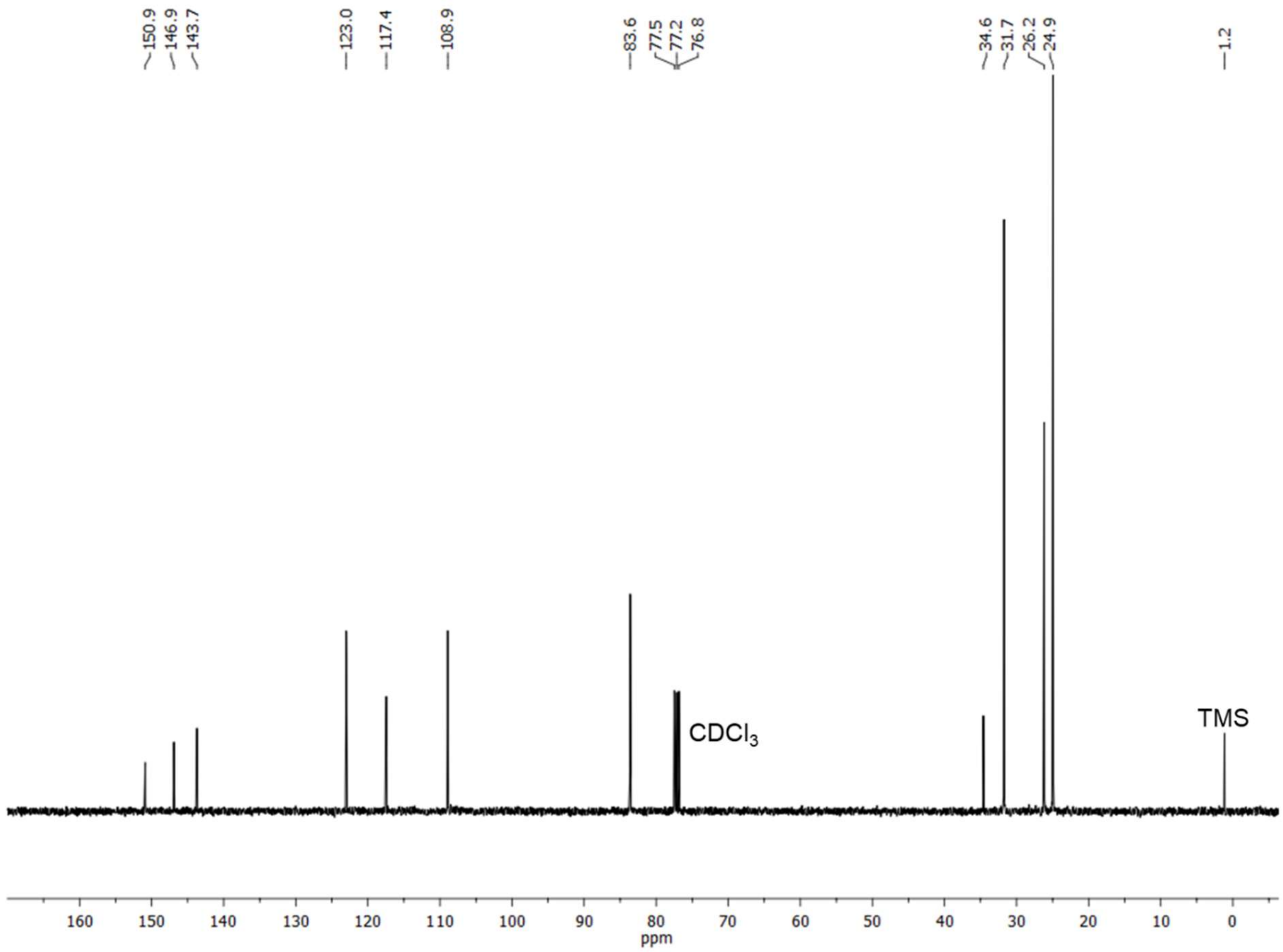

Fig. S34. $^{13}C\{^1H\}$ NMR spectrum (101 MHz, chloroform-*d*) of **AceBpin-2**.

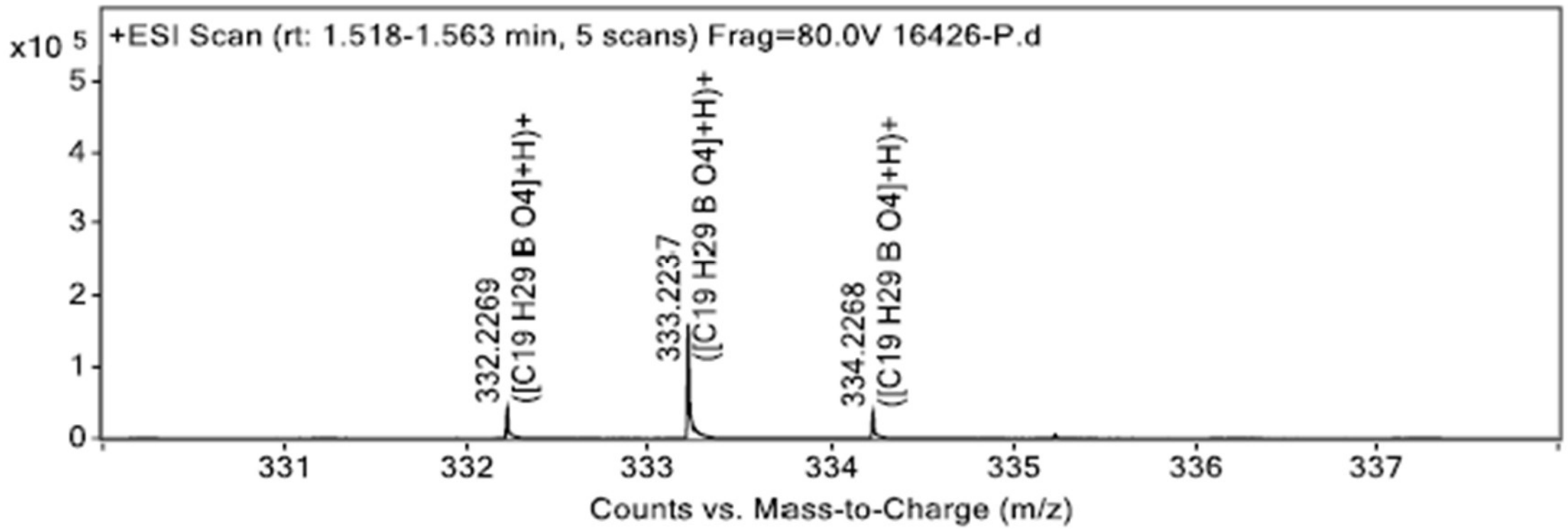

Fig. S35. High-resolution ESI-MS (HRMS) spectrum of **AceBpin-2**.

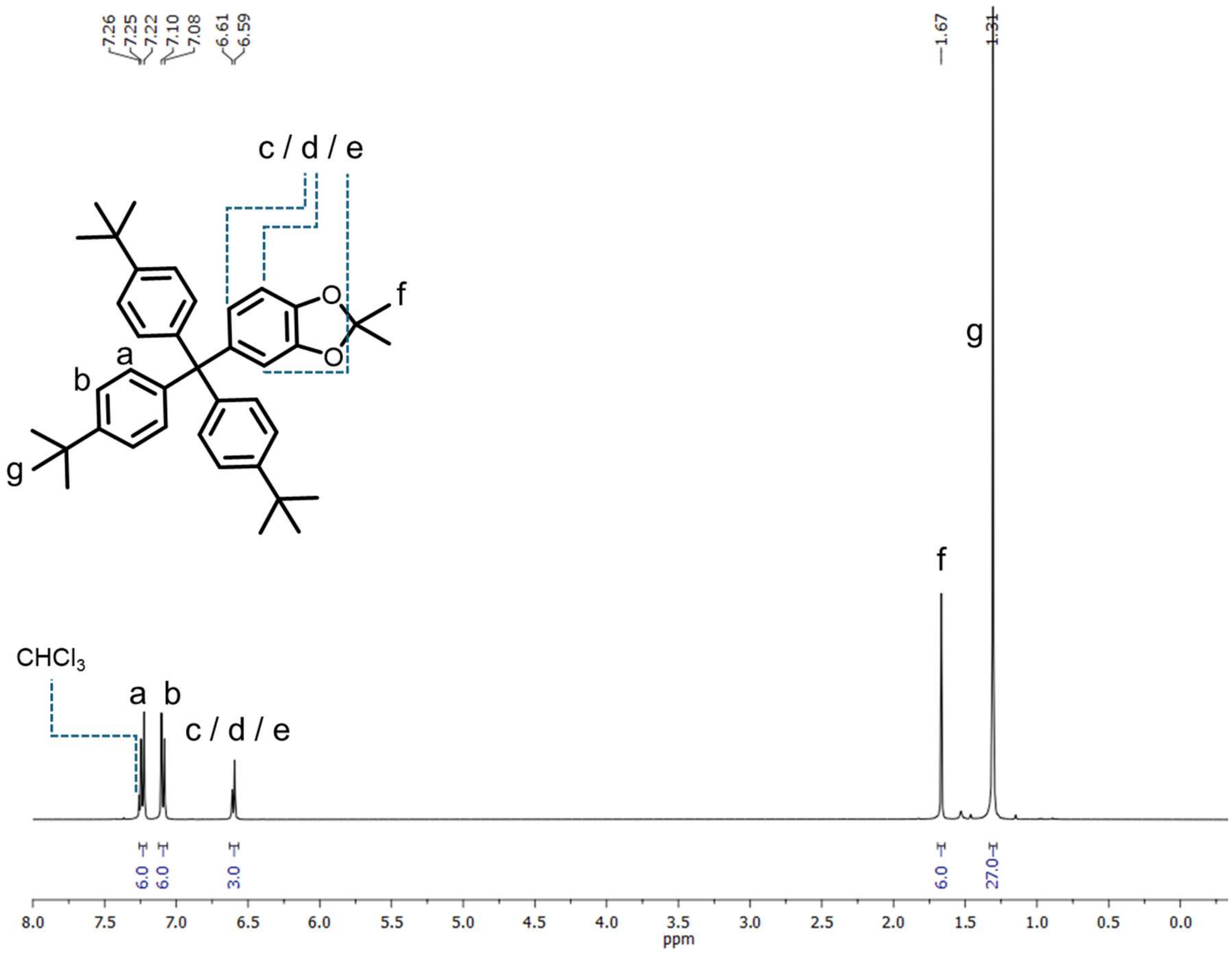

Fig. S36. ^{1}H NMR spectrum (400 MHz, chloroform-*d*) of **Ace-3**.

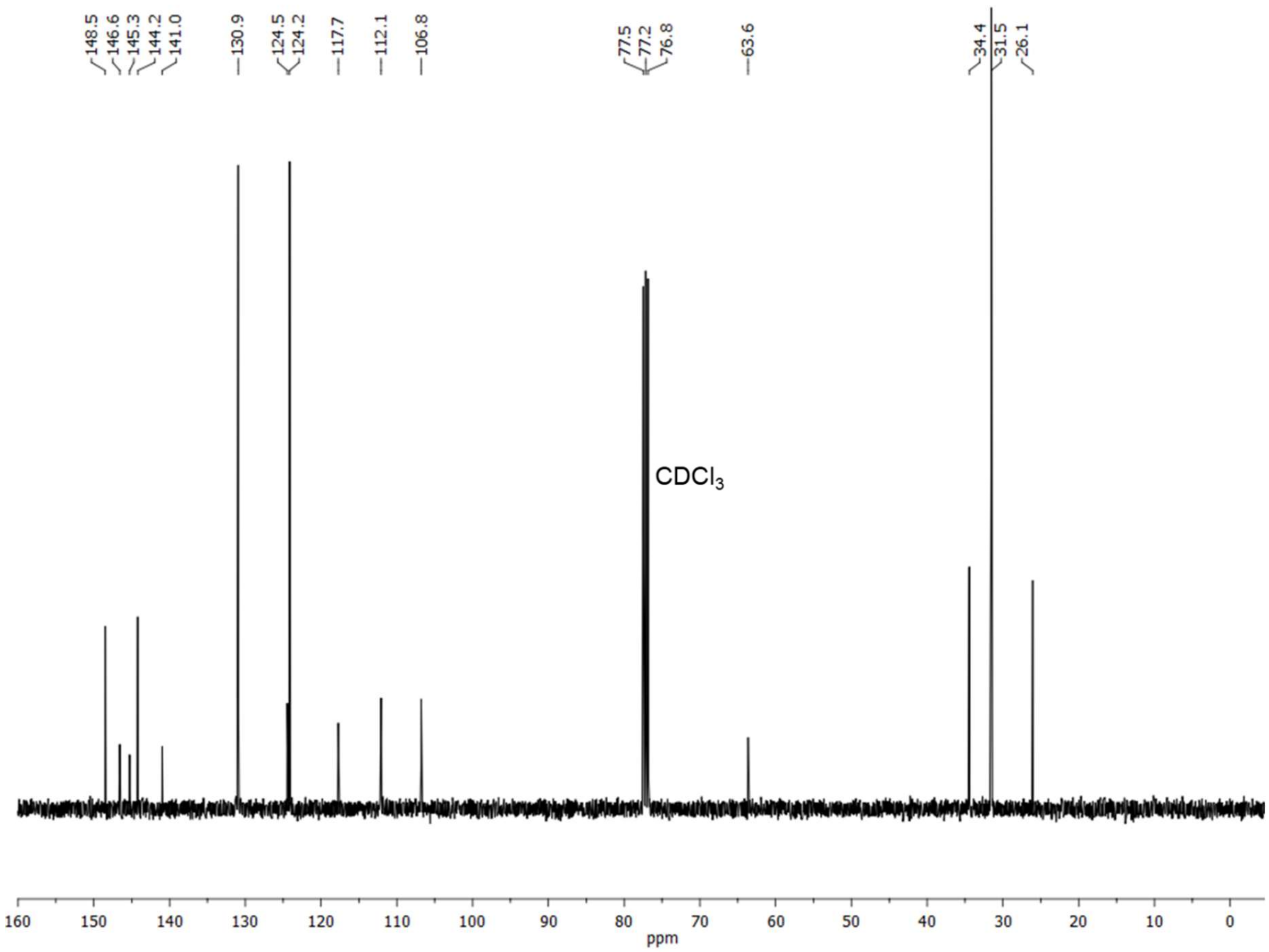

Fig. S37. $^{13}C\{^{1}H\}$ NMR spectrum (101 MHz, chloroform-*d*) of **Ace-3**.

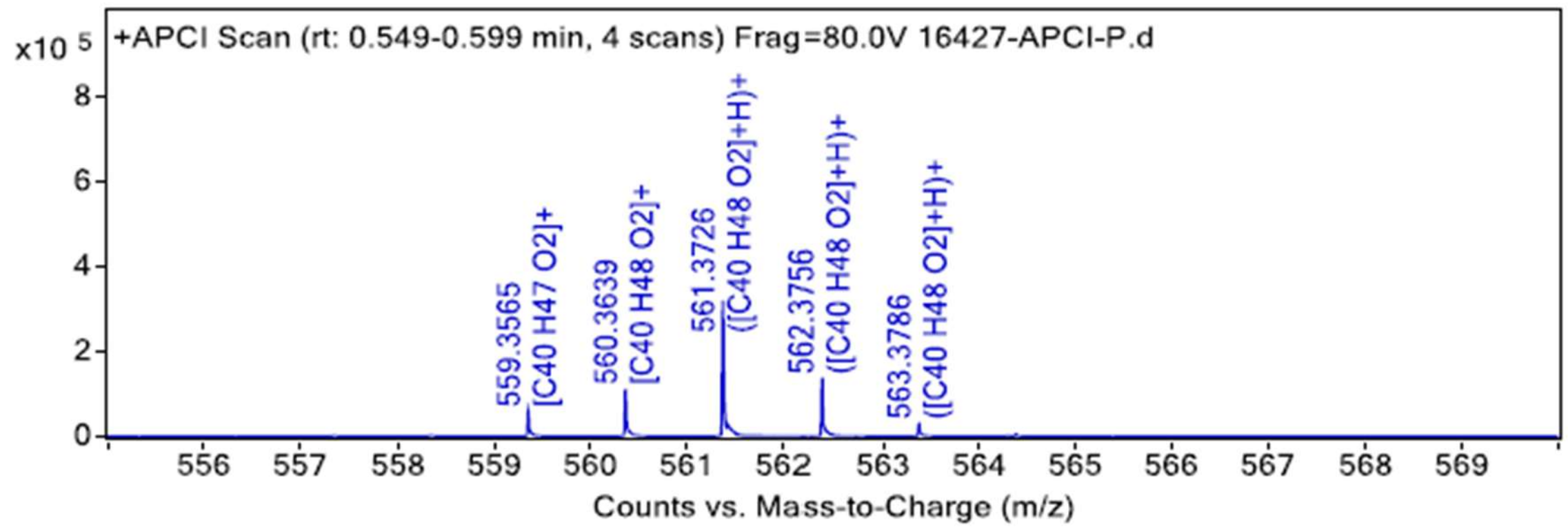

Fig. S38. High-resolution APCI-MS (HRMS) spectrum of **Ace-3**.

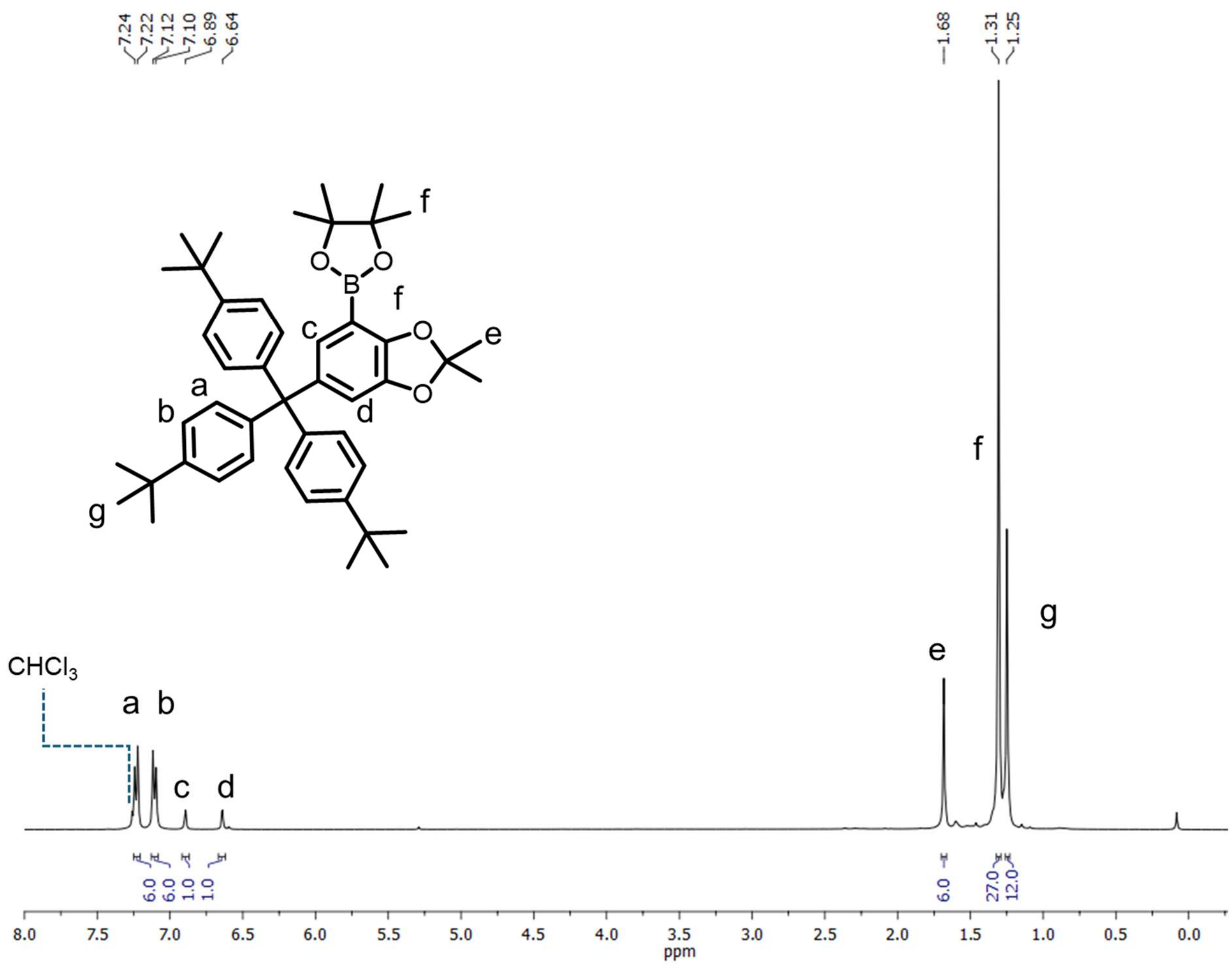

Fig. S39. ^{1}H NMR spectrum (400 MHz, chloroform-*d*) of **AceBpin-3**.

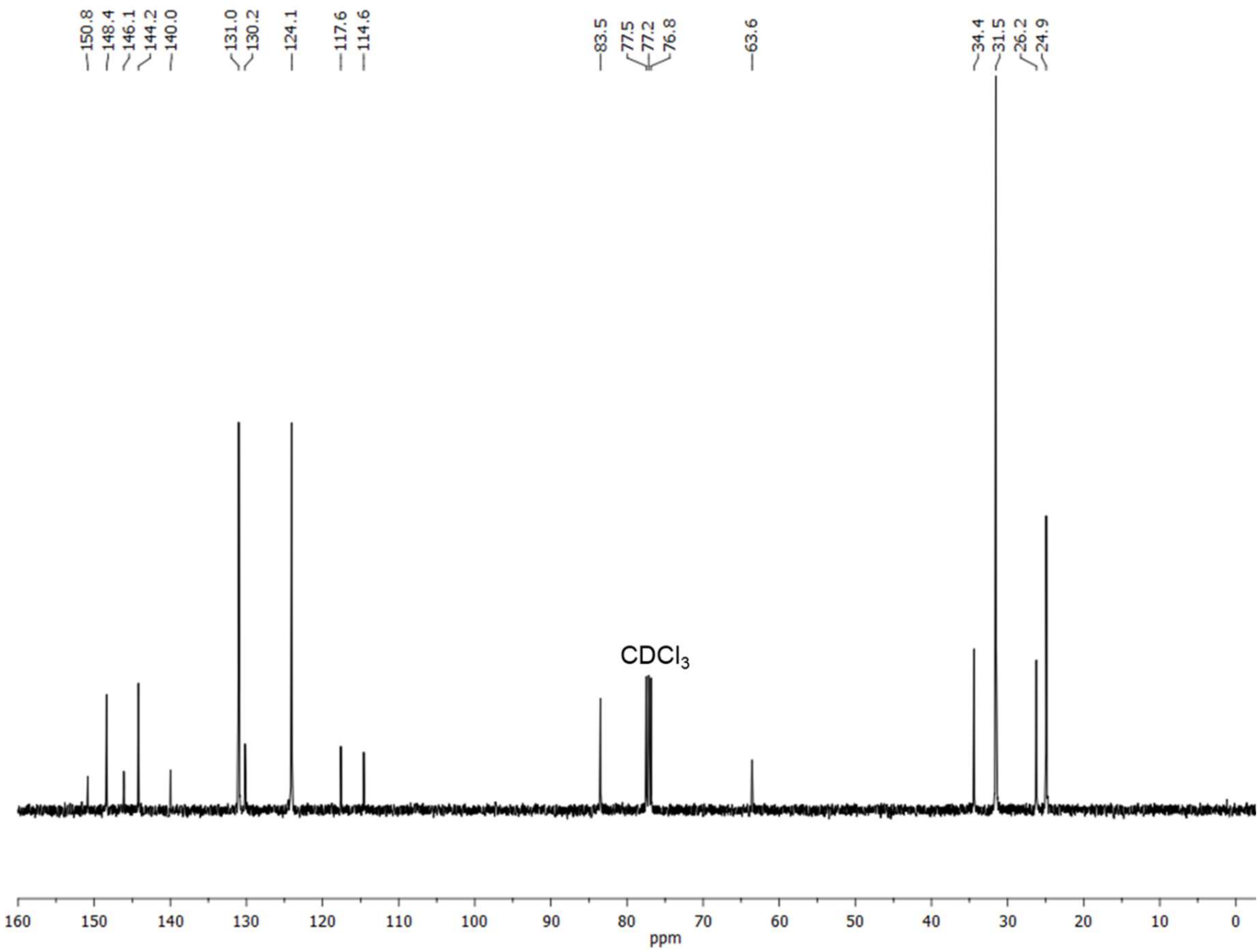

Fig. S40. $^{13}C\{^1H\}$ NMR spectrum (101 MHz, chloroform-*d*) of **AceBpin-3**.

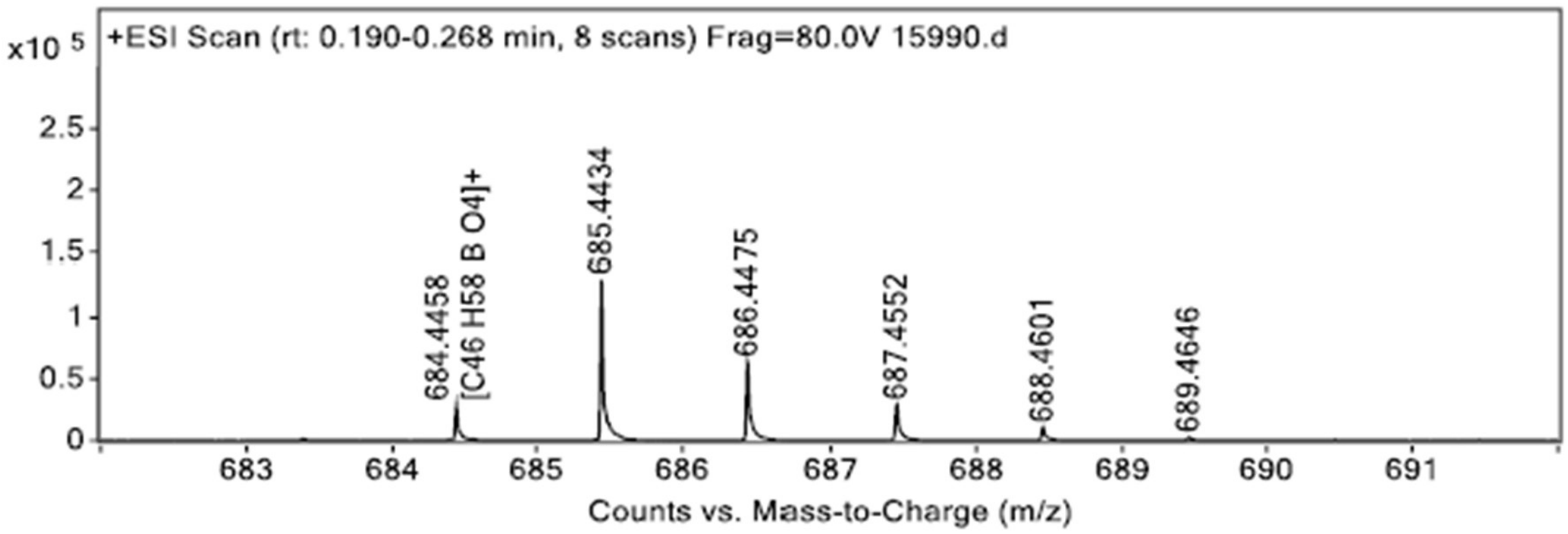

Fig. S41. High-resolution ESI-MS (HRMS) spectrum of **AceBpin-3**.

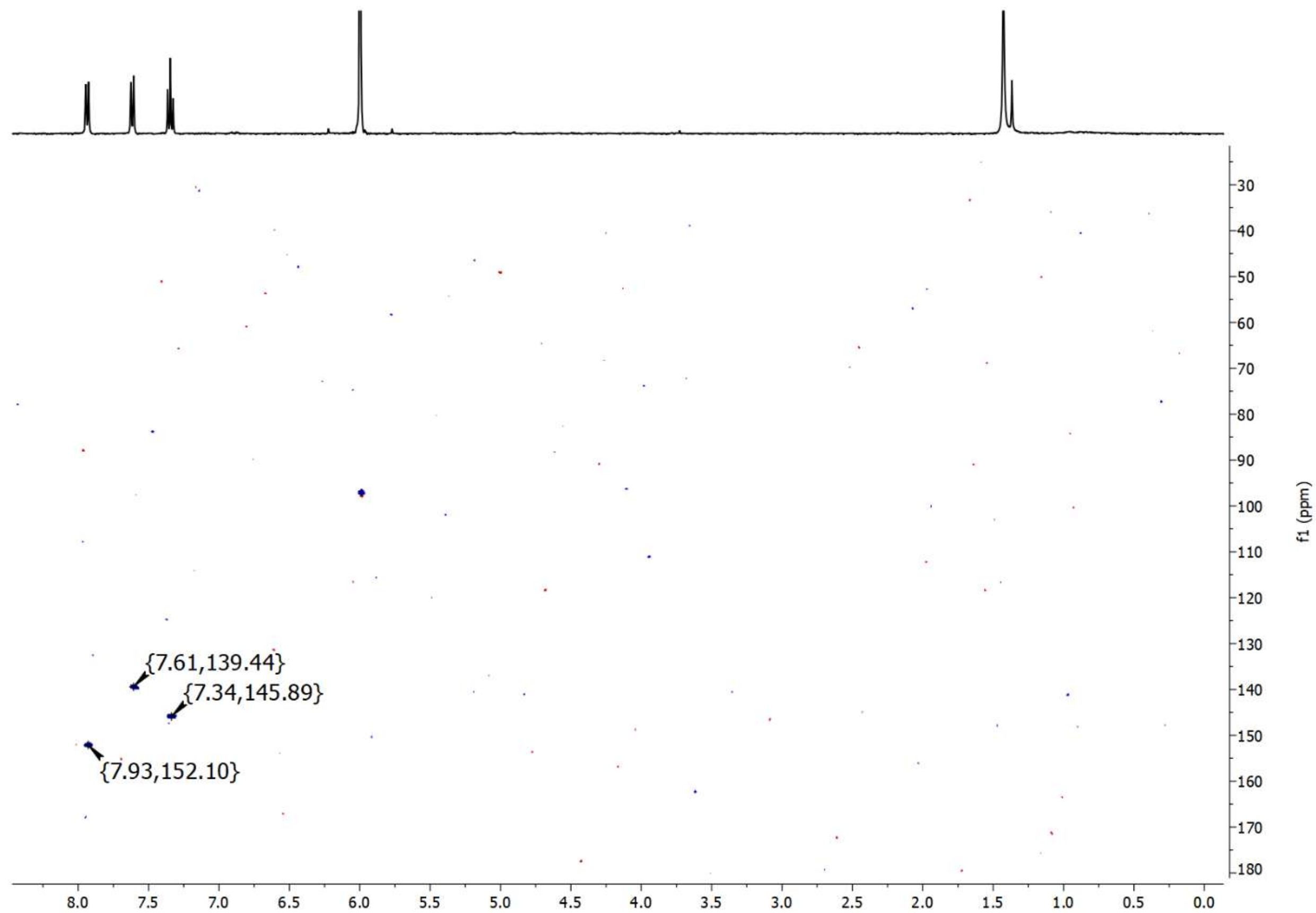

Fig. S42. 2D-HSQC NMR spectrum (400 MHz, 1,1,2,2,-tetrachloroethane-d_2) of **HBorph** at 120 °C.

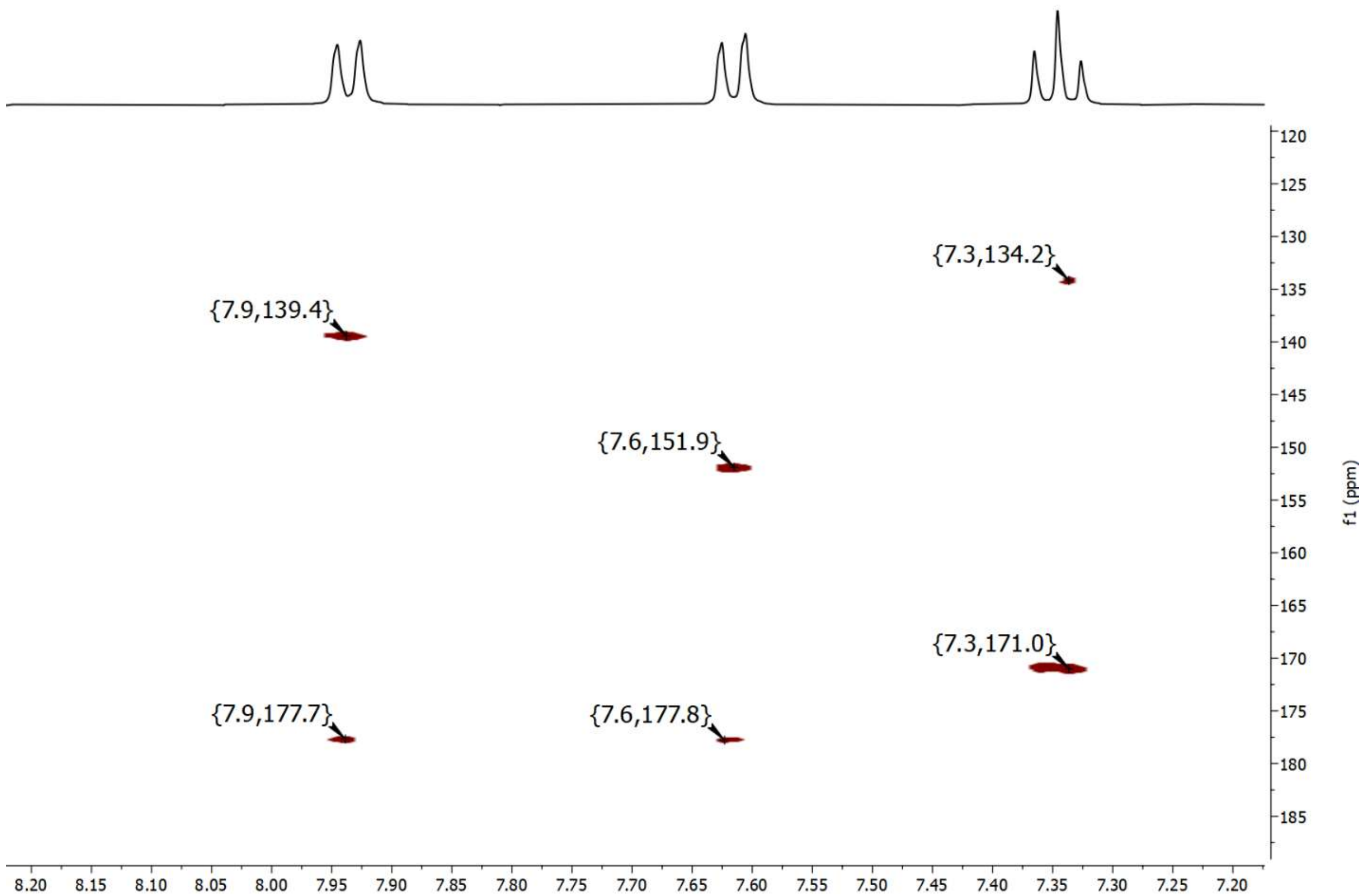

Fig. S43. 2D-HMBC NMR spectrum (400 MHz, 1,1,2,2,-tetrachloroethane-d_2) of **HBorph** at 120 °C.

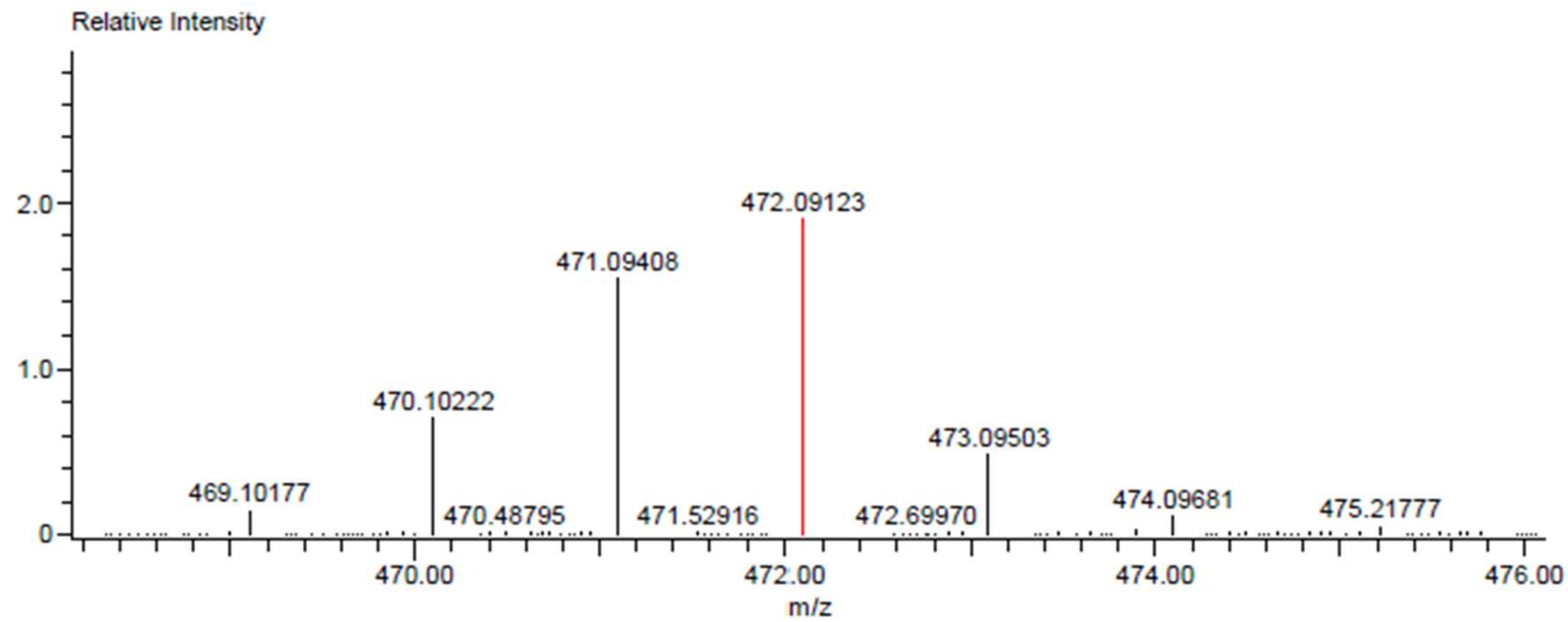

Fig. S44. High-resolution FD-MS (HRMS) spectrum of **HBorph**.

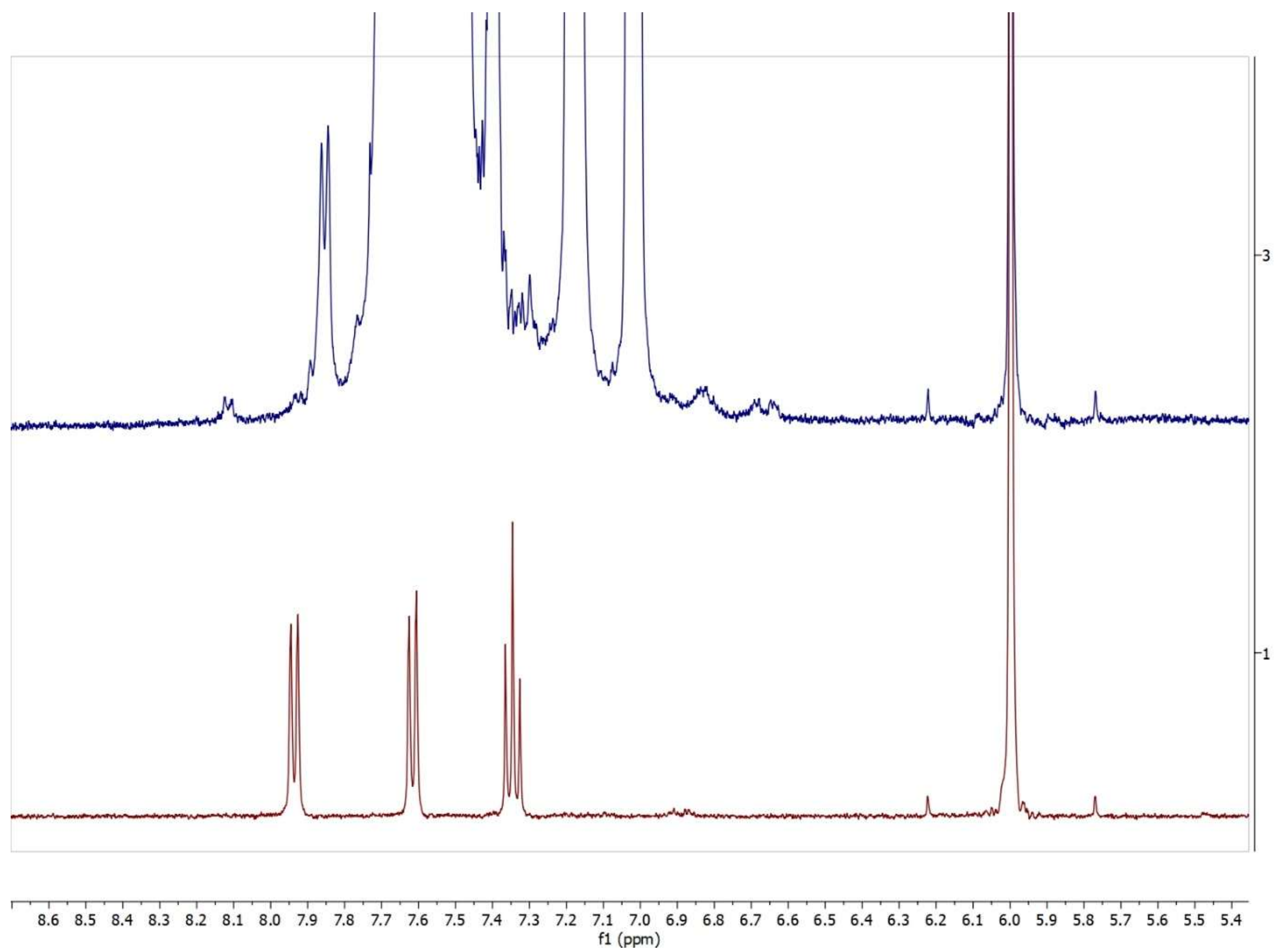

Fig. S45. ^{1}H NMR spectrum (400 MHz, 1,1,2,2,-tetrachloroethane-d_2) of **HBorph** (bottom) and **HBorph** with $LiBPh_4$ at 120 °C.

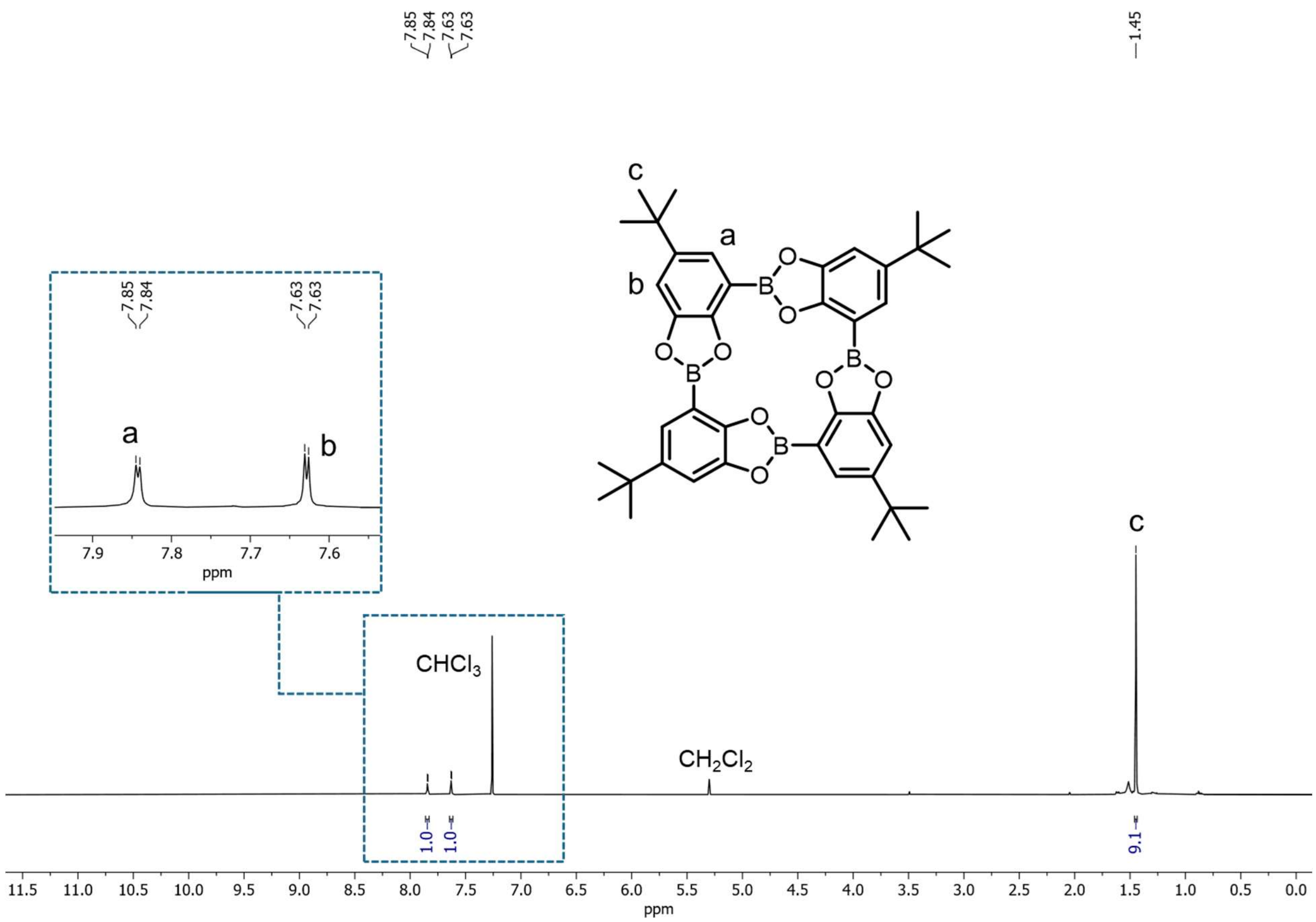

Fig. S46. ^{1}H NMR spectrum (400 MHz, chloroform-*d*) of **tBuBorph**.

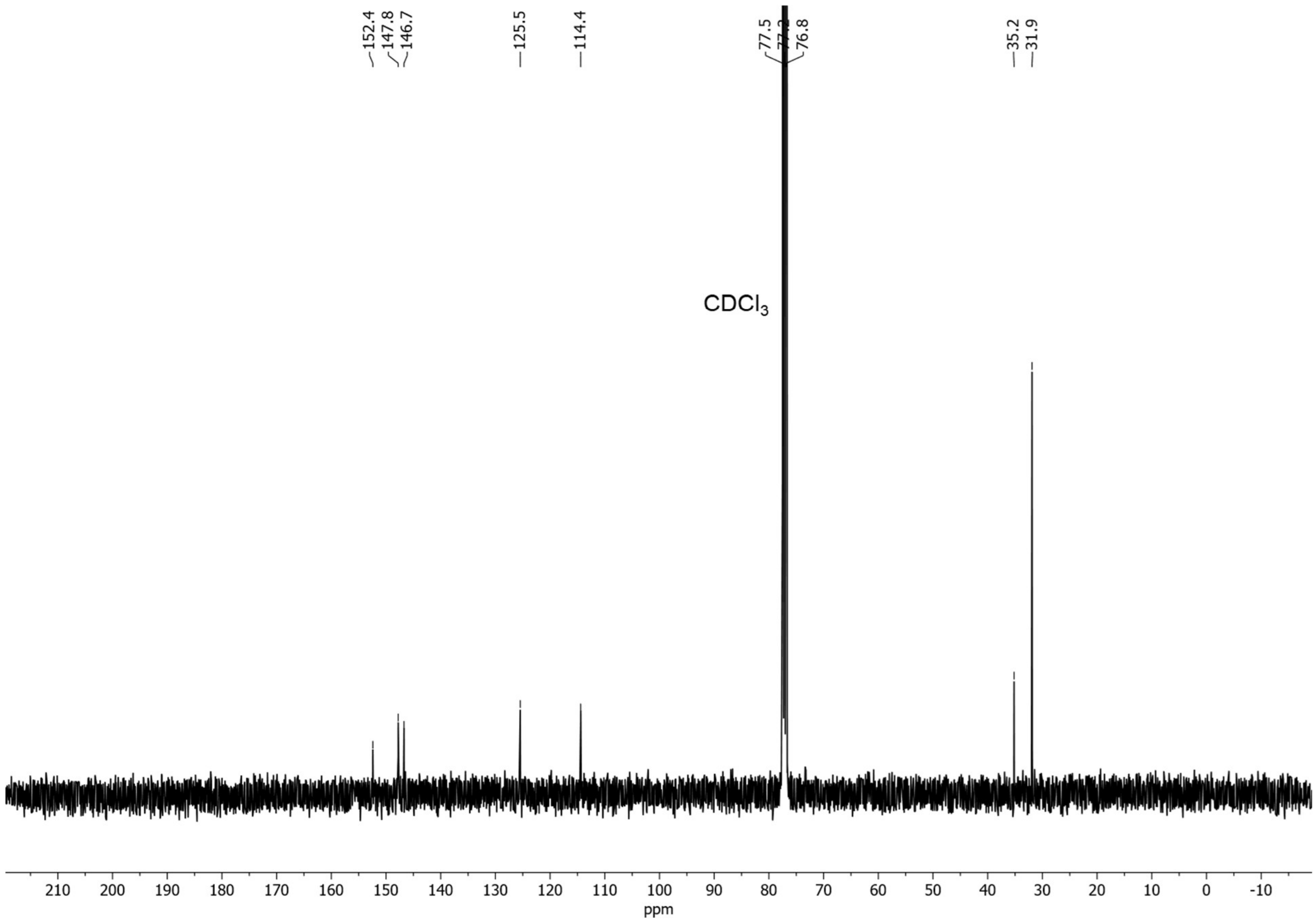

Fig. S47. $^{13}C\{^{1}H\}$ NMR spectrum (101 MHz, chloroform-*d*) of **tBuBorph**.

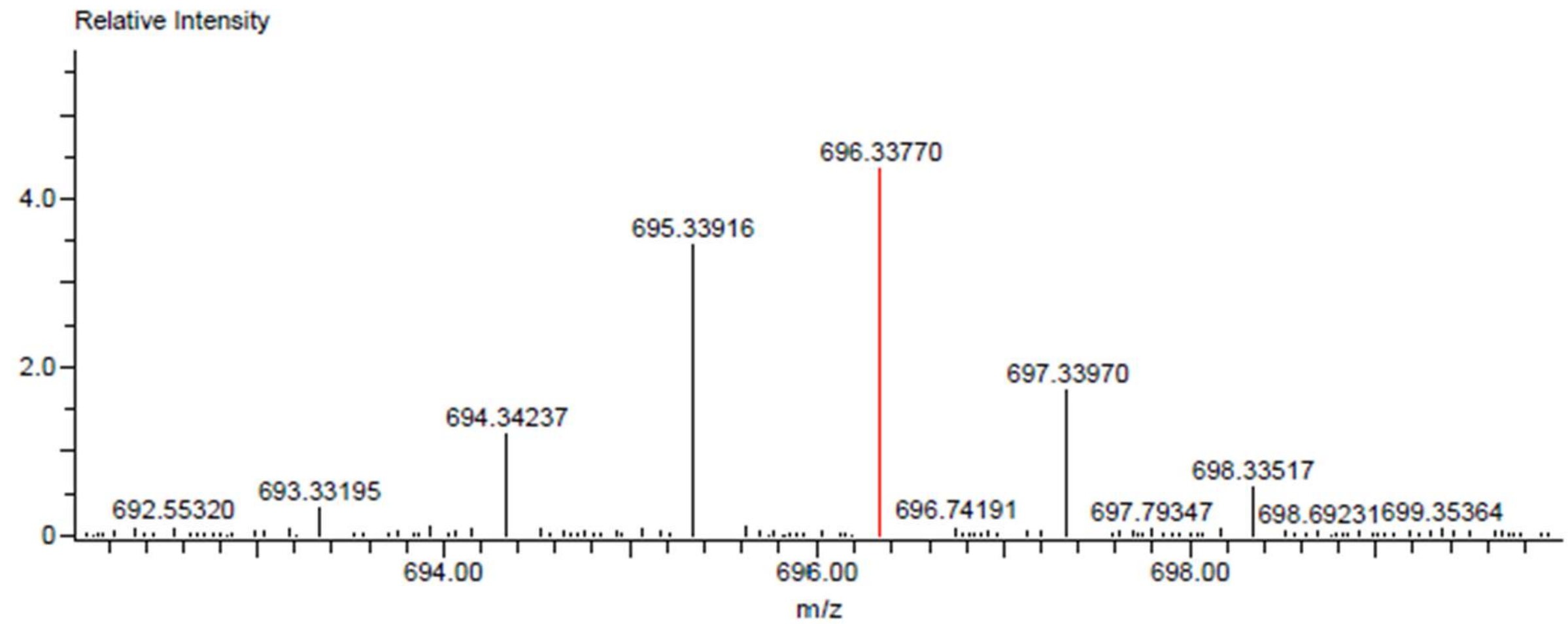

Fig. S48. High-resolution FD-MS (HRMS) spectrum of **tBuBorph**.

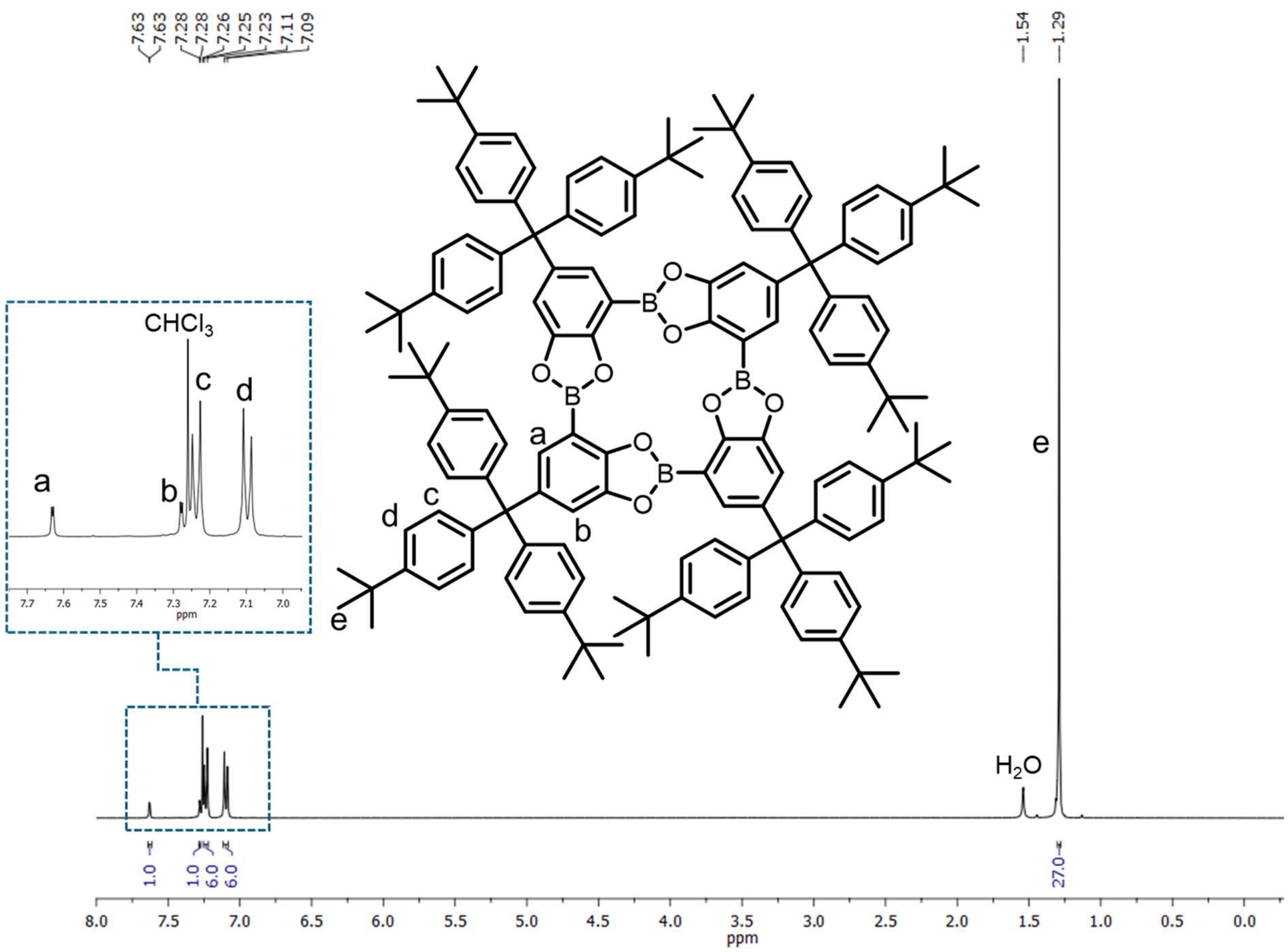

Fig. S49. ^{1}H NMR spectrum (400 MHz, chloroform-*d*) of **STBorph**.

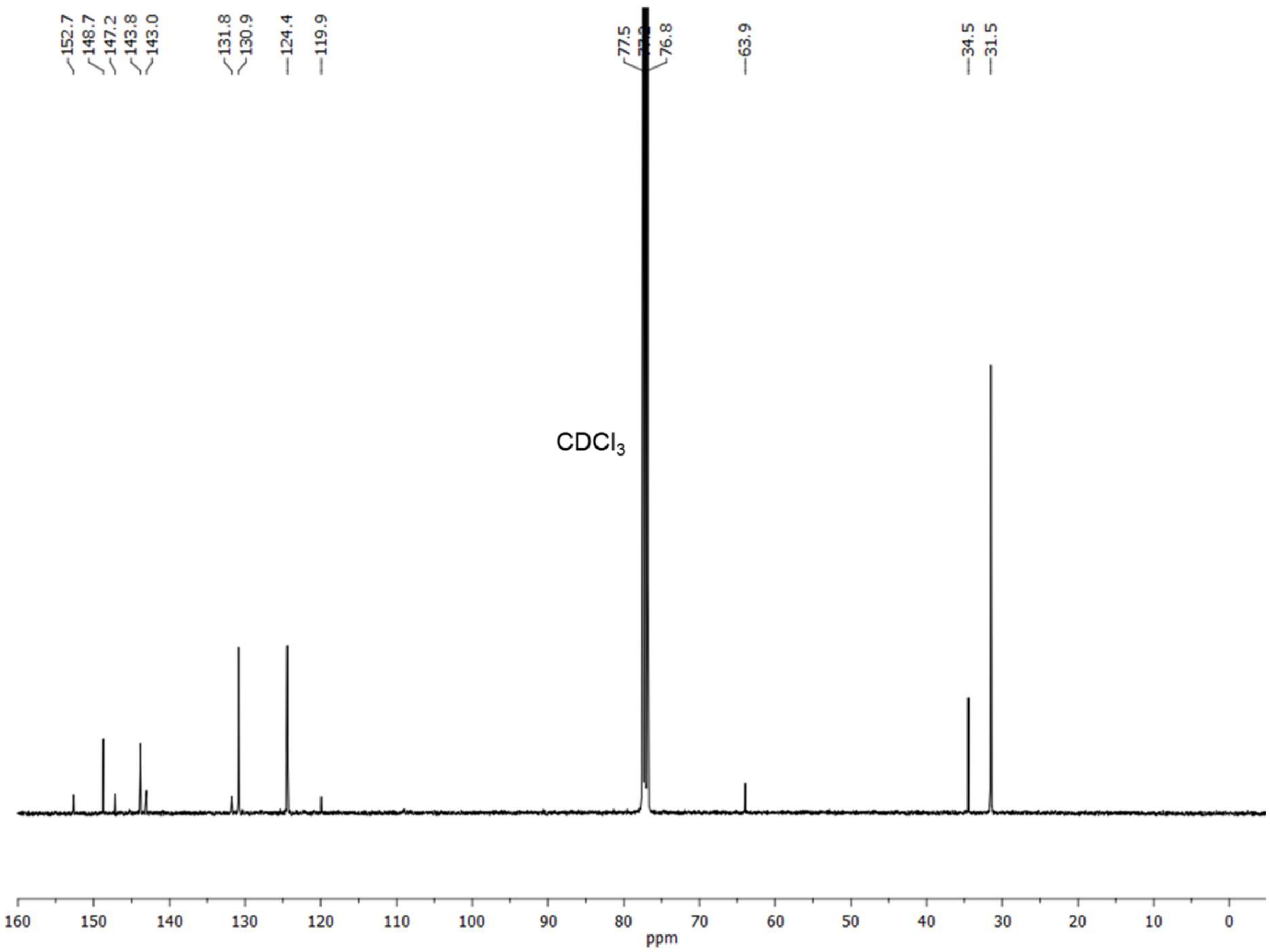

Fig. S50. $^{13}C\{^{1}H\}$ NMR spectrum (101 MHz, chloroform-*d*) of **STBorph**.

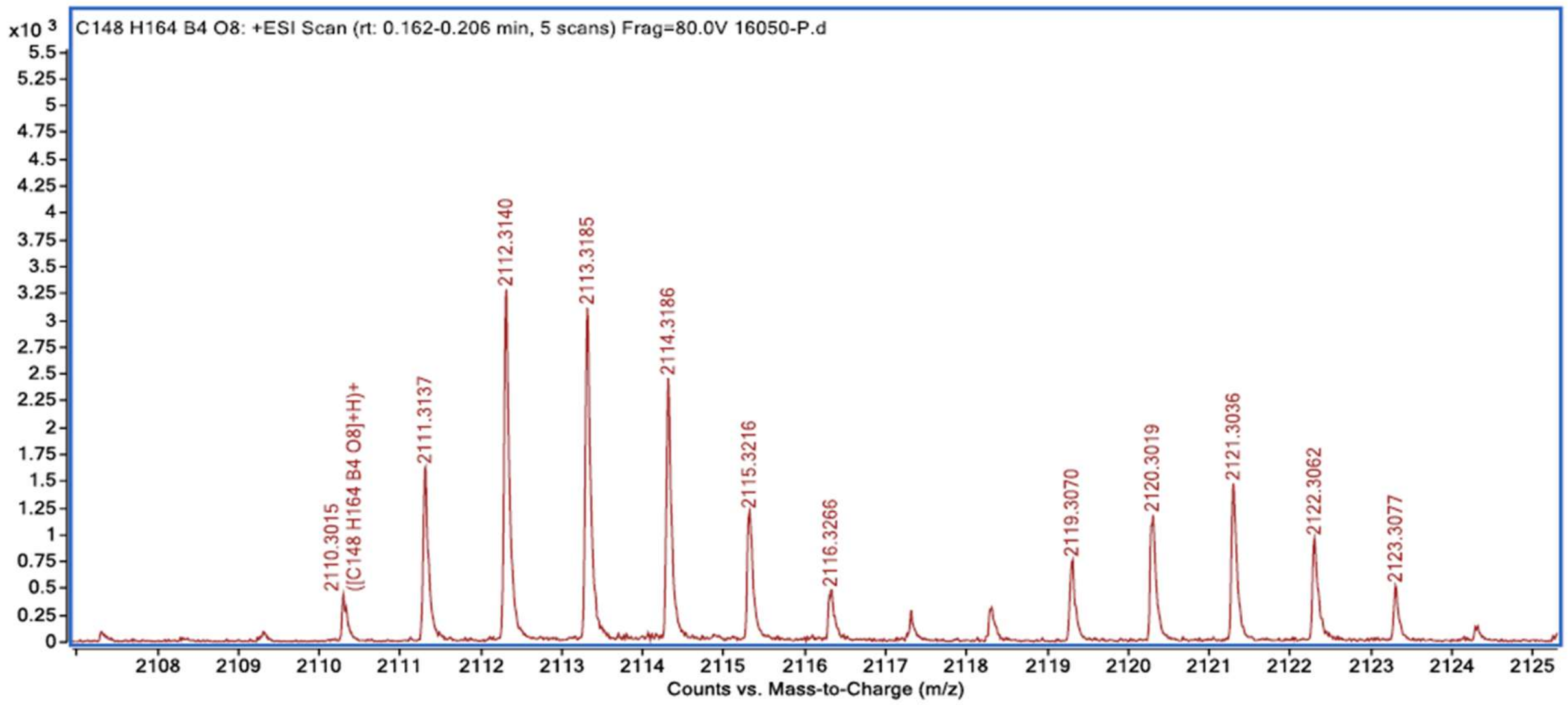

Fig. S51. High-resolution ESI-MS (HRMS) spectrum of $[C_{148}H_{164}B_4O_8]^+$ and $[C_{148}H_{164}B_4O_8Li]^+$.

References

1. Prevot-Halter, I. & Weiss, J. Synthetic receptors for dicarboxylates and diammoniums: templated assembly of functionalized catechols around molybdenum and complexation studies. *New J. Chem.* **22**, 869–874 (1998).

2. Gaussian 16, Revision B.01, Frisch, M.J., Trucks, G.W., Schlegel, H.B., Scuseria, G.E., Robb, M.A., Cheeseman, J.R.; Scalmani, G.; Barone, V.; Petersson, G.A.; Nakatsuji, H.; Li, X.; Caricato, M.; Marenich, A.V.; Bloino, J., Janesko, B.G., Gomperts, R., Mennucci, B., Hratchian, H.P., Ortiz, J.V., Izmaylov, A.F., Sonnenberg, J.L., Williams-Young, D., Ding, F., Lipparini, F., Egidi, F., Goings, J., Peng, B., Petrone, A., Henderson, T., Ranasinghe, D., Zakrzewski, V.G., Gao, J., Rega, N., Zheng, G., Liang, W., Hada, M.,

Ehara, M., Toyota, K., Fukuda, R., Hasegawa, J., Ishida, M., Nakajima, T., Honda, Y., Kitao, O., Nakai, H., Vreven, T., Throssell, K., Montgomery Jr., J.A., Peralta, J.E., Ogliaro, F., Bearpark, M.J., Heyd, J.J., Brothers, E.N., Kudin, K.N., Staroverov, V.N., Keith, T.A., Kobayashi, R., Normand, J., Raghavachari, K., Rendell, A.P., Burant, J.C., Iyengar, S.S., Tomasi, J., Cossi, M., Millam, J.M., Klene, M., Adamo, C., Cammi, R., Ochterski, J.W., Martin, R.L., Morokuma, K., Farkas, O., Foresman, J.B., Fox, D.J. Gaussian, Inc., Wallingford CT (2016) GaussView 5.0. Wallingford, E.U.A. - References - Scientific Research Publishing. https://scirp.org/reference/referencespapers?referenceid=2418053.

3. Francl, M. M., Pietro, W. J., Hehre, W. J., Binkley, J. S., Gordon, M. S., DeFrees, D. J. & Pople, J. A. Self-consistent molecular orbital methods. XXIII. A polarization-type basis set for second-row elements. *J. Chem. Phys.* **77**, 3654–3665 (1982).

4. Hay, P. J. & Wadt, W. R. Ab initio effective core potentials for molecular calculations. Potentials for K to Au including the outermost core orbitals. *J. Chem. Phys.* **82**, 299–310 (1985).

5. Wang, Z. Visualization of Aromaticity: AICD. https://wongzit.github.io/visualization-of-aromaticity-aicd/.

6. Geuenich, D., Hess, K., Köhler, F. & Herges, R. Anisotropy of the Induced Current Density (ACID), a General Method To Quantify and Visualize Electronic Delocalization. *Chem. Rev.* **105**, 3758–3772 (2005).

7. Iwagami, H., Yatagai, M., Nakazawa, M., Orita, H., Honda, Y., Ohnuki, T. & Yukawa, T. Synthesis of a Chiral α-(Aminooxy)arylacetic Ester. II. A Route through a Chiral 2-Hydroxy-2-phenylacetic Acid Derivative. *Bull. Chem. Soc. Jap.* **64**, 175–182 (1991).

8. Fukui, M., Mori, T., Inoue, Y. & Rathore, R. A New Class of Chiroptical Molecular Switches Based on the Redox-Induced Conformational Changes. *Org. Lett.* **9**, 3977–3980 (2007).

9. Gibson, H. W., Lee, S. H., Engen, P. T., Lecavalier, P., Sze, J., Shen, Y. X. & Bheda, M. New triarylmethyl derivatives: "blocking groups" for rotaxanes and polyrotaxanes. *J. Org. Chem.* **58**, 3748–3756 (1993).

10. Cram, D. J. & Lein, G. M. Host-guest complexation. 36. Spherand and lithium and sodium ion complexation rates and equilibria. *J. Am. Chem. Soc.* **107**, 3657–3668 (1985).

11. Guo, X., Yang, Y., Peng, Z., Cai, Y., Feng, W. & Yuan, L. Highly efficient synthesis of hydrogen-bonded aromatic tetramers as macrocyclic receptors for selective recognition of lithium ions. *Org. Chem. Front.* **6**, 2654–2661 (2019).

12. Chen, J.-A., Lai, J.-L., Lee, G. H., Wang, Y., Su, J. K., Yeh, H.-C., Lin, W.-Y. & Leung, M. Cooperative and Selective Lithium Complexation of 2,11,13,22-Tetraaza-5,8,16,19-tetraoxa-1,12-dioxocyclodocosanes. *Org. Lett.* **3**, 3999–4002 (2001).

13. Wang, H., Jones, L. O., Hwang, I., Allen, M. J., Tao, D., Lynch, V. M., Freeman, B. D., Khashab, N. M., Schatz, G. C., Page, Z. A. & Sessler, J. L. Selective Separation of Lithium Chloride by Organogels Containing Strapped Calix[4]pyrroles. *J. Am. Chem. Soc.* **143**, 20403–20410 (2021).

14. Grote, Z., Lehaire, M.-L., Scopelliti, R. & Severin, K. Severin, Selective Complexation of Li^+ in Water at Neutral pH Using a Self-Assembled Ionophore. *J. Am. Chem. Soc.* **125**, 13638–13639 (2003).

15. Gohil, H., Chatterjee, S., Yadav, S., Suresh, E. & Paital, A. R. An Ionophore for High Lithium Loading and Selective Capture from Brine. *Inorg. Chem.* **58**, 7209–7219 (2019).

16. Luo, Y., Marets, N. & Kato, T. Selective lithium ion recognition in self-assembled columnar liquid crystals based on a lithium receptor. *Chem. Sci.* **9**, 608–616 (2018).

17. Kim, H. & Koo, B. Lithium sensors based on photophysical changes of 1-aza-12-crown-4 naphthalene derivatives synthesized via Buchwald–Hartwig amination. *RSC Adv.* **12**, 31976–31984 (2022).

18. Gunnlaugsson, T., Bichell, B. & Nolan, C. A novel fluorescent photoinduced electron transfer (PET) sensor for lithium. *Tetrahedron Lett.* **43**, 4989–4992 (2002).

19. Kumar, M., Kumar, N. & Bhalla, V. Ratiometric detection of Hg^{2+} ions: an allosterically synchronized Hg^{2+}/Li^+ switch based on thiacalix[4]crown. *Dalton Trans.* **40**, 5170–5175 (2011).

20. Oral, I. & Abetz, V. A Highly Selective Polymer Material using Benzo-9-Crown-3 for the Extraction of Lithium in Presence of Other Interfering Alkali Metal Ions. *Macromol. Rapid Commun.* **42**, 2000746 (2021).

21. He, Q., Williams, N. J., Oh, J. H., Lynch, V. M., Kim, S. K., Moyer, B. A. & Sessler, J. L. Selective Solid–Liquid and Liquid–Liquid Extraction of Lithium Chloride Using Strapped Calix[4]pyrroles. *Angew. Chem. Int. Ed.* **57**, 11924–11928 (2018).

22. Bourhis, L. J., Dolomanov, O. V., Gildea, R. J., Howard, J. A. K. & Puschmann, H. The anatomy of a comprehensive constrained, restrained refinement program for the modern computing environment – Olex2 dissected. *Acta Cryst. A* **71**, 59–75 (2015).

23. Dolomanov, O. V., Bourhis, L. J., Gildea, R. J., Howard, J. A. K. & Puschmann, H. OLEX2: a complete structure solution, refinement and analysis program. *J Appl. Cryst.* **42**, 339–341 (2009).

24. Sheldrick, G. M. SHELXT – Integrated space-group and crystal-structure determination. *Acta Cryst. A* **71**, 3–8 (2015).

25. SAINT V8.40B, Bruker AXS, Madison, WI., 2022.

26. SADABS-2016/2, Bruker AXS, Madison, WI., 2016.
