# Supplementary material for: Head-to-tail assembled borphyrins for solid-state lithium extraction
Source: Nat Commun. 2026 Oct 1;17:11270. doi: 10.1038/s41467-026-78080-0 (PMC13720209; doi:10.1038/s41467-026-78080-0)
Supplement: Supplementary file 2 [file 41467_2026_78080_MOESM2_ESM.pdf]

nature portfolio

Peer Review File

Head-to-tail assembled borphyrins for solid-state lithium extraction

Corresponding Author: Professor Mark MacLachlan

This file contains all reviewer reports in order by version, followed by all author rebuttals in order by version.

Version 0:

Reviewer comments:

Reviewer #1

(Remarks to the Author)
This manuscript reports a new class of rigid, planar dioxaborole-linked macrocycles ("borphyrins") that exhibit strong and selective lithium binding. The authors demonstrate that these macrocycles can extract Li^+ from various salts, including low-solubility salts, and from complex mixtures mimicking seawater, highlighting the potential for selective lithium recovery.

Lithium extraction is acutually a long-studied problem, but recieved more attentions in recent years because of Li-battery. Decades ago, Cram and co-workers developed spherands that already exhibited very high lithium affinity and selectivity (the authors should cite theirs work). A recent review (Coordination Chemistry Reviews 515 (2024): 215968) also summarizes many relevant approaches (the authors should cite this as well). While the molecular design in this manuscript and the reported high Li^+ affinity and selectivity are impressive, major revision is needed to justify publication in Nature Communications.

1. Although the rigid receptor and size-matched cavity likely contribute to high specificity, the actual Li^+/Na^+ affinity difference is not quantitatively established. The manuscript shows UV–vis spectra indicating minimal changes upon addition of Na^+, but no binding constants are reported. This reviewer finds it difficult to imagine that the system has virtually no Na^+ binding at all, because even if a 1:1 host–guest complex cannot form with Na^+, a 2:1 binding mode could still be possible, as observed in macrocyclic anion receptors by Amar Flood and co-workers. Even if the actual $K_a(Na^+)$ is below 10 M^{-1}, this would itself be a very impressive and intuitive result. Direct NMR titrations (direct or competitive titrations) could be used to obtain quantitative values, and providing such data would give a much clearer picture of the receptor's selectivity.

2. While the authors report single crystals of two macrocycles, structural characterization of the Li^+@tBuBorph complex would provide stronger evidence for Li^+ binding and help validate the proposed binding mode.

3. The authors assert that their system offers practical advantages over previous macrocycles: "These systems often require multistep low-yield synthesis, show weak lithium binding affinity, poor selectivity, or dependence on specific salts for effective extraction in either solid-state or solution environments, which have further limited practical applications." However, this manuscript does not provide sufficient evidence to support a clear superiority in terms of practical applications. To overcome solubility limitations, bulky substituents were introduced, reducing the simplicity of the system. Moreover, boronic esters have inherent stability limitations, particularly in aqueous or extreme pH environments. Without additional data demonstrating that borphyrins are truly more suitable for practical applications than existing systems, the critique of previous work appears overstated.

4. Regarding potential applications, the "sea salt mimic" extraction is carried out by drying the salts and performing extraction in cyclopentyl methyl ether. This approach differs significantly from direct aqueous-phase extraction and requires additional processing steps, including concentration, solvent handling, and eventual recovery of the receptor. The manuscript should explicitly note these limitations to avoid giving a misleading impression of practical applicability.

5. To better illustrate the potential utility of the system, the authors could consider a continuous extraction setup, such as a U-tube or membrane-based experiment, to evaluate whether selective Li^+ transfer from an aqueous or mixed-phase solution can be achieved. Such studies would provide valuable insight into the real-world applicability of borphyrins.

6. Even as a non-specialist in the field, this reviewer noticed that some relevant literature may be missing. The authors should ensure that prior high-affinity Li^+ receptors and recent reviews are properly cited, to place this work in a more complete context.

Reviewer #2

(Remarks to the Author)
This manuscript reports a new class of macrocycles, termed borphyrins, featuring a dioxaborole-linked head-to-tail architecture. The synthetic strategy is elegant, operationally simple, and effective, relying on the condensation of a protected boric acid derivative to circumvent protodeborylation. All three macrocycles are rigorously characterized by single-crystal X-ray diffraction. Structurally, these compounds define a rigid cavity reminiscent of a 16-crown-4 motif and exhibit high affinity and selectivity for lithium ions in organic solvents, enabling extraction of lithium from crystalline solids or mixed salt systems into organic phases. Despite the aesthetic appeal of these molecules, several concerns limit the overall impact of the work. The key points are outlined below:
1. The Introduction emphasizes the importance of lithium extraction from brines or seawater; however, the study focuses exclusively on solid–liquid extraction, which is less directly aligned with this motivation. Moreover, the reported macrocycles are not compatible with aqueous environments and therefore cannot be applied to lithium recovery from water. The authors should revise the Introduction to more accurately frame the significance of solid–liquid extraction, clearly identify the outstanding challenges in this area, and articulate how the present work addresses a specific gap.
2. A major limitation of these macrocycles for lithium separation is their susceptibility to protodeborylation under aqueous conditions. This instability restricts their application to lithium capture without a viable pathway for controlled release and recyclability. Given that practical separation technologies require reversible binding, this issue represents a critical weakness. Demonstration of a strategy for receptor regeneration and lithium release would be essential to substantiate the broader applicability of this system. In its current form, this limitation raises concerns regarding the suitability of the work for publication in a high-impact journal such as Nature Communications.
3. For improved clarity and reproducibility, the authors should explicitly report the receptor concentrations used in both the NMR titration experiments (Figure 2C) and the liquid–solid extraction studies.

Reviewer #3

(Remarks to the Author)
The manuscript reports the synthesis of a new class of rigid dioxaborole-linked macrocycles (“borphyrins”) obtained through a head-to-tail assembly strategy and demonstrates their highly selective Li+ binding and extraction behaviour. The synthetic concept is clearly original, and the combination of rigid cavity design, strong lithium affinity, and extraction from complex salt mixtures will likely be of interest to the supramolecular and separation chemistry communities. In particular, the scalable synthesis and unusual macrocyclic architecture represent clear strengths of the work.
At the same time, several aspects of the study require further clarification before publication in Nat. Commun. While the supramolcular chemistry itself is convincing I find some of the broader claims regarding sustainable Li+ extraction and practical applicability somewhat overstated based on the current experimental evidence. I therefore recommend major revision!

My major comments and concerns are:

1) Structural evidence for Li+ encapsulation
A central claim of the manuscript is the selective encapsulation of Li+ within the rigid borphyrin cavity. However, despite extensive crystallographic characterization of the free borphyrin, no single-crystal X-ray structure of a Li+-bound complex is presented. The proposed binding mode is currently supported mainly by DFT calculations, UV/Vis titrations, NMR spectroscopy, and mass spectrometry.
I appreciate that crystallization of Li+ complexes can be experimentally difficult and that Li+ itself is only weakly scattering. Nevertheless, for a study in which the proposed cavity-controlled binding mode represents such a central aspect, the absence of direct structural evidence is noticeable. The authors should either provide a crystallographically characterized Li complex or discuss more explicitly why such a structure could not be obtained. Where possible, the experimental support for the proposed binding model should be strengthened further.

2) Stability and recyclability of the extraction system
The manuscript presents borphyrins as promising systems for sustainable lithium recovery. However, the authors also state that the borphyrin undergoes protodeborylation during lithium recovery, preventing recyclability of the investigated systems. In my opinion, this represents a significant limitation with respect to practical implementation and should be discussed more critically throughout the manuscript.
At present, the study still reads more as a proof-of-concept supramolecular extraction system than a practically viable recovery platform.

3) Mechanistic understanding of Mg^{2+} interference
The decrease in extraction efficiency observed in the presence of MgSO4 and CaSO4 is mainly attributed to desiccant effects. However, this explanation currently remains rather speculative and is not sufficiently supported experimentally. This point is particularly important because Mg/Li separation is one of the central challenges in lithium recovery from brines and seawater. A more detailed mechanistic discussion would therefore strengthen the manuscript considerably. Additional

control experiments or discussion addressing possible competitive coordination, ion pairing, precipitation effects, or solvent-dependent behaviour would help clarify this issue.

4) Scope of the application-oriented claims
The chemistry is interesting and the selectivity results are certainly noteworthy. However, some statements regarding sustainable lithium recovery and practical extraction applications appear somewhat ahead of the demonstrated robustness of the system. In particular, the absence of recyclability and the still relatively preliminary extraction studies suggest that the practical implications should be discussed more cautiously.

Overall, I find the chemistry original and scientifically interesting, and I believe the work could become suitable for Nat. Commun. after substantial revision. However, in its current form I am not yet fully convinced that the mechanistic and structural evidence supporting the central claims reaches the level expected for publication in the journal. In particular the lack of direct strutural validation of the Li-bound state and the still limited mechanistic understanding of the extraction behaviour under competitive conditions weaken the broader application-oriented conclusions.

Version 1:

Reviewer comments:

Reviewer #1

(Remarks to the Author)
The revision has addressed several technical concerns by providing additional Na^+ binding data, clarifying the extraction conditions, and moderating the application-oriented claims. These revisions improve the rigor and accuracy of the manuscript. However, they do not substantially change my original assessment regarding the overall significance of the work.

Importantly, the original manuscript was motivated and positioned from a practical lithium extraction perspective. In the revised version, however, the authors have explicitly acknowledged that the present system should only be regarded as a proof-of-concept molecular recognition platform rather than a practically relevant lithium recovery technology. This substantially changes the significance of the work.

Once the practical applicability is removed from the central claim, the manuscript is essentially reduced to the development of a new Li^+-binding macrocycle. In this context, the key question becomes what conceptual or functional advance this receptor provides over established high-affinity Li^+ receptors, particularly classical spherands. Although the borphyrin architecture is structurally elegant and synthetically interesting, I do not believe the current manuscript demonstrates a sufficiently significant conceptual advance in molecular recognition. The revised manuscript also explicitly acknowledges important limitations of the present system, including poor hydrolytic stability, lack of recyclability, and the inability to perform continuous or aqueous extraction.

Therefore, unless these fundamental limitations can be addressed, I believe this work remains at the level of introducing a new Li^+-binding macrocycle rather than establishing a genuinely impactful advance in lithium recognition or separation. While the chemistry is interesting, I am still not convinced that the conceptual novelty and overall significance reach the level expected for publication in Nature Communications.

Reviewer #2

(Remarks to the Author)
The authors have adequately addressed my previous comments by moderating their claims and more clearly acknowledging the current limitations of their receptors. I no longer have any objections to the publication of this manuscript and recommend its acceptance.

Reviewer #3

(Remarks to the Author)
The authors have carried out a comprehensive revision and responded constructively to the major concerns I raised in my previous review. In particular, the extensive crystallization experiments for Li+-bound borphyrin complexes are now documented, and the manuscript correctly notes that the proposed Li+-associated geometry is supported more by the combined spectroscopic, mass spectrometric, and computational evidence than by direct crystallographic validation. I consider this to be an appropriate and transparent handling of the structural limitation.

The lack of reusability is now also discussed much more clearly. The revised manuscript explicitly identifies protodeborylation during aqueous or protic Li+ release as a limitation of the present derivatives and appropriately presents the system as a “proof-of-concept” platform for molecular recognition rather than as an immediately applicable or sustainable technology for lithium recovery.

The origin of the reduced extraction efficiency in Mg2+- and Ca2+-containing mixtures remains mechanistically unclear. The absence of measurable co-extraction of these ions does not, in itself, rule out transient coordination or indirect interfacial and

solid-state effects. However, the revised manuscript no longer attributes the effect to a single mechanism and now appropriately presents it as a limitation of the extraction system under complex solid-salt conditions. I therefore do not consider additional mechanistic experiments to be essential for publication.

Two statements in the abstract must nevertheless be corrected. First, the description of the lithium affinity as “nanomolar” contradicts the reported association constants of 1.8×106 and 3.9×105 M-1, which correspond to dissociation constants of approximately 0.56 and 2.6 µM, respectively. The affinity should therefore be described as strong or as falling within the submicromolar to micromolar range, rather than as nanomolar.

Second, the statement that there is “no interference from overcompeting cations” is not supported by the extraction results, since the addition of alkaline earth salts reduces the extraction efficiency from approximately 98.6% to approximately 50%. Although these ions are not co-extracted to any significant extent, they clearly influence lithium extraction. This sentence should be revised to distinguish between selectivity and co-extraction based on the observed reduction in extraction efficiency.

Subject to the correction of these points, I consider the revised manuscript ready for publication.

Open Access This Peer Review File is licensed under a Creative Commons Attribution 4.0 International License, which permits use, sharing, adaptation, distribution and reproduction in any medium or format, as long as you give appropriate credit to the original author(s) and the source, provide a link to the Creative Commons license, and indicate if changes were made.
In cases where reviewers are anonymous, credit should be given to 'Anonymous Referee' and the source.
The images or other third party material in this Peer Review File are included in the article’s Creative Commons license, unless indicated otherwise in a credit line to the material. If material is not included in the article’s Creative Commons license and your intended use is not permitted by statutory regulation or exceeds the permitted use, you will need to obtain permission directly from the copyright holder.
To view a copy of this license, visit https://creativecommons.org/licenses/by/4.0/

Response to Reviewers

Dear Reviewers,

We thank you for the careful evaluation of our manuscript. We believe that the current manuscript has been strengthened by your constructive comments. A detailed response to your comments is given below (where the resulting changes to the MS and SI are underscored). To address your criticism, we have resynthesized new batches of STBorph and attempted 96 different crystallization experiments and performed additional measurements on lithium selectivity and lithium extractions as well as the sodium competitive binding. Other results of the new measurements are provided in/added to Figures S13A-C. We have also highlighted all changes to the manuscript.

Reviewer 1

This manuscript reports a new class of rigid, planar dioxaborole-linked macrocycles ("borphyrins") that exhibit strong and selective lithium binding. The authors demonstrate that these macrocycles can extract Li^+ from various salts, including low-solubility salts, and from complex mixtures mimicking seawater, highlighting the potential for selective lithium recovery.

Lithium extraction is acutually a long-studied problem, but recieved more attentions in recent years because of Li-battery. Decades ago, Cram and co-workers developed spherands that already exhibited very high lithium affinity and selectivity (the authors should cite theirs work). A recent review (Coordination Chemistry Reviews 515 (2024): 215968) also summarizes many relevant approaches (the authors should cite this as well). While the molecular design in this manuscript and the reported high Li^+ affinity and selectivity are impressive, major revision is needed to justify publication in Nature Communications.

Response: We thank the reviewer for the detailed evaluation of our work as well as their constructive comments and questions. Their remarks have indeed shed light on aspects of the work and helped reshape the introduction of the paper. We agree that lithium recognition and extraction are long-studied problems and that important precedents, including Cram's spherands, should be discussed more explicitly. In the revised manuscript, we have expanded the Introduction to include Cram and co-workers' seminal work on preorganized spherands and the recent Coordination Chemistry Reviews article on lithium-selective receptors (now Ref. 7-9).

1. Although the rigid receptor and size-matched cavity likely contribute to high specificity, the actual Li^+/Na^+ affinity difference is not quantitatively established. The manuscript shows UV–vis spectra indicating minimal changes upon addition of Na^+, but no binding constants are reported. This reviewer finds it difficult to imagine that the system has virtually no Na^+ binding at all, because even if a 1:1 host–guest complex cannot form with Na^+, a 2:1 binding mode could still be possible, as observed in macrocyclic anion receptors by Amar Flood and co-workers. Even if the actual $K_a(Na^+)$ is below 10 M^{-1}, this would itself be a very impressive and intuitive result. Direct NMR titrations (direct or competitive titrations) could be used to obtain quantitative values, and providing such data would give a much clearer picture of the receptor's selectivity.

Response: We thank the reviewer for their recommendation. Our attempts to perform the NMR titrations at mM concentrations were unsuccessful due to solubility issues (note the extremely large amount of Na^+ needed to be added to the solution).

We therefore performed additional UV–vis titrations (our previous study extended to 500 equiv. Na^+ in Fig S15). Because the weak Na^+ response required very high guest loadings, the titrations in THF were continued until no visible salt precipitation was observed. In contrast to Li^+, Na^+ produced only weak spectral changes at much higher guest concentrations (final [Na+] = 52 mM, ~8000 equiv.). Fitting three independent titrations to a 1:1 binding model gave an apparent association constant of $K_{a(Na+)} = (0.9 \pm 0.3) \times 10^2$ M^{-1} (Fig R1 or Fig. S13 in the manuscript). In response to the reviewer's comment, we also evaluated a higher-order binding model to explore the possibility of Na^+-STBoph sandwich complexations; however, this model resulted in poorly constrained and physically unrealistic fitting parameters. We therefore report the Na^+ response using a 1:1 apparent binding model, while avoiding any claim that Na^+ binding is completely absent.

It is also noteworthy that even our accessed purest commercially available sodium salts (metal assay salts) have subppm levels of Li^+ impurities (see our previous note on $NaBF_4$ impurities, Figure S15), which raises uncertainty in the sodium binding experiment given the extremely large excess of Na^+ required to show a visible response (this point is noted in the caption of Fig. S13). Nevertheless, even when using the largest fitted Na^+ affinity, the measured STBorph–Li^+ and STBorph–Na^+ association constants still differ by more than three orders of magnitude.

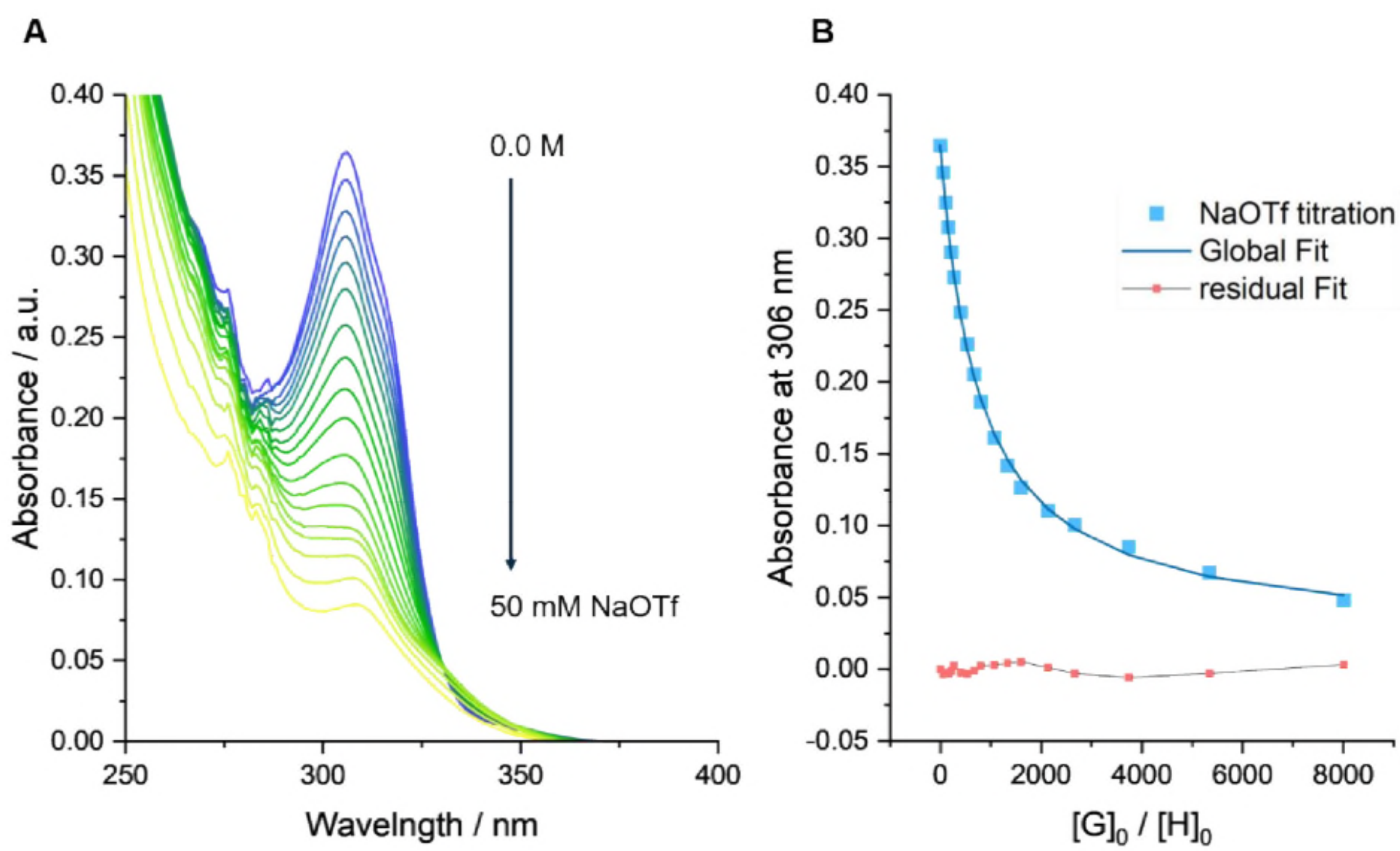

Fig S13. UV–vis titration of **STBorph** with NaOTf. (A) UV–vis spectra of **STBorph** ([**STBorph**] = 10 μM) in THF upon addition of NaOTf from a concentrated THF stock solution at 25 ˚C. The final NaOTf concentration was 50.2 mM. (B) Representative global fitting data for the NaOTf titration, monitored at 306 nm, using a 1:1 binding model. The residuals are shown below the fit. Three independent titrations gave an apparent association constant of $K_a = (0.9 \pm 0.3) \times 10^2$ M^{-1} for Na^+ binding. A higher-order 2:1 binding model was also evaluated for the three independent

Na^+ titrations. However, the apparent fitted constants returned by BindFit were not consistent across the datasets: K_{11} = ~90 M^{-1} (err. ~6%) and K_{21} = ~2.9 × 10^4 M^{-1} (err. ~30%) for titration 1; K_{11} = ~231 M^{-1} (err. ~12%) and K_{21} = ~7.5 × 10^{-7} M^{-1} (err. ~23%) for titration 2; and K_{11} = ~9.4 × 10^{14} M^{-1} (err. >10^8%) and K_{21} = ~4.9 × 10^{18} M^{-1} (err. >10^8%) for titration 3. These values indicate that the 2:1 model is poorly constrained and physically unrealistic under these conditions. Therefore, the Na^+ response was reported using an apparent 1:1 binding model. It is noteworthy that at such high concentrations, even scarce Li^+ impurities (at least 10 ppm in metal assay Na salts), and viscosity changes may contribute to the observed response.

2. While the authors report single crystals of two macrocycles, structural characterization of the Li^+@tBuBorph complex would provide stronger evidence for Li^+ binding and help validate the proposed binding mode.

Response: We agree that a single-crystal X-ray structure of a Li^+-bound borphyrin complex would provide the most direct validation of the proposed binding geometry. We have pursued crystallization of Li^+-bound borphyrin complexes extensively throughout the development of this project. In response to the reviewer's comment, we repeated the crystallization screen using the most promising conditions identified from our previous trials, preparing an additional 96 samples under rigorously dry and inert-atmosphere conditions with several lithium salts and solvent combinations. However, no single crystals of a borphyrin-containing Li^+ complex suitable for X-ray diffraction were obtained; instead, the trials produced free borphyrin crystals or solvated lithium salts, amorphous solids, or samples unsuitable for structural analysis.

We now state this limitation explicitly in the revised manuscript and summarize the crystallization trials in the Supporting Information (Table S3 and Fig. S17). Accordingly, we have revised the wording throughout the manuscript to avoid implying crystallographic confirmation of the Li^+-bound structure. The proposed Li^+-associated binding geometry is now described as being supported by the combined UV–vis, NMR, mass spectrometric, and DFT evidence, and not by direct crystallographic validation.

Table S3. Summary of crystallization trials for Li^+-bound borphyrin complexes, including a focused 96-sample screen performed under dry and inert-atmosphere conditions.

#	Compound	Amount (mg)	Amount (µmol)	Good Solvent	Poor Solvent	Li Salt	Li (equiv)	Li (µmol)
**1-1**	**tBuBorph**	0.5	0.718	$CHCl_3$	—	$LiNTf_2$	3	2.154
**1-2**	**tBuBorph**	0.5	0.718	$CHCl_3$	—	LiBArF	3	2.154
**1-3**	**tBuBorph**	0.5	0.718	$CHCl_3$	—	LiOTf	3	2.154
**1-4**	**tBuBorph**	0.5	0.718	$CHCl_3$	—	LiI	3	2.154
**1-5**	**tBuBorph**	0.5	0.718	THF	—	$LiNTf_2$	3	2.154
**1-6**	**tBuBorph**	0.5	0.718	THF	—	LiBArF	3	2.154
**1-7**	**tBuBorph**	0.5	0.718	THF	—	LiOTf	3	2.154
**1-8**	**tBuBorph**	0.5	0.718	THF	—	LiI	3	2.154
**1-9**	**tBuBorph**	0.5	0.718	$CHCl_3$	Hexanes	$LiNTf_2$	3	2.154
**1-10**	**tBuBorph**	0.5	0.718	$CHCl_3$	Hexanes	LiBArF	3	2.154
**1-11**	**tBuBorph**	0.5	0.718	$CHCl_3$	Hexanes	LiOTf	3	2.154
**1-12**	**tBuBorph**	0.5	0.718	$CHCl_3$	Hexanes	LiI	3	2.154
**1-13**	**tBuBorph**	0.5	0.718	$CHCl_3$	MeCN	$LiNTf_2$	3	2.154
**1-14**	**tBuBorph**	0.5	0.718	$CHCl_3$	MeCN	LiBArF	3	2.154

**1-15**	**tBuBorph**	0.5	0.718	$CHCl_3$	MeCN	LiOTf	3	2.154
**1-16**	**tBuBorph**	0.5	0.718	$CHCl_3$	MeCN	LiI	3	2.154
**1-17**	**tBuBorph**	0.5	0.718	THF	Hexanes	$LiNTf_2$	3	2.154
**1-18**	**tBuBorph**	0.5	0.718	THF	Hexanes	LiBArF	3	2.154
**1-19**	**tBuBorph**	0.5	0.718	THF	Hexanes	LiOTf	3	2.154
**1-20**	**tBuBorph**	0.5	0.718	THF	Hexanes	LiI	3	2.154
**1-21**	**tBuBorph**	0.5	0.718	THF	MeCN	$LiNTf_2$	3	2.154
**1-22**	**tBuBorph**	0.5	0.718	THF	MeCN	LiBArF	3	2.154
**1-23**	**tBuBorph**	0.5	0.718	THF	MeCN	LiOTf	3	2.154
**1-24**	**tBuBorph**	0.5	0.718	THF	MeCN	LiI	3	2.154
**1-25**	**STBorph**	1	0.473	$CHCl_3$	—	$LiNTf_2$	3	1.419
**1-26**	**STBorph**	1	0.473	$CHCl_3$	—	LiBArF	3	1.419
**1-27**	**STBorph**	1	0.473	$CHCl_3$	—	LiOTf	3	1.419
**1-28**	**STBorph**	1	0.473	$CHCl_3$	—	LiI	3	1.419
**1-29**	**STBorph**	1	0.473	THF	—	$LiNTf_2$	3	1.419
**1-30**	**STBorph**	1	0.473	THF	—	LiBArF	3	1.419
**1-31**	**STBorph**	1	0.473	THF	—	LiOTf	3	1.419
**1-32**	**STBorph**	1	0.473	THF	—	LiI	3	1.419
**1-33**	**STBorph**	1	0.473	$CHCl_3$	Hexanes	$LiNTf_2$	3	1.419
**1-34**	**STBorph**	1	0.473	$CHCl_3$	Hexanes	LiBArF	3	1.419
**1-35**	**STBorph**	1	0.473	$CHCl_3$	Hexanes	LiOTf	3	1.419
**1-36**	**STBorph**	1	0.473	$CHCl_3$	Hexanes	LiI	3	1.419
**1-37**	**STBorph**	1	0.473	$CHCl_3$	MeCN	$LiNTf_2$	3	1.419
**1-38**	**STBorph**	1	0.473	$CHCl_3$	MeCN	LiBArF	3	1.419
**1-39**	**STBorph**	1	0.473	$CHCl_3$	MeCN	LiOTf	3	1.419
**1-40**	**STBorph**	1	0.473	$CHCl_3$	MeCN	LiI	3	1.419
**1-41**	**STBorph**	1	0.473	THF	Hexanes	$LiNTf_2$	3	1.419
**1-42**	**STBorph**	1	0.473	THF	Hexanes	LiBArF	3	1.419
**1-43**	**STBorph**	1	0.473	THF	Hexanes	LiOTf	3	1.419
**1-44**	**STBorph**	1	0.473	THF	Hexanes	LiI	3	1.419
**1-45**	**STBorph**	1	0.473	THF	MeCN	$LiNTf_2$	3	1.419
**1-46**	**STBorph**	1	0.473	THF	MeCN	LiBArF	3	1.419
**1-47**	**STBorph**	1	0.473	THF	MeCN	LiOTf	3	1.419
**1-48**	**STBorph**	1	0.473	THF	MeCN	LiI	3	1.419
**2-1**	**STBorph**	1	0.473	Dioxane	—	$LiNTf_2$	3	1.419
**2-2**	**STBorph**	1	0.473	Dioxane	—	LiBArF	3	1.419
**2-3**	**STBorph**	1	0.473	Dioxane	—	LiOTf	3	1.419
**2-4**	**STBorph**	1	0.473	Dioxane	—	LiI	3	1.419
**2-5**	**STBorph**	1	0.473	1,2-DCE	—	$LiNTf_2$	3	1.419
**2-6**	**STBorph**	1	0.473	1,2-DCE	—	LiBArF	3	1.419
**2-7**	**STBorph**	1	0.473	1,2-DCE	—	LiOTf	3	1.419
**2-8**	**STBorph**	1	0.473	1,2-DCE	—	LiI	3	1.419
**2-9**	**STBorph**	1	0.473	Dioxane	Et_2O	$LiNTf_2$	3	1.419
**2-10**	**STBorph**	1	0.473	Dioxane	Et_2O	LiBArF	3	1.419
**2-11**	**STBorph**	1	0.473	Dioxane	Et_2O	LiOTf	3	1.419
**2-12**	**STBorph**	1	0.473	Dioxane	Et_2O	LiI	3	1.419
**2-13**	**STBorph**	1	0.473	Dioxane	Pentane	$LiNTf_2$	3	1.419
**2-14**	**STBorph**	1	0.473	Dioxane	Pentane	LiBArF	3	1.419
**2-15**	**STBorph**	1	0.473	Dioxane	Pentane	LiOTf	3	1.419
**2-16**	**STBorph**	1	0.473	Dioxane	Pentane	LiI	3	1.419
**2-17**	**STBorph**	1	0.473	1,2-DCE	Et_2O	$LiNTf_2$	3	1.419
**2-18**	**STBorph**	1	0.473	1,2-DCE	Et_2O	LiBArF	3	1.419
**2-19**	**STBorph**	1	0.473	1,2-DCE	Et_2O	LiOTf	3	1.419
**2-20**	**STBorph**	1	0.473	1,2-DCE	Et_2O	LiI	3	1.419
**2-21**	**STBorph**	1	0.473	1,2-DCE	Pentane	$LiNTf_2$	3	1.419
**2-22**	**STBorph**	1	0.473	1,2-DCE	Pentane	LiBArF	3	1.419
**2-23**	**STBorph**	1	0.473	1,2-DCE	Pentane	LiOTf	3	1.419
**2-24**	**STBorph**	1	0.473	1,2-DCE	Pentane	LiI	3	1.419

**2-25**	**tBuBorph**	0.5	0.718	Dioxane	—	$LiNTf_2$	3	2.154
**2-26**	**tBuBorph**	0.5	0.718	Dioxane	—	LiBArF	3	2.154
**2-27**	**tBuBorph**	0.5	0.718	Dioxane	—	LiOTf	3	2.154
**2-28**	**tBuBorph**	0.5	0.718	Dioxane	—	LiI	3	2.154
**2-29**	**tBuBorph**	0.5	0.718	1,2-DCE	—	$LiNTf_2$	3	2.154
**2-30**	**tBuBorph**	0.5	0.718	1,2-DCE	—	LiBArF	3	2.154
**2-31**	**tBuBorph**	0.5	0.718	1,2-DCE	—	LiOTf	3	2.154
**2-32**	**tBuBorph**	0.5	0.718	1,2-DCE	—	LiI	3	2.154
**2-33**	**tBuBorph**	0.5	0.718	Dioxane	Et_2O	$LiNTf_2$	3	2.154
**2-34**	**tBuBorph**	0.5	0.718	Dioxane	Et_2O	LiBArF	3	2.154
**2-35**	**tBuBorph**	0.5	0.718	Dioxane	Et_2O	LiOTf	3	2.154
**2-36**	**tBuBorph**	0.5	0.718	Dioxane	Et_2O	LiI	3	2.154
**2-37**	**tBuBorph**	0.5	0.718	Dioxane	Pentane	$LiNTf_2$	3	2.154
**2-38**	**tBuBorph**	0.5	0.718	Dioxane	Pentane	LiBArF	3	2.154
**2-39**	**tBuBorph**	0.5	0.718	Dioxane	Pentane	LiOTf	3	2.154
**2-40**	**tBuBorph**	0.5	0.718	Dioxane	Pentane	LiI	3	2.154
**2-41**	**tBuBorph**	0.5	0.718	1,2-DCE	Et_2O	$LiNTf_2$	3	2.154
**2-42**	**tBuBorph**	0.5	0.718	1,2-DCE	Et_2O	LiBArF	3	2.154
**2-43**	**tBuBorph**	0.5	0.718	1,2-DCE	Et_2O	LiOTf	3	2.154
**2-44**	**tBuBorph**	0.5	0.718	1,2-DCE	Et_2O	LiI	3	2.154
**2-45**	**tBuBorph**	0.5	0.718	1,2-DCE	Pentane	$LiNTf_2$	3	2.154
**2-46**	**tBuBorph**	0.5	0.718	1,2-DCE	Pentane	LiBArF	3	2.154
**2-47**	**tBuBorph**	0.5	0.718	1,2-DCE	Pentane	LiOTf	3	2.154
**2-48**	**tBuBorph**	0.5	0.718	1,2-DCE	Pentane	LiI	3	2.154

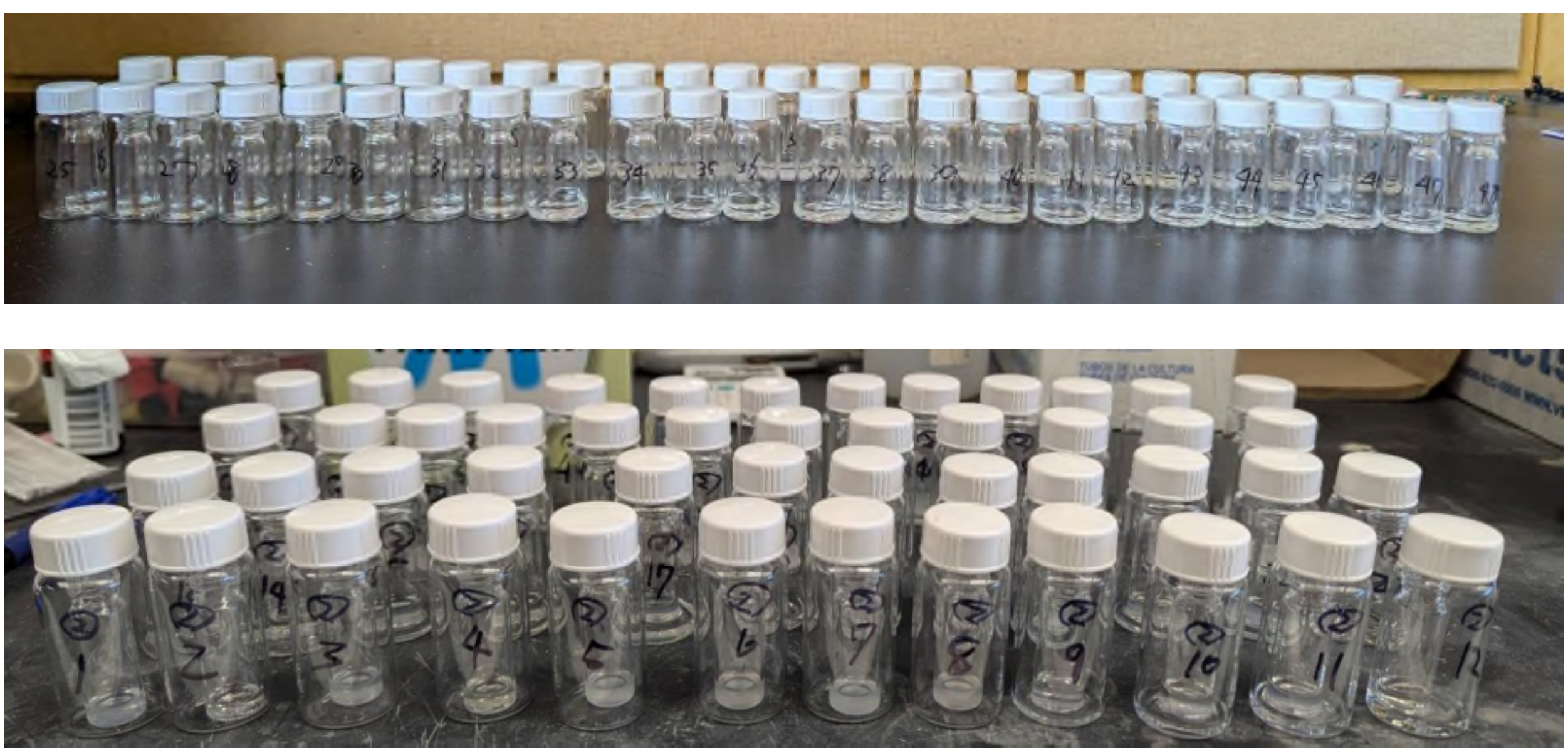

Fig. S17. Photograph of the full 96-sample crystallization screen for Li^+-bound borphyrin complexes prepared under dry and inert-atmosphere conditions.

3. The authors assert that their system offers practical advantages over previous macrocycles: "These systems often require multistep low-yield synthesis, show weak lithium binding affinity, poor selectivity, or dependence on specific salts for effective extraction in either solid-state or solution environments, which have further limited practical applications." However, this

manuscript does not provide sufficient evidence to support a clear superiority in terms of practical applications. To overcome solubility limitations, bulky substituents were introduced, reducing the simplicity of the system. Moreover, boronic esters have inherent stability limitations, particularly in aqueous or extreme pH environments. Without additional data demonstrating that borphyrins are truly more suitable for practical applications than existing systems, the critique of previous work appears overstated.

Response: We appreciate the reviewer's comment. Our intention in including this statement was to provide the current perspective of the field rather than to imply the superiority of our macrocycle. We agree that our original comparison with previously reported macrocyclic receptors was overstated. In the revised Introduction, we have softened this language and removed statements implying that previously reported systems are generally unsuitable for practical applications. Instead, we now state that there remains an opportunity to develop new macrocyclic design motifs that integrate rigid cavities for Li^+, strong Li^+ affinity, high Li^+/Na^+ selectivity, and synthetic accessibility. We have also revised the Abstract, Introduction, Results, and Conclusion to avoid presenting the current borphyrins as an immediately practical or fully recyclable lithium recovery technology and instead focus on presenting the borphyrin architecture as a new motif capable of highly selective Li^+ binding.

4. Regarding potential applications, the "sea salt mimic" extraction is carried out by drying the salts and performing extraction in cyclopentyl methyl ether. This approach differs significantly from direct aqueous-phase extraction and requires additional processing steps, including concentration, solvent handling, and eventual recovery of the receptor. The manuscript should explicitly note these limitations to avoid giving a misleading impression of practical applicability.

Response: We agree with the reviewer and apologize for the ambiguity regarding the *sea-salt* mimic experiment vs. direct aqueous extraction from seawater. In the revised manuscript, we have further clarified that these experiments involve extraction from dried salt mixtures under dry conditions. We have removed any language that could be interpreted as implying direct extraction from seawater, and have reframed the experiment as a demonstration of selective Li^+ recognition in a complex dry-salt environment. The revised text now presents the borphyrins as a proof-of-concept molecular design motif for selective lithium recognition under competitive dry-salt conditions, rather than as a demonstrated process for aqueous lithium recovery.

5. To better illustrate the potential utility of the system, the authors could consider a continuous extraction setup, such as a U-tube or membrane-based experiment, to evaluate whether selective Li^+ transfer from an aqueous or mixed-phase solution can be achieved. Such studies would provide valuable insight into the real-world applicability of borphyrins.

Response: While the continuous extraction, U-tube, membrane-based, or related phase-transfer experiments would provide valuable information about practical lithium transport, the current borphyrin derivatives undergo protodeborylation under protic or aqueous Li^+ release conditions, which prevents straightforward recycling of the receptor. Because of this limitation, we believe that such continuous extraction experiments would be premature within the current scope of borphyrins. We believe that further development of this proof-of-concept platform through the design of new borphyrin derivatives with improved hydrolytic stability and controlled Li^+ release

will be necessary before investigating recyclability and continuous extraction processes.

6. Even as a non-specialist in the field, this reviewer noticed that some relevant literature may be missing. The authors should ensure that prior high-affinity Li^+ receptors and recent reviews are properly cited, to place this work in a more complete context.

Response: We thank the reviewer for pointing out these important references and precedents. We have expanded the Introduction to include other high-affinity Li^+ receptors, such as Cram's spherands, as well as a recent *Coord. Chem. Rev.* article on lithium-selective receptors. Indeed, including the suggested references better places the present borphyrin system in the context of established Li^+-binding macrocycles and ionophores.

Reviewer 2

This manuscript reports a new class of macrocycles, termed borphyrins, featuring a dioxaborole-linked head-to-tail architecture. The synthetic strategy is elegant, operationally simple, and effective, relying on the condensation of a protected boric acid derivative to circumvent protodeborylation. All three macrocycles are rigorously characterized by single-crystal X-ray diffraction. Structurally, these compounds define a rigid cavity reminiscent of a 16-crown-4 motif and exhibit high affinity and selectivity for lithium ions in organic solvents, enabling extraction of lithium from crystalline solids or mixed salt systems into organic phases. Despite the aesthetic appeal of these molecules, several concerns limit the overall impact of the work. The key points are outlined below:

Response:
We thank the reviewer for the positive assessment of the synthetic strategy and structural design of borphyrins. To address the raised concerns, we have revised the manuscript to better define the scope of the work, to discuss protodeborylation and recyclability more critically, and improve the clarity and reproducibility of the binding and extraction experiments.

1. The Introduction emphasizes the importance of lithium extraction from brines or seawater; however, the study focuses exclusively on solid–liquid extraction, which is less directly aligned with this motivation. Moreover, the reported macrocycles are not compatible with aqueous environments and therefore cannot be applied to lithium recovery from water. The authors should revise the Introduction to more accurately frame the significance of solid–liquid extraction, clearly identify the outstanding challenges in this area, and articulate how the present work addresses a specific gap.

Response: We agree that the original Introduction placed too much emphasis on lithium recovery from brines and seawater relative to the experiments presented. In the revised manuscript, we have clarified that the current study does not demonstrate direct aqueous lithium extraction from brines or seawater. Instead, we now present the work as a molecular-recognition study focused on selective Li^+ binding and extraction under dry conditions, including dry lithium salts and dried mixed-salt samples. The Abstract, Introduction, Results, and Conclusion have been revised accordingly, and application-oriented statements have been softened throughout the manuscript.

2. A major limitation of these macrocycles for lithium separation is their susceptibility to protodeborylation under aqueous conditions. This instability restricts their application to lithium capture without a viable pathway for controlled release and recyclability. Given that practical separation technologies require reversible binding, this issue represents a critical weakness. Demonstration of a strategy for receptor regeneration and lithium release would be essential to substantiate the broader applicability of this system. In its current form, this limitation raises concerns regarding the suitability of the work for publication in a high-impact journal such as Nature Communications.

Response: We thank the reviewer for this important comment regarding the applicability of

borphyrins. We fully recognize the limitations of the current borphyrin system within the scope of this manuscript, including its limited aqueous stability and solubility. However, the development of selective lithium ionophores has lagged significantly behind that of receptors for many other ions and molecular guests. We believe that borphyrins introduce a new molecular design motif for selective Li^+ recognition that will stimulate further development of this class of macrocycles and hopefully lead to robust and practically applicable systems.

To clarify the applicability of borphyrin and their importance, we have revised the manuscript to discuss these limitations more explicitly. We now clearly state that hydrolytic stability and controlled Li^+ release into water are key limitations of the present design, and the manuscript no longer presents the current borphyrins as a fully recyclable lithium recovery platform. Instead, they are described as a proof-of-concept molecular design motif for selective Li^+ recognition. While we acknowledge that controlled Li^+ release and receptor recyclability into water important features, we note that the binding of Li^+ to borphyrins is reversible, already demonstrated by competitive binding experiments with 12-crown-4 (Fig. 2C), which is an encouraging foundation for the future development of recyclable borphyrin.

3. For improved clarity and reproducibility, the authors should explicitly report the receptor concentrations used in both the NMR titration experiments (Figure 2C) and the liquid–solid extraction studies.

Response:
We thank the reviewer for pointing out this omission. We have added the relevant concentrations, where applicable, to the figure captions.

Reviewer 3

The manuscript reports the synthesis of a new class of rigid dioxaborole-linked macrocycles ("borphyrins") obtained through a head-to-tail assembly strategy and demonstrates their highly selective Li^+ binding and extraction behaviour. The synthetic concept is clearly original, and the combination of rigid cavity design, strong lithium affinity, and extraction from complex salt mixtures will likely be of interest to the supramolecular and separation chemistry communities. In particular, the scalable synthesis and unusual macrocyclic architecture represent clear strengths of the work. At the same time, several aspects of the study require further clarification before publication in Nat. Commun. While the supramolcular chemistry itself is convincing I find some of the broader claims regarding sustainable Li^+ extraction and practical applicability somewhat overstated based on the current experimental evidence. I therefore recommend major revision!

My major comments and concerns are:

Response:
We thank the reviewer for the constructive assessment of our manuscript and for recognizing the originality of our design. We agree that several broader claims in the original manuscript were originally overstated, and in line with other reviewers' comments, we have strengthened the discussion on quantitative Li^+/Na^+ selectivity, emphasized the absence of direct crystallographic validation for the Li^+-bound state, revised the discussion of Mg/Ca-containing salts, and substantially softened application-oriented claims related to sustainable lithium recovery.

1) Structural evidence for Li^+ encapsulation. A central claim of the manuscript is the selective encapsulation of Li^+ within the rigid borphyrin cavity. However, despite extensive crystallographic characterization of the free borphyrin, no single-crystal X-ray structure of a Li^+-bound complex is presented. The proposed binding mode is currently supported mainly by DFT calculations, UV/Vis titrations, NMR spectroscopy, and mass spectrometry. I appreciate that crystallization of Li^+ complexes can be experimentally difficult and that Li^+ itself is only weakly scattering. Nevertheless, for a study in which the proposed cavity-controlled binding mode represents such a central aspect, the absence of direct structural evidence is noticeable. The authors should either provide a crystallographically characterized Li complex or discuss more explicitly why such a structure could not be obtained. Where possible, the experimental support for the proposed binding model should be strengthened further.

Response: We have pursued crystallization of Li+-bound borphyrin complexes extensively throughout the development of this project. In response to the reviewer's comment, we repeated the crystallization screen using the most promising conditions identified from our previous trials, preparing an additional 96 samples under rigorously dry and inert-atmosphere conditions with several lithium salts and solvent combinations (Fig. S17 & Table S3). However, no single crystals of borphyrin-containing Li^+ complex suitable for X-ray diffraction was obtained. We note that the single-crystal characterization of lithium-containing complexes is a well-recognized challenge due to the high hydration energy and low atomic number of Li^+. Consequently, these crystals are often highly hygroscopic, and even when suitable single crystals are obtained, locating and reliably refining the lithium atoms in the crystal structure can be difficult.

2) Stability and recyclability of the extraction system. The manuscript presents borphyrins as promising systems for sustainable lithium recovery. However, the authors also state that the borphyrin undergoes protodeborylation during lithium recovery, preventing recyclability of the investigated systems. In my opinion, this represents a significant limitation with respect to practical implementation and should be discussed more critically throughout the manuscript. At present, the study still reads more as a proof-of-concept supramolecular extraction system than a practically viable recovery platform.

Response: We agree with the reviewer that the lack of recyclability is a significant limitation of the current system. Although borphyrins show high Li^+ affinity and exceptional selectivity under dry binding and extraction conditions, attempts to release Li^+ into an aqueous phase led to protodeborylation of the borphyrin framework, preventing straightforward recycling of the current derivatives (Fig. S19). We emphasize that the primary contribution of this work is the discovery of a new molecular recognition motif that exhibits ultrahigh Li^+ affinity and unmatched selectivity. Borphyrin provides a promising platform for the rational engineering of future derivatives with improved properties (for example, as we demonstrated for solubility of HBorph vs. STBorph), including enhanced hydrolytic stability and recyclability. Accordingly, we have removed or softened claims related to sustainable lithium recovery and practical implementation throughout the revised manuscript. The revised version now presents borphyrins as a proof-of-concept molecular design motif for selective Li^+ recognition, while identifying hydrolytic stability and controlled Li^+ release as key challenges to be addressed in future designs.

3) Mechanistic understanding of Mg^{2+} interference The decrease in extraction efficiency observed in the presence of $MgSO_4$ and $CaSO_4$ is mainly attributed to desiccant effects. However, this explanation currently remains rather speculative and is not sufficiently supported experimentally. This point is particularly important because Mg/Li separation is one of the central challenges in lithium recovery from brines and seawater. A more detailed mechanistic discussion would therefore strengthen the manuscript considerably. Additional control experiments or discussion addressing possible competitive coordination, ion pairing, precipitation effects, or solvent-dependent behaviour would help clarify this issue.

Response:
We thank the reviewer for this important point. Our initial ICP measurements showed that the presence of Mg and Ca salts in the salt mixture decreases the amount of Li extracted (at a given extraction time), while none of the other cations were detectably co-extracted into the organic phase (Fig. 3a; Ca and Mg < 0.1%). This observation rules out competitive coordination or co-extraction of Mg^{2+} and Ca^{2+} as the primary cause of the reduced lithium extraction.
Furthermore, previous studies have shown that residual and adventitious water can facilitate solid-state extraction processes (Smith and coworkers, *Inorg. Chem.*, 2004). We therefore believe that the hygroscopic (desiccant) effect of the Mg and Ca salts is the most plausible explanation for the reduced extraction efficiency. However, to avoid assigning the effect to a single mechanism, we have expanded the discussion in the revised manuscript to acknowledge that the reduced extraction likely reflects the greater complexity of the mixed solid-salt environment, where hydration, ion pairing, salt accessibility, and other solid-state effects may all influence the extraction process.

4) Scope of the application-oriented claims. The chemistry is interesting and the selectivity results are certainly noteworthy. However, some statements regarding sustainable lithium recovery and practical extraction applications appear somewhat ahead of the demonstrated robustness of the system. In particular, the absence of recyclability and the still relatively preliminary extraction studies suggest that the practical implications should be discussed more cautiously.

Overall, I find the chemistry original and scientifically interesting, and I believe the work could become suitable for Nat. Commun. after substantial revision. However, in its current form I am not yet fully convinced that the mechanistic and structural evidence supporting the central claims reaches the level expected for publication in the journal. In particular the lack of direct strutural validation of the Li-bound state and the still limited mechanistic understanding of the extraction behaviour under competitive conditions weaken the broader application-oriented conclusions.

Response: We thank the reviewer for the constructive criticism regarding the wording and claims of the borphyrin application. We have therefore revised the Abstract, Introduction, Results, and Conclusion to remove statements implying direct aqueous lithium extraction from seawater or brines, sustainable lithium recovery, or a fully developed recyclable extraction technology. The revised manuscript now emphasizes the original borphyrin macrocyclic architecture, strong Li^+ affinity, a quantified >3000-fold difference between the measured STBorph–Li^+ and STBorph–Na^+ association constants, and proof-of-concept selective Li^+ recognition under dry competitive salt conditions, while acknowledging the limitations of the present study.

Response to Reviewers

Reviewer 1

The revision has addressed several technical concerns by providing additional Na^+ binding data, clarifying the extraction conditions, and moderating the application-oriented claims. These revisions improve the rigor and accuracy of the manuscript. However, they do not substantially change my original assessment regarding the overall significance of the work.
Importantly, the original manuscript was motivated and positioned from a practical lithium extraction perspective. In the revised version, however, the authors have explicitly acknowledged that the present system should only be regarded as a proof-of-concept molecular recognition platform rather than a practically relevant lithium recovery technology. This substantially changes the significance of the work. Once the practical applicability is removed from the central claim, the manuscript is essentially reduced to the development of a new Li^+-binding macrocycle. In this context, the key question becomes what conceptual or functional advance this receptor provides over established high-affinity Li^+ receptors, particularly classical spherands. Although the borphyrin architecture is structurally elegant and synthetically interesting, I do not believe the current manuscript demonstrates a sufficiently significant conceptual advance in molecular recognition. The revised manuscript also explicitly acknowledges important limitations of the present system, including poor hydrolytic stability, lack of recyclability, and the inability to perform continuous or aqueous extraction. Therefore, unless these fundamental limitations can be addressed, I believe this work remains at the level of introducing a new Li^+-binding macrocycle rather than establishing a genuinely impactful advance in lithium recognition or separation. While the chemistry is interesting, I am still not convinced that the conceptual novelty and overall significance reach the level expected for publication in Nature Communications.

Response:
We thank the reviewer for careful consideration and reassessment of our work. Of course, the question of novelty is always open to debate and we respect the reviewer's opinion, which is different from ours and the other referees. However, we respectfully disagree that once separated from its potential application, the present work is simply "…reduced to the development of a new Li^+-binding macrocycle".

Despite nearly six decades of research since Cram's spherands, the development of Li^+ ionophores that simultaneously combine synthetic accessibility, high affinity, and exceptional selectivity over competing ions remains an important challenge in molecular recognition. Classical spherands (as the only ionophore with a higher Li-binding constant than borphyrin) have very low overall yields (~2% in the originally reported systems), and while having extraordinarily reported $K_a(Li^+) > 7 \times 10^{16}\ M^{-1}$, they also bind Na^+ strongly ($K_a(Na^+) \sim 10^{14}\ M^{-1}$), with a Li^+/Na^+ selectivity of only 600 under those measurement conditions. The borphyrins reported here are obtained in >80% isolated yield while exhibiting Li^+ association constants of $\sim 10^6\ M^{-1}$ (see Table S1 for comparison with other previously reported Li^+ ionophores), and it is >10,000-fold weaker binding of Na^+ and no detectable competitive binding by the other investigated ions.

We therefore believe the advance of borphyrins is not simply another Li^+ ionophore, but a unique motif with the combination of strong Li^+ binding, exceptional ion specificity, >80% isolated synthetic yield, and a rigid, preorganized cavity that retains its geometry upon Li^+ binding

and intrinsically disfavors sandwich complexation. Although hydrolytic stability and recyclability remain important targets for future derivatives of borphyrins, we believe this work establishes borphyrins as a conceptually distinct Li^+ recognition platform rather than simply another high-affinity macrocycle.

Reviewer 2

The authors have adequately addressed my previous comments by moderating their claims and more clearly acknowledging the current limitations of their receptors. I no longer have any objections to the publication of this manuscript and recommend its acceptance.

Response:
We thank the reviewer for carefully reassessing our revised manuscript and for recognizing the changes made in response to the previous comments. We are grateful for the reviewer's recommendation for acceptance.

Reviewer 3

1) The authors have carried out a comprehensive revision and responded constructively to the major concerns I raised in my previous review. In particular, the extensive crystallization experiments for Li+-bound borphyrin complexes are now documented, and the manuscript correctly notes that the proposed Li+-associated geometry is supported more by the combined spectroscopic, mass spectrometric, and computational evidence than by direct crystallographic validation. I consider this to be an appropriate and transparent handling of the structural limitation.

Response:
We thank the reviewer for the careful reassessment of our manuscript and for recognizing our efforts to address this important structural limitation. We have retained the revised discussion clearly distinguishing the combined spectroscopic, mass spectrometric, and computational evidence from direct crystallographic validation.

2) The lack of reusability is now also discussed much more clearly. The revised manuscript explicitly identifies protodeborylation during aqueous or protic Li+ release as a limitation of the present derivatives and appropriately presents the system as a "proof-of-concept" platform for molecular recognition rather than as an immediately applicable or sustainable technology for lithium recovery.

Response:
We thank the reviewer for this positive assessment. We have retained this proof-of-concept framing and the explicit discussion of protodeborylation and the lack of reusability as important limitations of the present borphyrin system.

3) The origin of the reduced extraction efficiency in Mg^{2+}- and Ca^{2+}-containing mixtures remains mechanistically unclear. The absence of measurable co-extraction of these ions does not, in itself, rule out transient coordination or indirect interfacial and solid-state effects. However, the revised manuscript no longer attributes the effect to a single mechanism and now appropriately presents it as a limitation of the extraction system under complex solid-salt conditions. I therefore do not consider additional mechanistic experiments to be essential for publication.

Response:
We thank the reviewer for this assessment. We agree that the origin of the reduced lithium extraction efficiency in the presence of Mg^{2+} and Ca^{2+} cannot be assigned to a single mechanism based on the present data. We have therefore retained the cautious discussion introduced in the previous revision and do not make a specific mechanistic assignment for this effect.

4) Two statements in the abstract must nevertheless be corrected. First, the description of the lithium affinity as "nanomolar" contradicts the reported association constants of 1.8×10^6 and 3.9×10^5 M^{-1}, which correspond to dissociation constants of approximately 0.56 and 2.6 µM, respectively. The affinity should therefore be described as strong or as falling within the submicromolar to micromolar range, rather than as nanomolar.

Response:
We thank the reviewer for identifying this error. We agree that the term "nanomolar affinity" was not appropriate based on the measured association constants. We have therefore revised the Abstract to describe the borphyrin as exhibiting **"strong affinity for lithium."** The revised sentence now reads: "This molecule exhibits strong affinity for lithium and high selectivity over competing cations under dry solid-liquid extraction conditions, owing to its narrow binding cavity and planar macrocyclic core."

5) Second, the statement that there is "no interference from competing cations" is not supported by the extraction results, since the addition of alkaline earth salts reduces the extraction efficiency from approximately 98.6% to approximately 50%. Although these ions are not co-extracted to any significant extent, they clearly influence lithium extraction. This sentence should be revised to distinguish between selectivity and co-extraction based on the observed reduction in extraction efficiency.

Response: We agree with the reviewer and have revised the Abstract to distinguish between the absence of measurable co-extraction of competing cations and their effect on lithium extraction efficiency. Specifically, we removed the statement that there is "no interference from competing cations". The revised text reads: "This molecule exhibits strong affinity for lithium and high selectivity over competing cations under dry solid-liquid extraction conditions, owing to its narrow binding cavity and planar macrocyclic core".
